# Geographical Differences and Temporal Improvements in Forced Expiratory Volume in 1 Second of Preterm-Born Children: A Systematic Review and Meta-analysis

**DOI:** 10.1001/jamapediatrics.2022.1990

**Published:** 2022-06-27

**Authors:** Sarah J. Kotecha, James T. D. Gibbons, Christopher W. Course, Emily E. Evans, Shannon J. Simpson, W. John Watkins, Sailesh Kotecha

**Affiliations:** 1 Department of Child Health, Cardiff University School of Medicine, Cardiff, United Kingdom; 2 Children’s Lung Health, Wal-yan Respiratory Research Centre, Telethon Kid’s Institute, Perth, Australia; 3 Department of Respiratory Medicine, Perth Children’s Hospital, Perth, Australia; 4 Department of Paediatrics, Cardiff and Vale University Health Board, Cardiff, United Kingdom; 5 School of Allied Health, Curtin University, Perth, Australia

## Abstract

**Importance:**

Although preterm birth is associated with later deficits in lung function, there is a paucity of information on geographical differences and whether improvements occur over time, especially after surfactant was introduced.

**Objective:**

To determine deficits in percentage predicted forced expiratory volume in 1 second (%FEV_1_) in preterm-born study participants, including those with bronchopulmonary dysplasia (BPD) in infancy, when compared with term-born control groups.

**Data Sources:**

Eight databases searched up to December 2021.

**Study Selection:**

Studies reporting spirometry for preterm-born participants with or without a term-born control group were identified.

**Data Extraction and Synthesis:**

Data were extracted and quality assessed by 1 reviewer and checked by another. Data were pooled using random-effects models and analyzed using Review Manager and the R metafor package.

**Main Outcomes and Measures:**

Deficits in %FEV_1_ between preterm-born and term groups. Associations between deficits in %FEV_1_ and year of birth, age, introduction of surfactant therapy, and geographical region of birth and residence were also assessed.

**Results:**

From 16 856 titles, 685 full articles were screened: 86 with and without term-born control groups were included. Fifty studies with term controls were combined with the 36 studies from our previous systematic review, including 7094 preterm-born and 17 700 term-born participants. Of these studies, 45 included preterm-born children without BPD, 29 reported on BPD28 (supplemental oxygen dependency at 28 days), 26 reported on BPD36 (supplemental oxygen dependency at 36 weeks’ postmenstrual age), and 86 included preterm-born participants. Compared with the term-born group, the group of all preterm-born participants (all preterm) had deficits of %FEV_1_ of −9.2%; those without BPD had deficits of −5.8%, and those with BPD had deficits of approximately −16% regardless of whether they had BPD28 or BPD36. As year of birth increased, there was a statistically significant narrowing of the difference in mean %FEV_1_ between the preterm- and term-born groups for the all preterm group and the 3 BPD groups but not for the preterm-born group without BPD. For the all BPD group, when compared with Scandinavia, North America and western Europe had deficits of −5.5% (95% CI, −10.7 to −0.3; *P* = .04) and −4.1% (95% CI, −8.8 to 0.5; *P* = .08), respectively.

**Conclusions and Relevance:**

Values for the measure %FEV_1_ were reduced in preterm-born survivors. There were improvements in %FEV_1_ over recent years, but geographical region had an association with later %FEV_1_ for the BPD groups.

## Key Points

**Question:**

What is the lung function, as indicated by percentage predicted forced expiratory volume in 1 second (%FEV_1_), of preterm-born participants, including those who had bronchopulmonary dysplasia (BPD) in infancy, when compared with term-born participants?

**Findings:**

This systematic review and meta-analysis showed decreased %FEV_1_ in preterm-born survivors, especially in the BPD group, when compared with children in the term-born control group. Values for %FEV_1_ improved over time, but geographical region affected later %FEV_1_ in the BPD group.

**Meaning:**

Although decreased %FEV_1_ values improved over time, geographical region affected %FEV_1_ in the BPD group.

## Teaser

This systematic review and meta-analysis compares data for percentage predicted forced expiratory volume in 1 second (%FEV_1_) among preterm-born participants, including those with bronchopulmonary dysplasia in infancy, with term-born control groups.

## Introduction

As survival rates of preterm-born babies improve, especially among those born at extremes of prematurity, longer-term outcomes to ensure good quality of life are increasingly important. Our previous systematic review,^1^ which included publications up to 2011, demonstrated that survivors of preterm-birth, with and without the neonatal chronic lung disease called bronchopulmonary dysplasia (BPD), have deficits of 7.2% and 16.2%, respectively, for forced expiratory volume in 1 second (FEV_1_) compared with term-born controls. However, the previous review included cohorts predominantly born before the introduction of surfactant. Subsequent individual studies of preterm-born cohorts in the postsurfactant era also show deficits,^2,3,4^ but whether the degree of deficits is comparable with the presurfactant era is unclear. It is also unclear whether there are geographical differences or whether the deficits change with age. The previous systematic review was largely limited to the diagnosis of BPD defined as supplemental oxygen dependency at 28 days of age (BPD28) rather than the potentially more useful outcome of supplemental oxygen dependency at 36 weeks’ corrected gestation (BPD36).

Therefore, we updated the previous systematic review for the preterm group with and without BPD28 or BPD36 to (1) address if deficits in %FEV_1_ continue to be observed in those born preterm, including those who had either BPD28 or BPD36 in infancy; (2) describe change in %FEV_1_ over time; (3) report if %FEV_1_ changes with age; (4) identify changes in %FEV_1_ before and after introduction of surfactant; and (5) identify geographical differences where practices in the management of preterm-born infants may vary, when compared with term-born infants.

## Methods

The previous search strategy was used again^1^ (eAppendix 1 in the Supplement). Articles identified previously up to October 2011 using the databases CINAHL, Embase, HMIC Health Management Consortium, Medline, Scopus, OpenSIGLE, and Web of Knowledge (Science Citation Index Expanded, Social Science Citation Index, ISI proceedings) were searched from 2011 to December 2021. The review was not registered. Preferred Reporting Items for Systematic Reviews and Meta-analyses (PRISMA) reporting guidelines were followed.^5^ Ethics approval was not required because only published data were used.

### Eligibility Criteria and Outcome Measures

Studies that compared %FEV_1_ or FEV_1_ *z* scores in later life for preterm-born infants with and without BPD with a contemporaneous term-born population were eligible for inclusion. Prematurity was defined as birth at less than 37 weeks’ gestation and term as gestation of 37 weeks or longer. If results were not presented in either of those formats, or where results for a mixed preterm- and term-born group were presented, the authors were contacted for clarification to enable data inclusion. As previously, we included studies recruiting participants based on their birth weight; however, studies had to report %FEV_1_ and gestation, ie, for preterm infants. Bronchopulmonary dysplasia was defined as BPD28 or BPD36. Studies in all languages and from all countries were included.

The main outcome measure was mean %FEV_1_. Associations between deficits in %FEV_1_ and year of birth, age, introduction of surfactant therapy, and geographical region of birth and residence were also assessed.

### Study Selection

Each reference title and, where available, abstract was independently screened by 2 of 4 reviewers (S.J.K., J.T.D.G., C.W.C., and E.E.E.) using the inclusion criteria given in the protocol (eAppendix 1 in the Supplement). Full manuscripts were obtained for titles and abstracts that met the inclusion criteria. Two reviewers then screened the full manuscripts, and 1 (S.K.) mediated any disagreements.

### Data Collection Process

Data were manually extracted by a reviewer (S.J.K. or J.T.D.G.) and entered into a database (REDCap; Telethon Kids Institute); data were checked for accuracy by another (C.W.C. or E.E.E.).^6,7^ One of us (S.J.K.) contacted the authors if the data extraction was not possible (eg, combined data). For multiple publications of the same cohorts, 2 of us (S.K. and S.J.K.) reviewed each article to include the one with the most comprehensive data. For studies including a term-born control group, preterm and term data were entered for meta-analyses and meta-regression.

### Assessment of Study Quality and Risk of Bias

We used the same methodology as previously to assess the study quality (eAppendix 2 in the Supplement). Each study was assessed for quality by 2 reviewers (J.T.D.G. or S.J.K.) and checked by a different reviewer (C.W.C. or E.E.E.). The minimum and maximum possible scores were 6 and 20.

### Statistical Analysis

Review Manager version 5.4 and the R package metafor version 3.0-2^8^ were used to perform statistical analysis. *P* values less than .05 were considered significant. Medians were converted to means using the method of Wan et al.^9^ Data presented graphically were extracted independently by 2 reviewers. Data presented for different groups of preterm-born or term-born children in 1 article were combined where appropriate using Review Manager. We converted *z* scores of FEV_1_ to percentage predicted FEV_1_ by using graphical relationships between *z* scores and %FEV_1_ derived from local cohorts of the Avon Longitudinal Study of Parents and Children (ALSPAC),^10^ Respiratory Health Outcomes in Neonates (RHiNO),^11^ and West Australian Lung Health in Prematurity (WALHIP).^12^

Data from the previous systematic review^1^ and the current update were combined. Because heterogeneity was high in the majority of the forest plots, we used random effects to provide a pooled estimate of the mean difference in %FEV_1_ between preterm-born and term-born participants. The following groups were compared with reported term-born controls:

- All preterm-born participants (including those with and without BPD).
- Preterm-born participants without BPD.
- All-BPD group (including those with BPD28 and BPD36).
- BPD28.
- BPD36.

Studies without a term-born control group were excluded but are given as descriptive results. Studies that presented FEV_1_ results not expressed as percentage predicted or *z* scores (eg, raw values) were excluded unless authors provided further information. If the results for %FEV_1_ or *z* scores were not presented as means (or median) and SD (or ranges, IQRs), they were excluded unless conversion to means and SD was possible. Publication bias was investigated using a funnel plot that plotted effect size against standard error.

To examine the association of factors of interest with the difference in %FEV_1_ between each group above and term-born infants, meta-regressions were conducted. For each comparison, %FEV_1_ was modeled on the individual factors for just the preterm-born group, the term-born group, and then the difference between the preterm and term groups. For each of the 5 groups above, we investigated the association between the mean difference in %FEV_1_ between the preterm-born participants and term-born participants and 4 factors: year of birth, age at time of lung function, surfactant usage, and geographical region of birth or residence (region).

In studies recruiting participants over a range of ages or years of birth, the midpoint was used. Because surfactant use was not often reported, we assumed that those preterm-born participants born in 1990 or earlier did not receive routine surfactant and that surfactant use was gradually introduced for those born in 1993 or later.^13,14,15,16^ Geographical region was defined from the region of birth or residence or the geographical location of the majority of authors if no such information was stated. Given the several favorable outcomes from the Scandinavian countries for several perinatal conditions, Europe was classified into western Europe, eastern Europe, and Scandinavia to enable geographical comparison between the different regions, with Scandinavia as the reference population.

## Results

### Study Selection

The current searches identified 16 856 titles and abstracts; after screening, 685 full articles were identified, and 86 met the inclusion criteria (eFigure 1 in the Supplement). Of these 86 articles, 50^4,12,17,18,19,20,21,22,23,24,25,26,27,28,29,30,31,32,33,34,35,36,37,38,39,40,41,42,43,44,45,46,47,48,49,50,51,52,53,54,55,56,57,58,59,60,61,62,63,64,65^ were combined with the 36 studies from the previous systematic review,^66,67,68,69,70,71,72,73,74,75,76,77,78,79,80,81,82,83,84,85,86,87,88,89,90,91,92,93,94,95,96,97,98,99,100,101,102^ resulting in 86 included studies representing 7094 preterm-born and 17 700 term-born participants (eTables 4, 5, and 6 in the Supplement). Some studies were included in more than 1 analysis. Eighty-six studies compared all preterm-born participants (including some with BPD) with term controls.^4,12,17,18,19,20,21,22,23,24,25,26,27,28,29,30,31,32,33,34,35,36,37,38,39,40,41,42,43,44,45,46,47,48,49,50,51,52,53,54,55,56,57,58,59,60,61,62,63,64,65,66,67,68,69,70,71,72,73,74,75,76,77,78,79,80,81,82,83,84,85,86,87,88,89,90,91,92,93,94,95,96,97,98,99,100^ Forty-five studies compared a preterm-born group who did not have BPD^4,12,17,18,24,26,30,32,33,36,38,39,41,43,44,47,50,51,52,57,58,60,63,64,65,66,68,79,83,84,85,86,87,88,89,90,91,92,93,94,95,96,98,99,100^; 55 compared an all BPD group,^4,12,17,18,20,24,25,26,30,31,32,33,36,38,39,41,42,43,44,45,47,50,51,52,57,60,63,64,65,66,68,79,80,81,82,83,84,85,86,87,88,89,90,91,92,93,94,95,96,97,98,99,100,101,102^ 29 with a BPD28 group^12,18,24,25,26,31,32,38,41,45,47,50,80,81,82,83,84,85,88,91,94,96,97,98,99,100,101,102^; and 26 with a BPD36 group.^4,17,20,26,30,36,39,41,43,45,51,57,60,63,64,65,66,68,79,86,87,89,90,92,93,95^

### Study Characteristics

The characteristics of the studies included in the previous systematic review have already been reported.^1^ Included participants were born between 1961 and 2017. Gestation at birth ranged from 22 to 36 weeks and age from approximately 3 to 52 years for the preterm-born participants. The rates of ventilation and reported surfactant administration varied widely (eTables 4, 5, and 6 in the Supplement).

### Risk of Bias Across Studies and Publication Bias

The mean quality scores for the 86 studies was 13.5 and ranged from 6 to 19 (eTables 4, 5, and 6 in the Supplement). Many studies did not provide description for some domains, so computing a score for potential risk of bias was not possible for these domains. Publication bias was investigated and only observed in the all preterm-born group (eFigure 6 in the Supplement).

### Synthesis of Results

**Figure 1.**
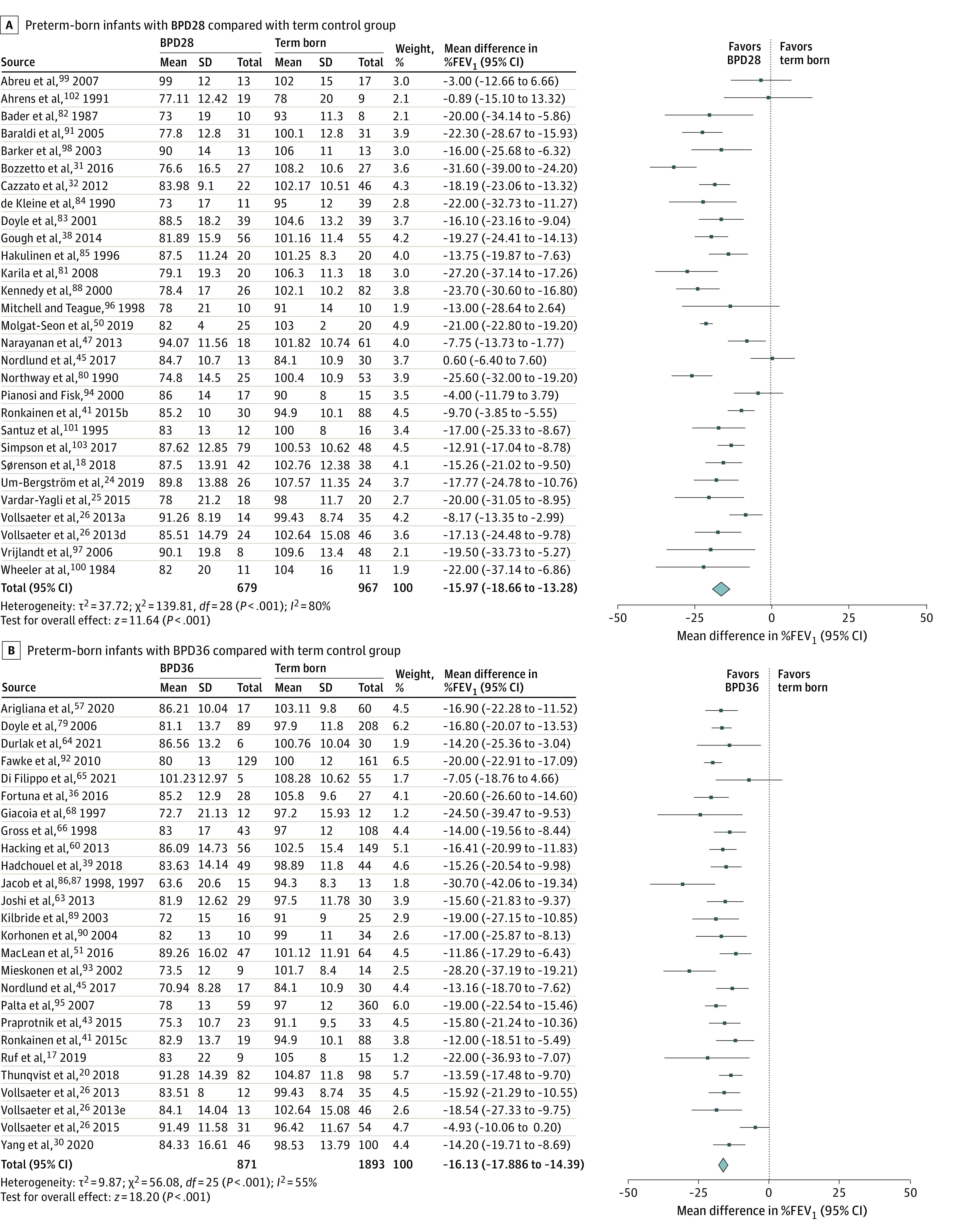
Percentage Predicted Forced Expiratory Volume in 1 Second (%FEV_1_) for All Preterm-Born Participants With Bronchopulmonary Dysplasia Defined as Supplemental Oxygen Dependency at Age 28 Days (BPD28) and 36 Weeks’ Postmenstrual Age (BPD36) Compared With Term-Born Control Group

The comparisons between the preterm- and term-born groups are shown in Figure 1; eFigures 2, 3, and 4 in the Supplement; and Table 1. A mean difference for %FEV_1_ of −9.2% (95% CI, −10.4% to −8.0%) was noted when all preterm-born participants, including those with BPD, were compared with term-born controls (eFigure 2 in the Supplement). The comparison between the preterm-born group without BPD and the term-born group showed a mean difference of −5.8% (95% CI, −7.1% to −4.5%) in %FEV_1_ (eFigure 3 in the Supplement). The comparisons between the BPD and term-born groups showed greater but largely similar mean differences in %FEV_1_ of −15.9% (95% CI, −17.6% to −14.2%), −16.0% (95% CI, −18.7% to −13.3%), and −16.1% (95% CI, −17.9% to −14.4%) for the all BPD (eFigure 4 in the Supplement), BPD28 (Figure 1A), and the BPD36 groups (Figure 1B), respectively. All differences between the preterm and term control groups were statistically significant (all *P* < .001).

**Table 1.** Results of Meta-analyses of %FEV_1_ of the Different Preterm-Born Groups Compared With a Term-Born Control Group

Group compared with term-born participants	No. of studies	No. of preterm-born participants	No. of term-born participants	Mean difference, % (95% CI)	*P* value	*I^2^* ^a^
All preterm-born participants	86	7094	17 700	−9.2 (−10.4 to −8.0)	<.001	87
Preterm-born, no BPD	45	2133	2562	−5.8 (−7.1 to −4.5)	<.001	66
Preterm-born, BPD	55	1736	2827	−15.9 (−17.6 to −14.2)	<.001	76
Preterm-born, BPD28	29	679	967	−16.0 (−18.7 to −13.3)	<.001	80
Preterm-born, BPD36	26	871	1893	−16.1 (−17.9 to −14.4)	<.001	55

Abbreviations: BPD, bronchopulmonary dysplasia; BPD28, BPD defined as supplemental oxygen dependency at 28 days of age; BPD36, BPD defined as supplemental oxygen dependency at 36 weeks’ postmenstrual age; %FEV_1_, percentage predicted forced expiratory volume in 1 second.

^a^ All *P* values for heterogeneity test results were <.001.

### Additional Analysis

#### Year of Birth

**Figure 2.**
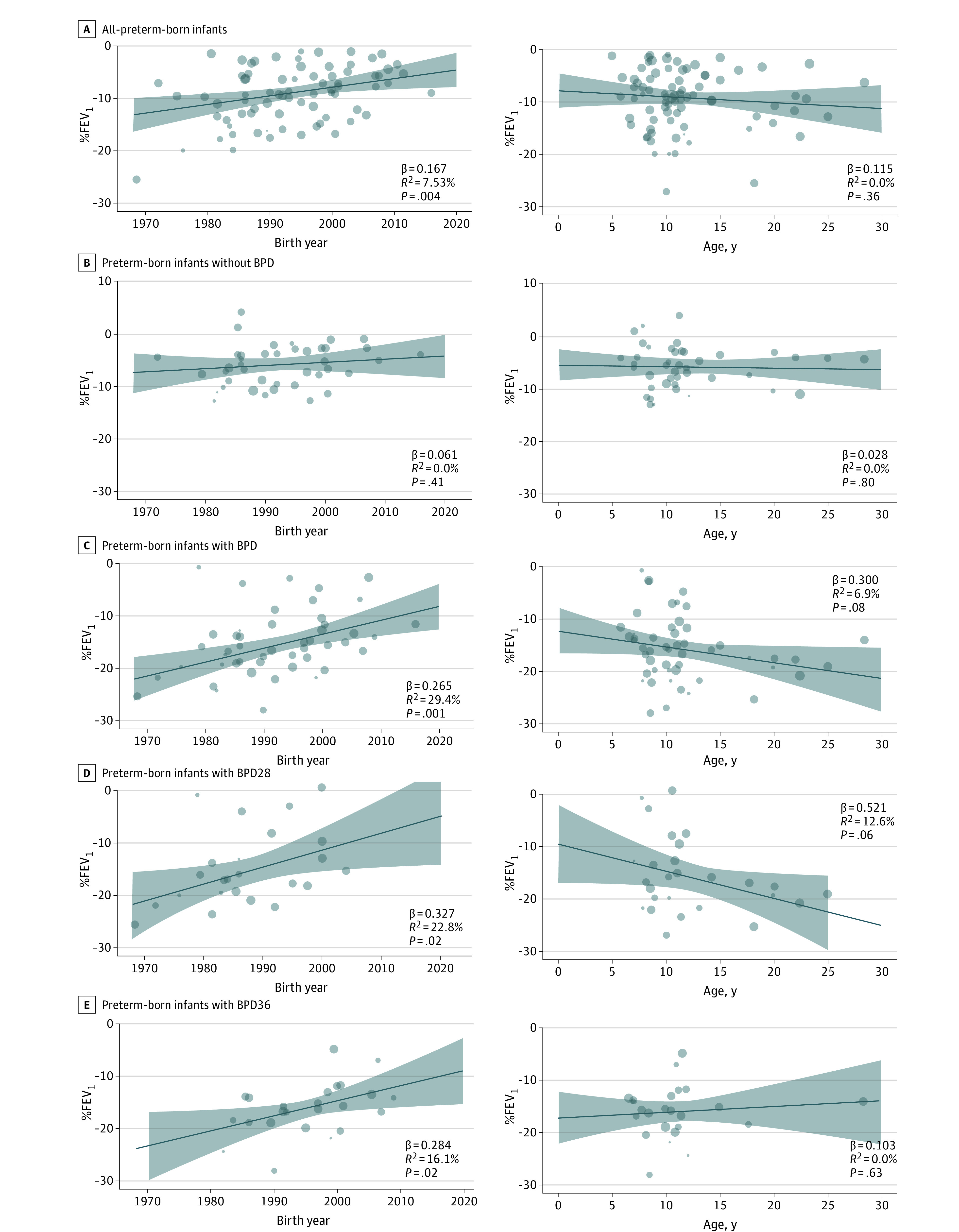
Differences in Percentage Predicted Forced Expiratory Volume in 1 Second (%FEV_1_) Between Preterm-Born Groups and Term-Born Control Groups by Birth Year and Age BPD indicates bronchopulmonary dysplasia; BPD28, BPD defined as supplemental oxygen dependency at 28 days of age; BPD36, BPD defined as supplemental oxygen dependency at 36 weeks’ postmenstrual age.

There was a statistically significant decrease in the difference for mean %FEV_1_ between the preterm- and term-born groups for the all preterm and the 3 BPD groups. However, this was not the case for the preterm-born group without BPD as year of birth increased (Figure 2).

#### Age at Time of Lung Function Testing

Age at time of spirometry did not have a significant association with the difference between the preterm- and term-born groups in mean %FEV_1_ for any of the groups (Figure 2). Although the mean difference between the BPD28 and term-born groups appeared to be associated with an increase, albeit not a statistically significant one, %FEV_1_ increased with age for the term-born group, and %FEV_1_ in the BPD28 group did not decline with age (eFigure 5 in the Supplement).

#### Surfactant Usage

Surfactant usage, taken with several other improvements in managing sick newborn preterm infants over time, resulted in improvements in the difference between the preterm- and term-born groups for mean %FEV_1_ for the all BPD, BPD28, and BPD36 groups but not for all-preterm group or for the preterm-born group without BPD (Table 2 and eTable 1 in the Supplement). These results should be treated with caution because surfactant usage was defined crudely as being introduced in or after 1993 and not being generally available before or in 1990.

**Table 2.** Difference in FEV_1_ Between Preterm-Born and Term-Born Groups Modeled on Surfactant (≤1990 vs ≥1993)

Group compared with term-born participants	No. of studies	β (95% CI)	*P* value
All preterm-born participants	70	2.638 (0.204 to 5.073)	.03
Preterm-born, no BPD	37	0.813 (−1.774 to 3.400)	.54
Preterm-born, BPD	45	4.907 (1.611 to 8.204)	.004
Preterm-born, BPD28	22	5.864 (0.257 to 11.472)	.04
Preterm-born, BPD36	22	4.775 (0.373 to 9.178)	.04

Abbreviations: BPD, bronchopulmonary dysplasia; BPD28, BPD defined as supplemental oxygen dependency at 28 days of age; BPD36, BPD defined as supplemental oxygen dependency at 36 weeks’ postmenstrual age; %FEV_1_, percentage predicted forced expiratory volume in 1 second.

#### Geographical Region of Birth or Residence

For preterm-born participants without BPD, similar degrees of differences were seen between the preterm-born and term-born groups for all geographical regions, with the largest differences observed by the Australasian studies and smallest by the 1 South American study (Table 3). Because Scandinavia has the lowest morbidity for many perinatal conditions, we compared western Europe, North America, and Australasia with Scandinavia as the reference region (since sufficient numbers of participants were available for study). There were no statistical differences for the preterm without BPD group between these geographical regions (eTables 2 and 3 in the Supplement). For the preterm with BPD (both BPD28 and BPD36) group, the difference between the preterm and term groups ranged from −3% for the 1 South American study and −18.1% for North America (Table 3). When North America, western Europe, and Australasia were compared with Scandinavia as the reference region, differences for %FEV_1_ between the preterm group with BPD and term group were −5.5% (95% CI, −10.7 to −0.3; *P* = .04) for North America, −4.1% (95% CI, −8.8 to 0.5; *P* = .08) for western Europe, and −3.9 (95% CI, −9.8 to 2.1; *P* = .20) for Australasia.

**Table 3.** %FEV_1_ by Geographical Region for Preterm-Born Population With and Without BPD Compared With Term-Born Control Group

	No. of studies	No. of preterm-born participants	No. of term-born participants	Mean difference (95% CI)	*P* value	Heterogeneity	
						*I^2^*	*P* value
**Preterm without BPD**							
Europe	26	924	1162	−5.5 (−6.9 to −4.0)	<.001	30	.07
Western Europe	15	658	723	−6.5 (−8.4 to −4.6)	<.001	31	.12
Eastern Europe	2	55	63	−2.59 (−6.7 to 1.5)	.22	0	.38
Scandinavia	8	168	314	−5.0 (−7.1 to −2.8)	<.001	0	.45
North America	11	488	728	−5.5 (−8.6 to −2.4)	<.001	80	<.001
South America	1	10	17	−2.0 (−13.2 to 9.2)	.73	NA	NA
Asia	1	30	29	−4.1 (−11.0 to 2.8)	.24	NA	NA
Australasia	6	681	626	−6.9 (−9.4 to −4.4)	<.001	67	.01
**Preterm with BPD**							
Europe	33	994	1401	−15.4 (−17.7 to −13.1)	<.001	77	<.001
Western Europe	21	626	895	−16.8 (−19.9 to −13.7)	<.001	78	<.001
Eastern Europe	2	29	63	−15.5 (−20.4 to −10.6)	<.001	0	<.001
Scandinavia	10	339	443	−12.4 (−15.6 to −9.2)	<.001	68	.001
North America	13	321	734	−18.1 (−21.3 to −14.9)	<.001	68	<.001
South America	1	13	17	−3.00 (−12.7 to 6.6)	.54	NA	NA
Asia	1	55	29	−11.7 (17.8 to −5.7)	<.001	NA	NA
Australasia	6	335	626	−16.2 (−18.7 to −13.7)	<.001	34	.18

Abbreviations: BPD, bronchopulmonary dysplasia; %FEV_1_, percentage predicted forced expiratory volume in 1 second; NA, not applicable.

## Discussion

The current systematic review extends the previous review^1^ by including a larger body of evidence relevant to the contemporary neonatal era and addresses several questions that were not previously possible to address. The preterm group had deficits of %FEV_1_ of −9.2% while those without BPD had deficits of −5.8% and those with BPD had deficits of approximately −16% regardless of whether they had BPD28 or BPD36. Over the last 3 decades, %FEV_1_ values have improved; we speculate that part of these improvements followed the introduction of surfactant in the early 1990s. There did not appear to be a change in %FEV_1_ in childhood or in young adulthood. Further, we noted that when compared with data from the Scandinavian countries, %FEV_1_ decrements were greater in studies from North America and western Europe, which have similar levels of economic development.

Overall, in the preterm group, %FEV_1_ was decreased by −9.2%, from which −5.8% can possibly be attributed to preterm-born participants without BPD and approximately −16% to the BPD group, regardless of the definition used to report BPD. For the preterm-born group without BPD, there was no change over time, measured by year of birth. This is to be expected because the majority of infants born at more than 32 weeks’ gestation do not require such medical interventions as mechanical ventilation and supplemental oxygen. Thus, the degree of difference between the term and preterm populations is likely to remain static over time as improvements in health due to socioeconomic factors, nutrition, antenatal medical care, etc, will benefit both populations, with the relative difference likely to remain largely unchanged. In contrast, medical interventions are likely to be common in the BPD group with the prevailing interventions likely to affect lung function outcomes. Regardless of definition of BPD, %FEV_1_ appears to have improved significantly over time (Figure 2). One factor that may explain this improvement is the introduction of exogeneous surfactant treatment in the early 1990s. Therefore, we compared those born before or in the year 1990 with those born in and after 1993, assuming most infants born thereafter will be treated with surfactant initially as rescue therapy and then prophylactically in more recent cohorts. In addition to surfactant treatment, which increased gradually throughout the 1990s, other contributions to improvements in neonatal care include the routine use of antenatal maternal corticosteroid administration from the mid 1990s, improved nutrition, gentler ventilation techniques, and permissive hypercarbia; decreased maternal smoking due to antismoking legislature will also have contributed to these improvements.

It is interesting to note that the deficits in %FEV_1_ were similar between the BPD28 and BPD36 groups. The former group will have included participants with both mild and moderate/severe BPD while the latter will have included only those with moderate/severe BPD. Because we were unable to separate the severity of BPD in the majority of publications, we were not able to separately report on participants with mild or moderate/severe BPD. Increasingly, it is recognized that BPD is a poor predictor of low lung function in childhood, and it has also recently been reported that gestation and intrauterine growth restriction are better associated with low lung function in childhood than BPD in fully adjusted models.^62^

For all the groups we studied, there did not appear to be any change in %FEV_1_ with increasing age, in childhood or young adulthood, except for the all BPD and BPD28 populations. This latter observation can be explained by the increasing %FEV_1_ in the term-born control group over time rather than any changes in the preterm-born population (eFigure 5 in the Supplement). When this anomaly of increasing %FEV_1_ over time in the term population is taken into account, it appears that the preterm group with BPD28 probably does not change with increasing age. This is an important observation as there is concern that there may be some deterioration in lung function with increasing age,^103,104^ although several other longitudinal publications do not report any deterioration but support the concept of “lung tracking.”^26,105^ Nevertheless, adequately powered longitudinal studies of lung function for this population from infancy are necessary to delineate this observation further.

There is limited information on how regional differences in health care are associated with BPD outcomes, so we compared data from similarly economically developed geographical regions. For the preterm population without BPD, the findings for %FEV_1_ deficits when compared with the term population were largely similar between the regions when Scandinavia was used as the reference population. However, for the group of all participants with BPD, there were differences between the Scandinavian population and those in North America (−5.5%; 95% CI, −10.7 to −0.3; *P* = .04) and western Europe (−4.1%; 95% CI, −8.8 to 0.5; *P* = .08) but not those in Australasia (−3.9; 95% CI, −9.8 to 2.1; *P* = .20). We choose Scandinavia as the reference population because it has superior outcomes for several perinatal conditions, including stillbirth and neonatal mortality,^106^ when compared with populations from similarly economically developed countries. It is unknown whether these differences are due to genetic or environmental factors, but clearly understanding and investigating potential environmental factors, especially differences in clinical care of babies at risk of BPD, may reveal areas of global improvement.

As expected, publication bias was observed for the all preterm-born group, which was a disparate group. It was not observed in any of the other groups we studied. There was a range of quality scores with some studies having low scores. This can partially be explained by the lack of information available to enable us to fully score the quality in several articles.

### Strengths and Limitations

There are several strengths and weaknesses. We have identified the largest group of participants thus far studied, including 7094 preterm-born and 17 700 term-born participants. We were not able to include a significant number of studies because they did not include a term-born reference population. The deterioration in %FEV_1_ in the BPD28 group was likely due to increases in %FEV_1_ for the term-born reference population, clearly demonstrating why a local term-born population should be included to reflect local socioeconomic, health, and genetic factors. We were limited by the information reported; we especially wanted to report outcomes for late preterm infants but were unable to do so, representing a deviation from our original protocol. Data from 1 excellent study was included in the main analysis but not in the additional analyses (associations between year of birth, age of participants, surfactant usage, and region) because the participants studied were born well before BPD was described and the age of the population would have skewed the analyses.^61^ We focused only on FEV_1_ as it is more reliable and reproducible^107^ than the other spirometry measures. Importantly, it is associated not only with lung health but also with cardiovascular outcomes and all-cause mortality.^108,109^

## Conclusions

This comprehensive systematic review, which has collated %FEV_1_ data from a large number of preterm- and term-born participants, noted deficits in %FEV_1_ for preterm-born participants when compared with term-born participants. Decrements were larger for the preterm-born participants who had BPD. Improvements in %FEV_1_ were noted over time, especially for the BPD groups, possibly due to the introduction of surfactant and improvement in early-life therapies. Differences were also observed for %FEV_1_ when geographical areas were compared with Scandinavian countries. These results emphasize the importance of being aware of the potential deficits^11^ when treating preterm-born survivors and of finding suitable treatments for the deficits observed in %FEV_1_.^102^

## Notes

Supplement. **eFigure 1.** Study selection flow diagram **eFigure 2.** Percentage predicted forced expiratory volume in 1 s (%FEV_1_) for all the preterm-born subjects compared with term control group **eFigure 3.** Percentage predicted forced expiratory volume in 1 s (%FEV_1_) for all the preterm-born subjects without BPD compared with term control group. **eFigure 4.** Percentage predicted forced expiratory volume in 1 s (%FEV_1_) for all the preterm-born subjects with BPD compared with term control group **eTable 1.** %FEV_1_ before (≤1990) and after surfactant (≥1993) for preterm-born groups compared to term groups **eTable 2.** %FEV_1_ by continent for whole preterm-born population compared to term control group **eTable 3.** Difference in %FEV_1_ between Prem-born and Term groups modelled on Continents: Western Europe v Scandinavia **eFigure 5.** %FEV_1_ BPD_28_ Age **eFigure 6.** Publication Bias **eAppendix 1.** The effect of premature birth compared to term birth on later lung function: a systematic review of the literature **eAppendix 2.** Assessment of study quality **eTable 4.** Description of the included articles **eTable 5.** Demographics of the included articles **eTable 6.** Lung function outcomes of the included articles
eReferences
