# Supplementary material for: Geographical Differences and Temporal Improvements in Forced Expiratory Volume in 1 Second of Preterm-Born Children: A Systematic Review and Meta-analysis
Source: JAMA Pediatr. 2022 Jun 27;176(9):867–77. doi: 10.1001/jamapediatrics.2022.1990 (PMC9237805; doi:10.1001/jamapediatrics.2022.1990)
Supplement: Supplement. — eFigure 1. Study selection flow diagram eFigure 2. Percentage predicted forced expiratory volume in 1 s (%FEV1) for all the preterm-born subjects compared with term control group eFigure 3. Percentage predicted forced expiratory volume in 1 s (%FEV1) for all the preterm-born subjects without BPD compared with term control group. eFigure 4. Percentage predicted forced expiratory volume in 1 s (%FEV1) for all the preterm-born subjects with BPD compared with term control group eTable 1. %FEV1 before (≤1990) and after surfactant (≥1993) for preterm-born groups compared to term groups eTable 2. %FEV1 by continent for whole preterm-born population compared to term control group eTable 3. Difference in %FEV1 between Prem-born and Term groups modelled on Continents: Western Europe v Scandinavia eFigure 5. %FEV1 BPD28 Age eFigure 6. Publication Bias eAppendix 1. The effect of premature birth compared to term birth on later lung function: a systematic review of the literature eAppendix 2. Assessment of study quality eTable 4. Description of the included articles eTable 5. Demographics of the included articles eTable 6. Lung function outcomes of the included articles eReferences [file jamapediatr-e221990-s001.pdf]

Supplemental Online Content

Kotecha SJ, Gibbons JTD, Course CW, et al. Geographical differences and temporal improvements in forced expiratory volume in 1 second of preterm-born children: a systematic review and meta-analysis. *JAMA Pediatr*. Published online June 27, 2022. doi:10.1001/jamapediatrics.2022.1990

eFigure 1. Study selection flow diagram

eFigure 2. Percentage predicted forced expiratory volume in 1 s (%FEV_1) for all the preterm-born subjects compared with term control group

eFigure 3. Percentage predicted forced expiratory volume in 1 s (%FEV_1) for all the preterm-born subjects without BPD compared with term control group.

eFigure 4. Percentage predicted forced expiratory volume in 1 s (%FEV_1) for all the preterm-born subjects with BPD compared with term control group

eTable 1. %FEV_1 before (≤1990) and after surfactant (≥1993) for preterm-born groups compared to term groups

eTable 2. %FEV_1 by continent for whole preterm-born population compared to term control group

eTable 3. Difference in %FEV_1 between Prem-born and Term groups modelled on Continents: Western Europe v Scandinavia

eFigure 5. %FEV_1 BPD_{28} Age

eFigure 6. Publication Bias

eAppendix 1. The effect of premature birth compared to term birth on later lung function: a systematic review of the literature

eAppendix 2. Assessment of study quality

eTable 4. Description of the included articles

eTable 5. Demographics of the included articles

eTable 6. Lung function outcomes of the included articles

eReferences

This supplemental material has been provided by the authors to give readers additional information about their work.

© 2022 Kotecha SJ et al. *JAMA Pediatrics.*

eFigure 1. Study selection flow diagram

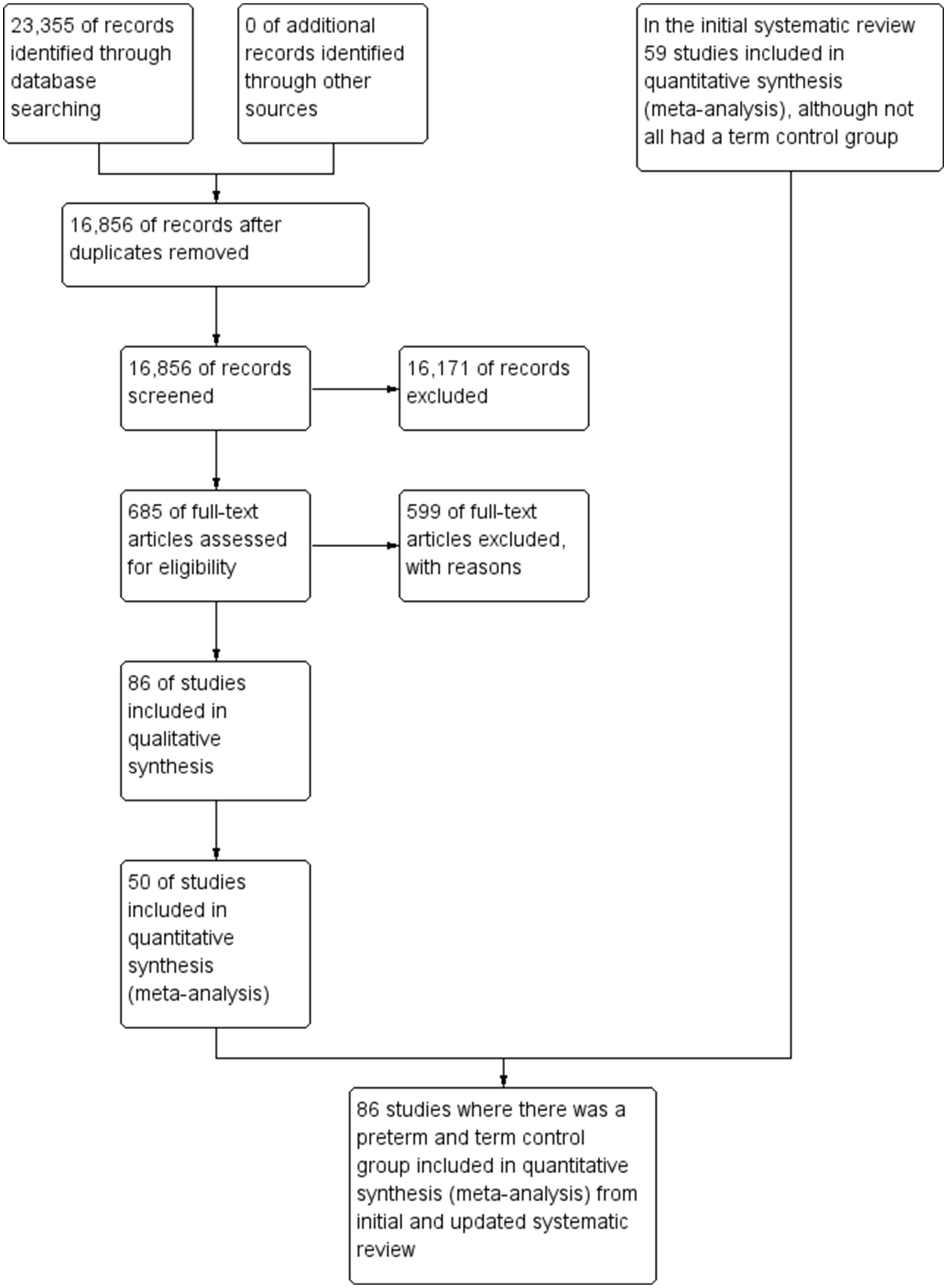

© 2022 Kotecha SJ et al. *JAMA Pediatrics.*

eFigure 2. Percentage predicted forced expiratory volume in 1 s (%FEV$_1$) for all the preterm-born subjects compared with term control group.

Study or Subgroup	Preterm Mean	Preterm SD	Preterm Total	term Mean	term SD	term Total	Weight	Mean Difference IV, Random, 95% CI
Abreu 2007	99.4348	12.6089	23	102	15	17	0.8%	-2.57 [-11.36, 6.23]
Anand 2003	94.9	13.8	128	96.5	10.8	128	1.4%	-1.60 [-4.64, 1.44]
Arigliani 2020	95.25	13.32	47	103.11	9.8	60	1.3%	-7.86 [-12.40, -3.32]
Bader 1987	73	19	10	93	11.3	8	0.5%	-20.00 [-34.14, -5.86]
Baraldi 2005	84.1	15.5	62	100.1	12.8	31	1.1%	-16.00 [-21.93, -10.07]
Barker 2003	95.5	15.282	26	106	11	13	0.9%	-10.50 [-18.88, -2.12]
Bertrand 1985	76	13.7356	22	84.5806	10.2038	31	1.0%	-8.58 [-15.35, -1.81]
Bozzetto 2016	76.6	16.5	27	108.2	10.6	27	1.0%	-31.60 [-39.00, -24.20]
Bui 2022	97.5	15.2	218	98.8	29.3	1227	1.4%	-1.30 [-3.90, 1.30]
Burns 2008	88.98	13.47	54	97.73	10.89	55	1.3%	-8.75 [-13.35, -4.15]
Cazzato 2012	86.8	13.32	48	102.17	10.51	46	1.2%	-15.37 [-20.21, -10.53]
Chang 2020	91.49	13.2	85	100.53	13.91	29	1.1%	-9.04 [-14.83, -3.25]
De Kleine 1990	87.83	17.33	76	95	12	39	1.2%	-7.17 [-12.59, -1.75]
Devakumar 2015	85.74	9.92	48	86.92	9.33	697	1.4%	-1.18 [-4.07, 1.71]
Doyle 2001	94.82	14.42	169	104.6	13.2	39	1.2%	-9.78 [-14.46, -5.10]
Doyle 2006	84.9	12.7	240	97.9	11.8	208	1.4%	-13.00 [-15.27, -10.73]
Durlak 2021	93.57	14.93	28	100.76	10.04	30	1.1%	-7.19 [-13.78, -0.60]
Evensen 2009	85.2	10.949	37	98.1	11.1122	63	1.3%	-12.90 [-17.37, -8.43]
Fawke 2010	83	14	182	100	12	161	1.4%	-17.00 [-19.75, -14.25]
Filippo 2021	105.93	15.32	55	108.28	10.62	55	1.2%	-2.35 [-7.28, 2.58]
Flahault 2019	90.32	12.03	101	99.83	10.51	105	1.4%	-9.51 [-12.60, -6.42]
Fortuna 2016	88.95	12.4629	48	105.8	9.6	27	1.2%	-16.85 [-21.90, -11.80]
Gaffin 2020	98.5	17.3	47	102.1	18.5	250	1.2%	-3.60 [-9.05, 1.85]
Galdes-Sebaldt 1989	82.37	7.89	30	92	5.2	27	1.4%	-9.63 [-13.07, -6.19]
Giacoia 1997	79.3	22.0634	24	97.2	15.9349	12	0.6%	-17.90 [-30.52, -5.28]
Gough 2014	88.1775	17.245	96	101.16	11.4	55	1.3%	-12.98 [-17.56, -8.40]
Gross 1998	91.3	19	96	97	12	108	1.3%	-5.70 [-10.12, -1.28]
Hacking 2013	90.9	15.4	150	102.5	15.4	149	1.4%	-11.60 [-15.09, -8.11]
Hadchouel 2018	93.02	15.32	274	98.89	11.8	44	1.3%	-5.87 [-9.80, -1.94]
Hakulinen 1996	87.7661	15.9719	31	101.25	8.3	20	1.0%	-13.48 [-20.18, -6.79]
Hamon 2012	102.17	12.38	42	108.04	9.57	27	1.2%	-5.87 [-11.07, -0.67]
Hart 2021	91.16	12.46	544	95.7	9.2	195	1.5%	-4.54 [-6.20, -2.88]
Jacob 1998 + 1997	74.35	19.512	30	94.3	8.3	13	0.9%	-19.95 [-28.26, -11.64]
Joshi 2013	87.28	14.26	62	97.5	11.78	30	1.2%	-10.22 [-15.73, -4.71]
Karila 2009	79.1	19.3	20	106.3	11.3	18	0.8%	-27.20 [-37.14, -17.26]
Karnaushkina 2016	79.4	2.7	10	91.2	4.6	9	1.4%	-11.80 [-15.24, -8.36]
Kennedy 2000	91	14.9	102	102.1	10.2	82	1.3%	-11.10 [-14.74, -7.46]
Kilbride 2003	85	14	50	91	9	25	1.2%	-6.00 [-11.24, -0.76]
Kilbride 2012	100.6	16.2398	30	107	13	9	0.7%	-6.40 [-16.69, 3.89]
Konefal 2010	95.07	17.54	31	96.2	20.2	19	0.7%	-1.13 [-12.11, 9.85]
Konefal 2013	89	20.24	58	98.3	14.04	90	1.1%	-9.30 [-15.26, -3.34]
Korhonen 2004	92.5833	14.1077	60	99	11	33	1.2%	-6.42 [-11.60, -1.24]
Kotecha 2012	97.9878	11.4576	382	100.19	11.8	6144	1.5%	-2.20 [-3.39, -1.02]
Kwinta 2013	81.3	13	22	95.8	8	20	1.1%	-14.50 [-20.97, -8.03]
Landry 2015	89.0682	16.2154	88	98	9	35	1.3%	-8.93 [-13.44, -4.42]
MacLean 2016	91.87	14.62	100	101.12	11.91	64	1.3%	-9.25 [-13.34, -5.16]
Mai 2003	92	12	74	95	10	64	1.3%	-3.00 [-6.67, 0.67]
Mieskonen 2002	84.1	14.3	40	101.7	8.4	14	1.1%	-17.60 [-23.84, -11.36]
Mitchell 1998	81.5	18.1209	20	91	10	14	0.8%	-9.50 [-19.01, 0.01]
Molgat-Seon 2019	86.3182	5.9758	44	103	2	20	1.5%	-16.68 [-18.65, -14.71]
Morata-Alba 2019	90.2	13.9	116	96	13	116	1.4%	-5.80 [-9.26, -2.34]
Morsing 2011	90.55	12.56	62	98.3	14.26	31	1.1%	-7.75 [-13.66, -1.84]
Narayanan 2013	98.0972	12.7212	58	101.82	10.74	61	1.3%	-3.72 [-7.96, 0.52]
Nasanen-Gilmore 2018	97.4632	12.0337	378	100.29	10.97	341	1.5%	-2.83 [-4.51, -1.15]
Nordlund 2016	76.9	11.6	30	84.1	10.9	30	1.1%	-7.20 [-12.90, -1.50]
Northway 1990	74.8	14.5	25	100.4	10.9	53	1.1%	-25.60 [-32.00, -19.20]
Odberg 2010	106.8	13.5	134	110.2	14.2	135	1.4%	-3.40 [-6.71, -0.09]
Palta 2007	86	14	265	97	12	360	1.5%	-11.00 [-13.09, -8.91]
Panagiotounakou 2019	91	10.1	85	92.6	12.38	62	1.3%	-1.60 [-5.36, 2.16]
Pianosi 2000	84.5938	13.4089	32	90	8	15	1.1%	-5.41 [-11.57, 0.76]
Praprotnik 2015	83.8446	13.061	56	91.1	9.5	33	1.2%	-7.26 [-11.97, -2.54]
Prenzel 2020	91.26	16.26	39	106.16	10.74	40	1.1%	-14.90 [-20.99, -8.81]
Ronkainen 2015	86.4	11.8	88	94.9	10.1	88	1.4%	-8.50 [-11.75, -5.25]
Ruf 2019	91.2727	17.4115	22	105	8	15	0.9%	-13.73 [-22.05, -5.40]
Santuz 1995	83	13	12	100	8	16	0.9%	-17.00 [-25.33, -8.67]
Siltanen 2004	92	13.1	50	104	8	54	1.3%	-12.00 [-16.21, -7.79]
Simpson 2017	91.61	13.32	131	100.53	10.62	48	1.3%	-8.92 [-12.69, -5.15]
Smith 2008	85	12.4	123	95	10.2	34	1.3%	-10.00 [-14.07, -5.93]
Sorensen 2018	90.504	14.3854	70	102.76	12.38	38	1.2%	-12.26 [-17.44, -7.07]
Teig 2012	92.83	9.75	16	109.1	26.04	11	0.4%	-16.27 [-32.38, -0.16]
Thunqvist 2016	95.94	11.94	106	99.94	10.67	1686	1.4%	-4.00 [-6.33, -1.67]
Thunqvist 2017	91.61	11.8	90	104.87	11.8	98	1.4%	-13.26 [-16.64, -9.88]
Tukova 2019	92.99	12.26	48	98.37	14.03	526	1.3%	-5.38 [-9.05, -1.71]
Turner 2013	96.98	13.25	37	98.28	13.98	563	1.3%	-1.30 [-5.72, 3.12]
Um-Bergstrom 2019	96.7	15.2402	49	107.57	11.35	24	1.1%	-10.87 [-17.10, -4.64]
Vardar-Yagli 2015	78	21.2	18	98	11.7	20	0.7%	-20.00 [-31.05, -8.95]
Vollsaeter 2013b	87.3602	39.3854	48	102.64	15.08	46	0.6%	-15.28 [-27.24, -3.32]
Vollsaeter 2013c	90.0723	9.4128	35	99.43	8.74	35	1.3%	-9.36 [-13.61, -5.10]
Vollsaeter 2015	92.43	10.68	57	96.42	11.67	54	1.3%	-3.99 [-8.16, 0.18]
Von Mutius 1993	98.6966	11.401	118	100.4	9.1935	2113	1.5%	-1.70 [-3.80, 0.39]
Vrijlandt 2006	95.4	15.9	42	109.6	13.4	48	1.1%	-14.20 [-20.32, -8.08]
Vrijlandt 2018	92	12	37	97	10	34	1.2%	-5.00 [-10.12, 0.12]
Wheeler 1984	95.44	21.7036	25	104	16	11	0.6%	-8.56 [-21.28, 4.16]
Winck 2016	104.75	19.07	48	108.39	13.2	45	1.0%	-3.64 [-10.27, 2.99]
Yaacoby-Bianu 2019	97.63	14.24	29	103.27	9.74	30	1.1%	-5.64 [-11.89, 0.61]
Yang 2020	92.2	14.14	224	98.53	13.79	100	1.4%	-6.33 [-9.61, -3.05]
**Total (95% CI)**			**7094**			**17700**	**100.0%**	**-9.18 [-10.40, -7.96]**

Heterogeneity: Tau² = 25.39; Chi² = 655.84, df = 85 (P < 0.00001); I² = 87%
Test for overall effect: Z = 14.76 (P < 0.00001)

Mean Difference IV, Random, 95% CI

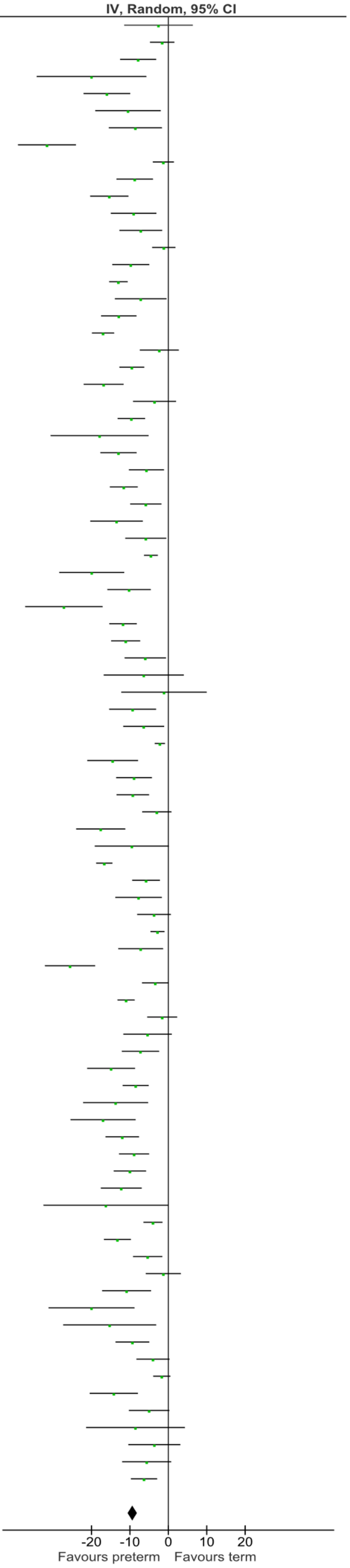

© 2022 Kotecha SJ et al. *JAMA Pediatrics.*

eFigure 3. Percentage predicted forced expiratory volume in 1 s (%FEV_1) for all the preterm-born subjects without BPD compared with term control group.

Study or Subgroup	Preterm no BPD Mean	SD	Total	Term Mean	SD	Total	Weight	Mean Difference IV, Random, 95% CI
Abreu 2007	100	14	10	102	15	17	1.0%	-2.00 [-13.23, 9.23]
Arigliani 2020	100.29	12.38	30	103.11	9.8	60	2.5%	-2.82 [-7.90, 2.26]
Baraldi 2005	90.3	15.6	31	100.1	12.8	31	1.8%	-9.80 [-16.90, -2.70]
Barker 2003	101	15	13	106	11	13	1.2%	-5.00 [-15.11, 5.11]
Cazzato 2012	89.15	15.67	26	102.17	10.51	46	1.9%	-13.02 [-19.77, -6.27]
Chang 2020	96.42	12.97	30	100.53	13.91	29	1.9%	-4.11 [-10.98, 2.76]
De Kleine 1990	90.34	16.2	65	95	12	39	2.4%	-4.66 [-10.11, 0.79]
Doyle 2001	96.7	12.6	130	104.6	13.2	39	2.7%	-7.90 [-12.57, -3.23]
Doyle 2006	87.1	11.5	151	97.9	11.8	208	3.6%	-10.80 [-13.24, -8.36]
Durlak 2021	95.48	15.08	22	100.76	10.04	30	1.8%	-5.28 [-12.53, 1.97]
Fawke 2010	90	15	53	100	12	161	2.8%	-10.00 [-14.44, -5.56]
Filippo 2021	107.1	15.32	50	108.28	10.62	55	2.5%	-1.18 [-6.27, 3.91]
Fortuna 2016	94.2	9.9	20	105.8	9.6	27	2.3%	-11.60 [-17.25, -5.95]
Giacoia 1997	85.9	21.8238	12	97.2	15.9349	12	0.6%	-11.30 [-26.59, 3.99]
Gough 2014	96.98	15.22	40	101.16	11.4	55	2.3%	-4.18 [-9.78, 1.42]
Gross 1998	98	18	53	97	12	108	2.4%	1.00 [-4.35, 6.35]
Hacking 2013	95.01	12.85	94	102.5	15.4	149	3.2%	-7.49 [-11.08, -3.90]
Hadchouel 2018	95.37	14.14	225	98.89	11.8	44	3.0%	-3.52 [-7.47, 0.43]
Hakulinen 1996	88.25	22.91	11	101.25	8.3	20	0.7%	-13.00 [-27.02, 1.02]
Jacob 1998 + 1997	85.1	10.8	15	94.3	8.3	13	1.8%	-9.20 [-16.29, -2.11]
Joshi 2013	92	14.1	33	97.5	11.78	30	2.1%	-5.50 [-11.90, 0.90]
Kennedy 2000	95.4	11.4	76	102.1	10.2	82	3.2%	-6.70 [-10.08, -3.32]
Kilbride 2003	89	13	34	85	14	50	2.2%	4.00 [-1.84, 9.84]
Korhonen 2004	95	14	31	99	11	33	2.1%	-4.00 [-10.19, 2.19]
Landry 2015	94	12.8313	57	98	9	35	2.8%	-4.00 [-8.47, 0.47]
MacLean 2016	94.19	12.97	53	101.12	11.91	64	2.7%	-6.93 [-11.48, -2.38]
Mieskonen 2002	89.8	13	18	101.7	8.4	14	1.7%	-11.90 [-19.34, -4.46]
Mitchell 1998	85	15	10	91	14	40	1.1%	-6.00 [-16.26, 4.26]
Molgat-Seon 2019	92	2	19	103	2	20	4.0%	-11.00 [-12.26, -9.74]
Narayanan 2013	95.72	14.5	19	101.82	10.74	61	1.9%	-6.10 [-13.15, 0.95]
Palta 2007	88	14	206	97	12	360	3.7%	-9.00 [-11.28, -6.72]
Panagiotounakou 2019	92.2	10.9	43	92.6	12.38	62	2.8%	-0.40 [-4.88, 4.08]
Pianosi 2000	83	13	15	90	8	15	1.7%	-7.00 [-14.72, 0.72]
Praprotnik 2015	89.8	11.2	33	91.1	9.5	33	2.6%	-1.30 [-6.31, 3.71]
Ronkainen 2015	89.4	11.1	39	94.9	10.1	88	2.9%	-5.50 [-9.57, -1.43]
Ruf 2019	97	11	13	105	8	15	1.8%	-8.00 [-15.22, -0.78]
Simpson 2017	97.6	11.68	52	100.53	10.62	48	2.8%	-2.93 [-7.30, 1.44]
Sorensen 2018	95.01	14.14	28	102.76	12.38	38	2.0%	-7.75 [-14.30, -1.20]
Um-Bergstrom 2019	104.5	12.96	23	107.57	11.35	24	1.9%	-3.07 [-10.05, 3.91]
Vollsaeter 2013b	95.25	13.86	11	102.64	15.08	46	1.3%	-7.39 [-16.67, 1.89]
Vollsaeter 2013c	97.13	7.54	9	99.43	8.74	35	2.3%	-2.30 [-8.01, 3.41]
Vollsaeter 2015	93.49	10.23	26	96.42	11.67	54	2.6%	-2.93 [-7.95, 2.09]
Vrijlandt 2006	99.2	17.9	12	109.6	13.4	48	1.1%	-10.40 [-21.21, 0.41]
Wheeler 1984	106	17	14	104	16	11	0.8%	2.00 [-10.99, 14.99]
Yang 2020	94.19	12.73	178	98.53	13.79	100	3.3%	-4.34 [-7.63, -1.05]
**Total (95% CI)**			**2133**			**2562**	**100.0%**	**-5.81 [-7.10, -4.52]**

Heterogeneity: Tau² = 10.33; Chi² = 128.58, df = 44 (P < 0.00001); I² = 66%
Test for overall effect: Z = 8.84 (P < 0.00001)

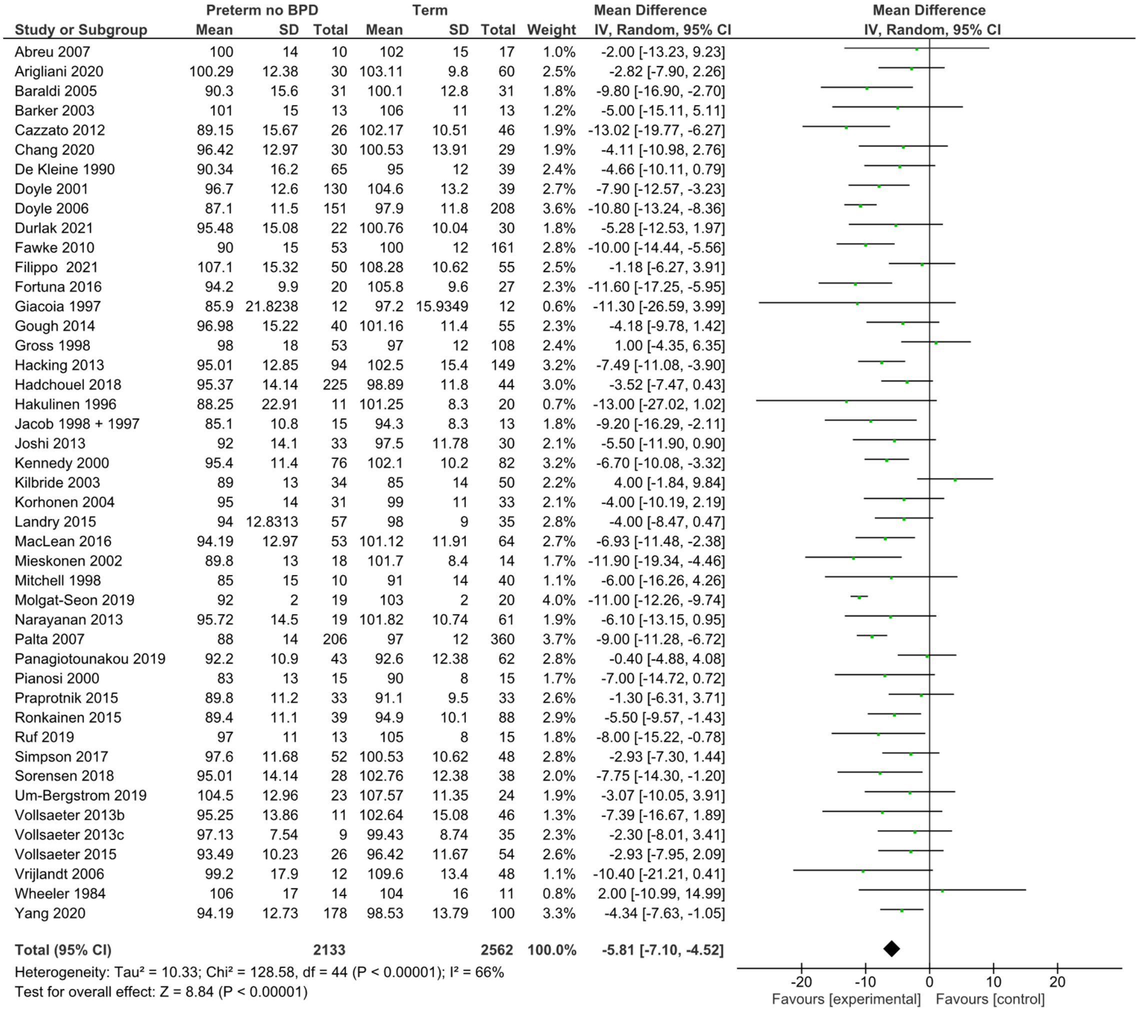

© 2022 Kotecha SJ et al. *JAMA Pediatrics.*

eFigure 4. Percentage predicted forced expiratory volume in 1 s (%FEV_1) for all the preterm-born subjects with BPD compared with term control group.

	BPD			term				Mean Difference
Study or Subgroup	Mean	SD	Total	Mean	SD	Total	Weight	IV, Random, 95% CI
Abreu 2007	99	12	13	102	15	17	1.4%	-3.00 [-12.66, 6.66]
Ahrens 1991	77.11	12.42	19	78	20	9	0.9%	-0.89 [-15.10, 13.32]
Arigliani 2020	86.21	10.04	17	103.11	9.8	60	2.2%	-16.90 [-22.28, -11.52]
Bader 1987	73	19	10	93	11.3	8	0.9%	-20.00 [-34.14, -5.86]
Baraldi 2005	77.8	12.8	31	100.1	12.8	31	2.0%	-22.30 [-28.67, -15.93]
Barker 2003	90	14	13	106	11	13	1.4%	-16.00 [-25.68, -6.32]
Bozzetto 2016	76.6	16.5	27	108.2	10.6	27	1.8%	-31.60 [-39.00, -24.20]
Cazzato 2012	83.98	9.1	22	102.17	10.51	46	2.3%	-18.19 [-23.06, -13.32]
Chang 2020	88.79	12.73	55	100.53	13.91	29	2.0%	-11.74 [-17.82, -5.66]
De Kleine 1990	73	17	11	95	12	39	1.3%	-22.00 [-32.73, -11.27]
Doyle 2001	88.5	18.2	39	104.6	13.2	39	1.9%	-16.10 [-23.16, -9.04]
Doyle 2006	81.1	13.7	89	97.9	11.8	208	2.5%	-16.80 [-20.07, -13.53]
Durlak 2021	86.56	13.2	6	100.76	10.04	30	1.2%	-14.20 [-25.36, -3.04]
Fawke 2010	80	13	129	100	12	161	2.6%	-20.00 [-22.91, -17.09]
Filippo 2021	101.23	12.97	5	108.28	10.62	55	1.2%	-7.05 [-18.76, 4.66]
Fortuna 2016	85.2	12.9	28	105.8	9.6	27	2.1%	-20.60 [-26.60, -14.60]
Giacoia 1997	72.7	21.131	12	97.2	15.9349	12	0.9%	-24.50 [-39.47, -9.53]
Gough 2014	81.89	15.9	56	101.16	11.4	55	2.2%	-19.27 [-24.41, -14.13]
Gross 1998	83	17	43	97	12	108	2.1%	-14.00 [-19.56, -8.44]
Hacking 2013	86.09	14.73	56	102.5	15.4	149	2.3%	-16.41 [-20.99, -11.83]
Hadchouel 2018	83.63	14.14	49	98.89	11.8	44	2.2%	-15.26 [-20.54, -9.98]
Hakulinen 1996	87.5	11.24	20	101.25	8.3	20	2.0%	-13.75 [-19.87, -7.63]
Jacob 1998 + 1997	63.6	20.6	15	94.3	8.3	13	1.2%	-30.70 [-42.06, -19.34]
Joshi 2013	81.9	12.62	29	97.5	11.78	30	2.0%	-15.60 [-21.83, -9.37]
Karila 2009	79.1	19.3	20	106.3	11.3	18	1.4%	-27.20 [-37.14, -17.26]
Kennedy 2000	78.4	17	26	102.1	10.2	82	1.9%	-23.70 [-30.60, -16.80]
Kilbride 2003	72	15	16	91	9	25	1.7%	-19.00 [-27.15, -10.85]
Korhonen 2004	90	14	29	99	11	34	2.0%	-9.00 [-15.30, -2.70]
Landry 2015	80	18	31	98	9	35	1.9%	-18.00 [-25.00, -11.00]
MacLean 2016	89.26	16.02	47	101.12	11.91	64	2.2%	-11.86 [-17.29, -6.43]
Mieskonen 2002	73.5	12	9	101.7	8.4	14	1.5%	-28.20 [-37.19, -19.21]
Mitchell 1998	78	21	10	91	14	10	0.8%	-13.00 [-28.64, 2.64]
Molgat-Seon 2019	82	4	25	103	2	20	2.7%	-21.00 [-22.80, -19.20]
Narayanan 2013	94.07	11.56	18	101.82	10.74	61	2.1%	-7.75 [-13.73, -1.77]
Nordlund 2016	76.9	11.6	30	84.1	10.9	30	2.1%	-7.20 [-12.90, -1.50]
Northway 1990	74.8	14.5	25	100.4	10.9	53	2.0%	-25.60 [-32.00, -19.20]
Palta 2007	78	13	59	97	12	360	2.5%	-19.00 [-22.54, -15.46]
Panagiotounakou 2019	89.9	9.2	42	92.6	12.38	62	2.4%	-2.70 [-6.85, 1.45]
Pianosi 2000	86	14	17	90	8	15	1.7%	-4.00 [-11.79, 3.79]
Praprotnik 2015	75.3	10.7	23	91.1	9.5	33	2.2%	-15.80 [-21.24, -10.36]
Prenzel 2020	91.26	16.26	39	106.16	10.74	40	2.0%	-14.90 [-20.99, -8.81]
Ronkainen 2015	84.3	11.4	49	94.9	10.1	88	2.4%	-10.60 [-14.43, -6.77]
Ruf 2019	83	22	9	105	8	15	0.9%	-22.00 [-36.93, -7.07]
Santuz 1995	83	13	12	100	8	16	1.6%	-17.00 [-25.33, -8.67]
Simpson 2017	87.62	12.85	79	100.53	10.62	48	2.4%	-12.91 [-17.04, -8.78]
Sorensen 2018	87.5	13.91	42	102.76	12.38	38	2.1%	-15.26 [-21.02, -9.50]
Thunqvist 2017	91.28	14.39	82	104.87	11.8	98	2.4%	-13.59 [-17.48, -9.70]
Um-Bergstrom 2019	89.8	13.88	26	107.57	11.35	24	1.9%	-17.77 [-24.78, -10.76]
Vardar-Yagli 2015	78	21.2	18	98	11.7	20	1.2%	-20.00 [-31.05, -8.95]
Vollsaeter 2013b	85.0146	44.1266	37	102.64	15.08	46	0.9%	-17.63 [-32.50, -2.75]
Vollsaeter 2013c	87.6292	8.8411	26	99.43	8.74	35	2.3%	-11.80 [-16.27, -7.34]
Vollsaeter 2015	91.49	11.58	31	96.42	11.67	54	2.2%	-4.93 [-10.06, 0.20]
Vrijlandt 2006	90.1	19.8	8	109.6	13.4	48	0.9%	-19.50 [-33.73, -5.27]
Wheeler 1984	82	20	11	104	16	11	0.8%	-22.00 [-37.14, -6.86]
Yang 2020	84.33	16.61	46	98.53	13.79	100	2.1%	-14.20 [-19.71, -8.69]
**Total (95% CI)**			**1736**			**2827**	**100.0%**	**-15.89 [-17.55, -14.23]**

Heterogeneity: Tau² = 25.49; Chi² = 224.76, df = 54 (P < 0.00001); I² = 76%

Test for overall effect: Z = 18.78 (P < 0.00001)

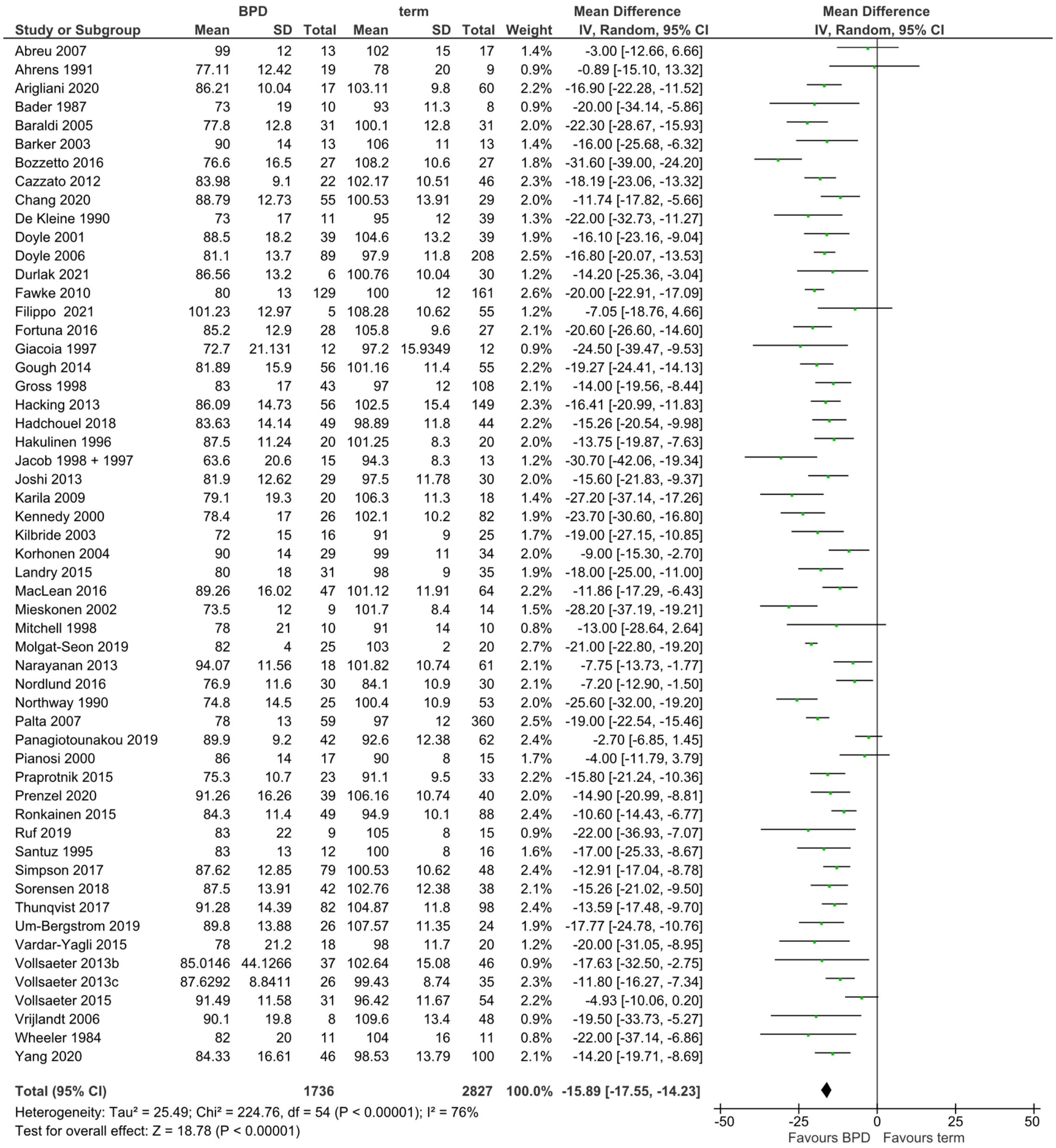

© 2022 Kotecha SJ et al. *JAMA Pediatrics.*

eTable 1. %FEV_1 before (≤1990) and after surfactant (≥1993) for preterm-born groups compared to term groups

	Number of studies	Number of preterm subjects	Number of term subjects	Mean Difference (95% CI)	P value	Heterogeneity		Number of studies	Number of preterm subjects	Number of term subjects	Effect Size OR (95% CI)	P value	Heterogeneity	
≤1990								**≥1993**						
All preterm compared to terms	30	2,397	1,958	-10.7% (-12.9, -8.4)	<0.00001	87%	<0.00001	40	3,239	5,639	-7.81% (-9.3, -6.3)	<0.00001	78%	<0.00001
preterm no BPD compared to terms	19	975	1,109	-6.3% (-8.4, -4.2)	<0.00001	71%	<0.00001	18	827	982	-5.5% (-7.0, -3.9)	<0.00001	36%	0.07
preterm BPD compared to terms	23	559	1,140	-18.3% (-20.5, -16.0)	<0.00001	57%	0.0005	22	879	1212	-13.3% (-15.7, -11.0)	<0.00001	75%	<0.00001
preterm BPD_{28} compared to terms	15	315	473	-17.5% (-20.4, -14.5)	<0.00001	61%	0.001	7	225	291	-11.4% (-15.9, -6.9)	<0.00001	78%	=0.0001
preterm BPD_{36} compared to terms	8	213	678	-19.4% (-23.1, -15.8)	<0.00001	52%	0.04	14	518	908	-14.6% (-17.1, -12.1)	<0.00001	64%	=0.0006

© 2022 Kotecha SJ et al. *JAMA Pediatrics.*

eTable 2. %FEV$_1$ by continent for whole preterm-born population compared to term control group

	Number of studies	Number of preterm subjects	Number of term subjects	Mean Difference OR (95% CI)	P value	Heterogeneity	
						I^2	P
Europe	52	4,383	13,751	-9.0% (-10.5, -7.4)	<0.00001	87%	<0.00001
Western Europe	29	2,771	10,217	-9.7% (-12.0, -7.3)	<0.00001	90%	<0.00001
Eastern Europe	6	243	718	-7.7% (-10.6, 4.9)	<0.00001	33%	0.19
Scandinavia	17	1,369	2,816	-8.3% (-10.6, -6.1)	<0.00001	81%	<0.00001
N America	18	1,039	1,160	-10.7% (-13.2, -8.2)	<0.00001	79%	<0.00001
S America	2	71	62	-3.3% (-8.6, 2.1)	0.23	0%	0.85
Asia	3	162	756	-4.8% (-9.8, 0.3)	0.06	69%	0.04
Australasia	8	1,055	582	-10.2% (-12.2, -8.2)	<0.00001	52%	0.04

© 2022 Kotecha SJ et al. *JAMA Pediatrics.*

eTable 3. Difference in $\%FEV_1$ between Prem-born and Term groups modelled on Continents – Western Europe v Scandinavia (ref)

Group	Continent	Number of Studies	Beta (95% CI)	p-value
**Prem no BPD**	Western Europe	40	-1.310 (-5.109, 2.489)	0.499
	North America		-0.539 (-4.517, 3.440)	0.791
	Australasia		-1.563 (-5.727, 2.602)	0.462
**Prem with BPD**	Western Europe	50	-4.144 (-8.776, 0.489)	0.080 ~
	North America		-5.535 (-10.734, -0.336)	0.037*
	Australasia		-3.866 (-9.803, 2.071)	0.202

~Near Sig p<0.1, * p<0.05, **p<0.001, ***p<0.0001

© 2022 Kotecha SJ et al. *JAMA Pediatrics.*

eFigure 5. %FEV_1 BPD_{28} Age

A) Preterm with BPD_{28}

Beta=-0.070
R^2=0.0%
p=0.777

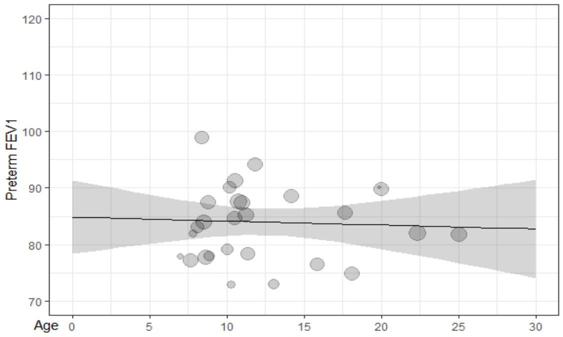

B) Term controls

Beta=0.501
R^2=11.2%
p=0.047

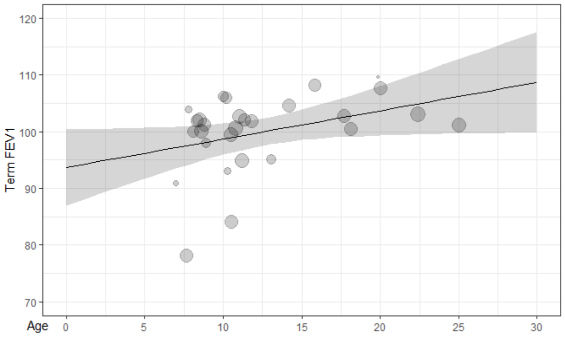

© 2022 Kotecha SJ et al. *JAMA Pediatrics.*

eFigure 6. Publication Bias

a) All Preterm

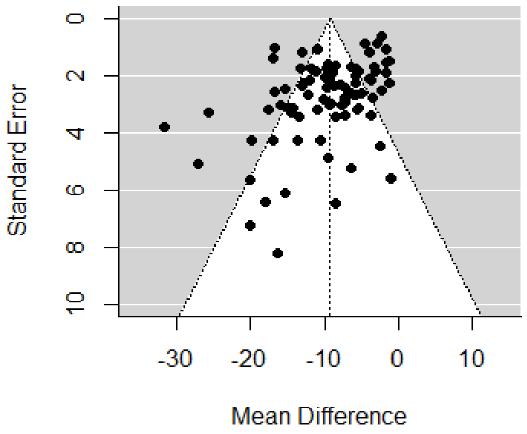

Rank Correlation Test for Funnel Plot Asymmetry Kendall's tau = -0.17, p = 0.020

b) Preterm subjects without BPD

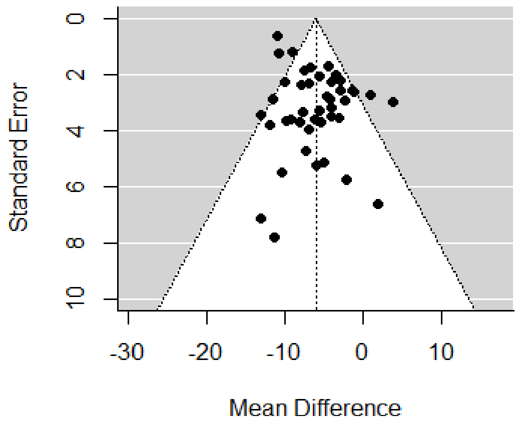

Rank Correlation Test for Funnel Plot Asymmetry Kendall's tau = -0.121, p = 0.255

c) Preterm subjects with BPD

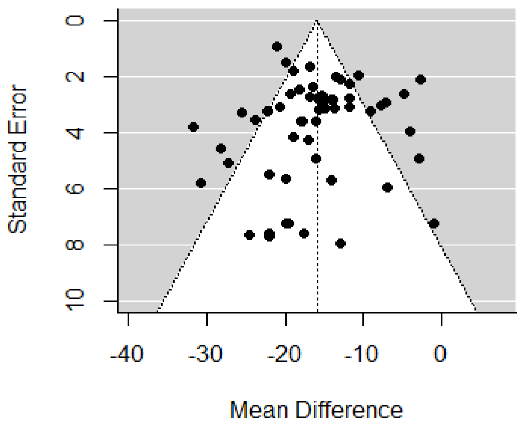

Rank Correlation Test for Funnel Plot Asymmetry Kendall's tau = -0.134, p = 0.151

d) Preterm subjects with BPD28

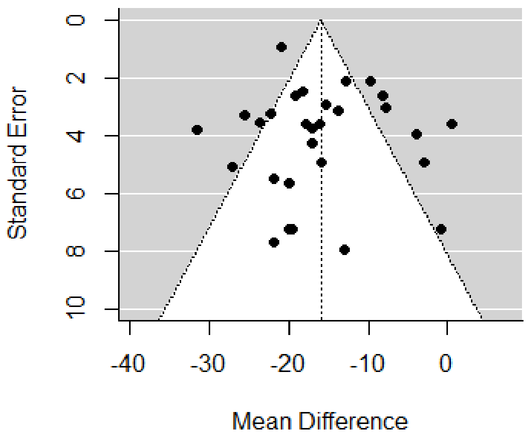

Rank Correlation Test for Funnel Plot Asymmetry Kendall's tau = -0.074, p = 0.59

e) Preterm subjects with BPD36

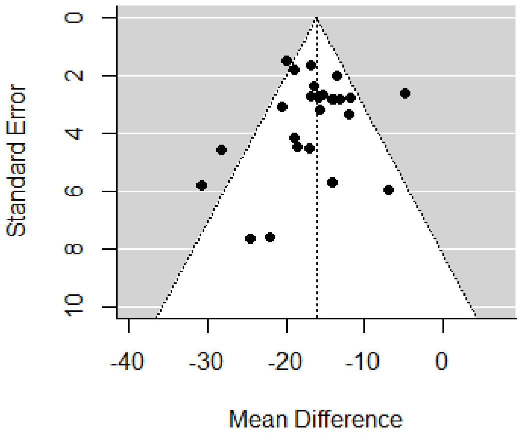

Rank Correlation Test for Funnel Plot Asymmetry Kendall's tau = -0.089, p = 0.541

© 2022 Kotecha SJ et al. *JAMA Pediatrics.*

eAppendix 1

The effect of premature birth compared to term birth on later lung function - a systematic review of the literature (UPDATED 2020)

Review Question

The aim is to extend and update the systematic review from 2011 (Ref 1) to update the overall observations and to address the questions that were not possible to address on the last occasion.

1. Are there deficits in lung function in later life in preterm babies compared to babies born at term?
    - Preterm<37 weeks' gestation and those with CLD (defined as supplemental oxygen dependency at 28 days of age and 36 weeks corrected age) compared to term controls
    - Was there change over time against year of birth?
    - Were there differences between males and females?
    - Were there differences between the pre-surfactant and post-surfactant eras?
    - Were there differences within the different gestation groups (<=28 weeks; 29-32 weeks and 33-36 weeks' gestation) against term born children?
2. How does lung function change longitudinally in preterm born compared to term babies?
    - Preterm group overall will be assessed
    - Those with CLD at 28 days and 36 weeks will be looked at separately
    - Were there differences between the pre-surfactant and post-surfactant eras?

Search strategy

A search strategy was developed for electronic databases using the keywords and MeSH headings below. The search strategy was used in the systematic review which was ran in 2011 till 2020. The Observational Studies search filter used by SIGN (Scottish Intercollegiate Guidelines Network) http://www.sign.ac.uk/methodology/filters.html#obs was adapted to retrieve types of study designs included in the review in 2020.

The search strategy will be modified to search rest of the bibliographic databases. In addition, a range of 'snowballing' techniques will be used to increase the sensitivity of the search, including reference list follow up, contact with subject experts and relevant websites/organisations, and table of content scanning for the top three most frequently cited journals.

Keywords/ MeSH headings

Bronchospirometry/
Bronchospirometries
Chronic respiratory questionnaire
FEF 25 75 Percent
Forced Expiratory Volume/
FEV1

© 2022 Kotecha SJ et al. *JAMA Pediatrics.*

Flow Rate, Maximal Expiratory/
Forced Expiratory Flow Rates/
Forced Vital Capacity
ISAAC questionnaire
Lung function test
Lung Volume Measurements/
MEFR

Maximal Midexpiratory Flow Rate/
Maximal Expiratory Flow Rate/
MMFR

Pulmonary Function Test

Respiratory Function Tests/

SGRQ
Spirometry/

Spirometries
St George's respiratory questionnaire
Timed Vital Capacity
Vital Capacity/
Volumes, Forced

Volume, Forced Expiratory/

Birth Weight/

Birth Weight Low/

Fetal Growth Retardation/

Infant, Low Birth Weight/
Infant, Very Low Birth Weight/
Infant, Extremely Low Birth Weight/
Infant, Premature/

IUGR

Intrauterine growth restriction
Low birth weight
Obstetric Labor, Premature/
Premature infant
Preterm labor
Premature labor
Premature birth
Preterm birth

Asthma/
bronchial asthma

Bronchopulmonary Dysplasia/

Chronic lung disease of infancy/

© 2022 Kotecha SJ et al. *JAMA Pediatrics.*

Hyaline Membrane Disease/
Chronic lung disease of prematurity
Respiratory Distress Syndrome, Newborn/
Pulmonary Disease, Chronic Obstructive/

Ovid MEDLINE - Search Strategy

1. exp Bronchospirometry/
2. exp Vital Capacity/
3. exp Forced Expiratory Volume/
4. Respiratory Function Tests/
5. exp Forced Expiratory Flow Rates/
6. exp Maximal Expiratory Flow Rate/
7. exp Maximal Midexpiratory Flow Rate/
8. Spirometry/
9. Lung Volume Measurements/
10. Bronchospirometries.mp.
11. Forced Vital Capacit*.mp.
12. Timed Vital Capacit*.mp.
13. (MEFR or MMFR or FEVt or SGRQ).mp.
14. St George's respiratory questionnaire.mp.
15. Chronic Respiratory Questionnaire.mp.
16. ISAAC questionnaire.mp.
17. Flow Rate, Maximal Expiratory/
18. Volume, Forced Expiratory/
19. Volumes, Forced/
20. Forced volume.mp.
21. ((Maximal or flow rate) adj2 expirator*).mp.
22. FEF 25 75 Percent.mp.
23. ((lung* or respiratory or pulmonary) adj2 (function* or expirator* capacit*)).mp.
24. spirometries.mp.
25. or/1-24
26. ((lung*1 or respiratory or pulmonary) adj2 (disease*1 or disorder*1)).mp.
27. exp Asthma/

© 2022 Kotecha SJ et al. *JAMA Pediatrics.*

28. exp Respiratory Distress Syndrome, Newborn/

29. Bronchial asthma.mp.

30. Chronic lung disease of prematurity.mp.

31. Chronic lung disease of infancy.mp.

32. Hyaline Membrane Disease/

33. Bronchopulmonary Dysplasia/

34. Pulmonary Disease, Chronic Obstructive/

35. or/26-34

36. exp Infant, Low Birth Weight/

37. exp Infant, Very Low Birth Weight/

38. exp Infant, Extremely Low Birth Weight/

39. exp Infant, Premature/

40. Obstetric Labor, Premature/

41. exp Premature Birth/

42. Fetal Growth Retardation/

43. Birth Weight/

44. Low Birth Weight.mp.

45. ((Preterm* or Premature*) adj2 (labo#r* or birth* or born or infant or baby or babies or child or children or girl*1 or boy*1)).mp.

46. Prematurity.mp.

47. IUGR.mp.

48. intrauterine growth restriction.mp.

49. or/36-48

50. 25 or 35

51. 49 and 50

52. Epidemiologic Studies/

53. exp case control studies/

54. exp cohort studies/

55. Case control.tw.

56. (cohort adj (study or studies)).tw.

57. Cohort analy*.tw.

58. (Follow up adj (study or studies)).tw.

© 2022 Kotecha SJ et al. *JAMA Pediatrics.*

59. (observational adj (study or studies)).tw.

60. Longitudinal.tw.

61. Retrospective.tw.

62. Cross sectional.tw.

63. Cross-sectional studies/

64. case-control studies/ or longitudinal/ or follow-up studies/ or prospective studies/

65. or/52-64

66. 51 and 65

The following table is an explanation of the symbols used in the search strategy above.

/	after an index term (MeSH heading) indicates that all subheadings were selected.
*	before an index term indicates that that term was focused - i.e. limited to records where the term was a major MeSH/Emtree term.
"exp"	before an index term indicates that the term was exploded.
.tw.	indicates a search for a term in title/abstract
.mp.	indicates a free text search for a term
#	retrieves records that contain the search term with substituted character(s) in the specified location.
*	at the end of a term indicates that this term has been truncated.
*n	The limited truncation symbol, $n, Retrieves records that contain the search term and all possible suffix variations of a root word with the maximum number of characters that may follow the root word or phrase, specified by n.
?	in the middle of a term indicates the use of a wildcard.
adj	indicates a search for two terms where they appear adjacent to one another

Databases and information sources

Bibliographic databases
CINAHL 2011-present
Embase 2011-present

© 2022 Kotecha SJ et al. *JAMA Pediatrics.*

HMIC Health Management Information Consortium 2011
Medline 2011-present
Medline in Process
Scopus
OpenSIGLE
Web of Knowledge<br>Science Citation Index Expanded 2011- present<br>Social Science Citation Index 2011- present<br>ISI Proceedings 2011- present

© 2022 Kotecha SJ et al. *JAMA Pediatrics.*

The effect of premature birth compared to term birth on later lung function - a systematic review of the literature

Source	Y/N/not clear/not reported/comment
Study ID number	
Report ID (surname of first author and year study undertaken)	
Title	
Authors names	
Journal	
Language published in	
Reviewed by	
Other comments	
Eligibility	
Confirm eligibility for review	
Gestation ≤ 32 weeks gestation	
Gestation 33-36 weeks gestation	
Other gestation please state	
Age at time of LF testing <5 years	
Age at time of LF testing ≥5 years	
If age less than 5 years method of LF testing	
LF variables collected $FEV_{0.5}$	
LF variables collected FEV_1	
LF variables collected FVC	
LF variables collected $FEF_{25-75\%}$/MEF	
LF variables collected Ratios	
LF variables collected TLC	
LF variables collected RV	
LF variables collected DL_{CO}	
LF values reported compared to predicted values or term group comparison	
BPD/CLD group	
Reason for exclusion	
Need to write to authors	
Other comments	
Methods	
Study design	
Age of study groups	
Other comments	
Participants	
Total number	
Total number in CLD/BPD group	
Total number in prem group	
Total number in control group	
Total number excluded	

© 2022 Kotecha SJ et al. *JAMA Pediatrics.*

Reason why excluded	
Setting	
Birthweight	
Social status	
Rate of ventilation	
Personal smoking by the prem subjects	
Age at time of LF testing	
Sex	
Weeks' gestation	
Maternal smoking	
Surfactant given and details	
Maternal steroids given and details	
Country	
Co-morbidity	
Ethnicity	
Year of birth of participants	
CLD/BPD and how defined	
Neonatal data information	
Date of study	
Other comments	
Interventions	
Did study consider intervention	
Specific intervention	
Intervention details	
Is baseline data adequate	
Other comments	
Outcomes	

$FEV_{0.5}$	Prem	BPD/CLD/	control
Total number in group			
Mean			
SD			
Median			
Significance			
Method of measuring LF			
Method of standardisation			
Raw values			

FEV_1	Prem	BPD/CLD/	control
Total number in group			
Mean			

© 2022 Kotecha SJ et al. *JAMA Pediatrics.*

SD			
Median			
Significance			
Method of measuring LF			
Method of standardisation			
Raw values			

FVC	Prem	BPD/CLD/	control
Total number in group			
Mean			
SD			
Median			
Significance			
Method of measuring LF			
Method of standardisation			
Raw values			

FEF_{25-75}/MEF	Prem	BPD/CLD/	control
Total number in group			
Mean			
SD			
Median			
Significance			
Method of measuring LF			
Method of standardisation			
Raw values			

ratios	Prem	BPD/CLD/	control
Total number in group			
Mean			
SD			
Median			
Significance			
Method of measuring LF			
Method of standardisation			

© 2022 Kotecha SJ et al. *JAMA Pediatrics.*

Raw values			

TLC	Prem	BPD/CLD/	control
Total number in group			
Mean			
SD			
Median			
Significance			
Method of measuring LF			
Method of standardisation			
Raw values			

RV	Prem	BPD/CLD/	control
Total number in group			
Mean			
SD			
Median			
Significance			
Method of measuring LF			
Method of standardisation			
Raw values			

DLCO	Prem	BPD/CLD/	control
Total number in group			
Mean			
SD			
Median			
Significance			
Method of measuring LF			
Method of standardisation			
Raw values			

Miscellaneous	
Funding source	
Key conclusions	
Miscellaneous comments from authors	

© 2022 Kotecha SJ et al. *JAMA Pediatrics.*

References to other relevant studies	
Other comments	

eAppendix 2

Assessment of study quality

Quality	Scores awarded	
Selection		
1) Representativeness of the exposed cohort		
a) truly representative of the average in the community	4	
b) Somewhat representative of the average in the community	3	
c) Selected group of users eg nurses, volunteers	2	
d) no description of the derivation of the cohort	1	
2) Selection of the non exposed cohort		
a) Drawn from the same community	3	
b) Drawn from a different source	2	
c) no description of the derivation of the non exposed cohort	1	
3) Ascertainment of exposure (weeks gestation)		
a) secure record (scan +/- LMP)	3	
b) Written self report (medical assessment)	2	
c) no description	1	
4) Demonstration that outcome of interest was not present at start of study		
a) yes	2	
b) no	1	
Outcome		
1) Assessment of outcome		
a) independent blind assessment	4	
b) record linkage	3	
c) self report	2	
d) no description	1	

© 2022 Kotecha SJ et al. *JAMA Pediatrics.*

2) Adequacy of follow up of cohorts		
a) complete follow up all subject accounted for	4	
b) subjects lost to follow up unlikely to introduce bias	3	
c) follow up rate low and no description of those lost	2	
d) no statement	1	

Data analyses

Where there are multiple papers, the most comprehensive publication will be included in the main analyses.

For any disagreement, SK will arbitrate the final decision.

The medians will be converted to means, wherever possible, using best current methods.

The data for meta-analyses will be entered into RevMan and forest plots will be produced. Heterogeneity will also be reported by RevMan, where there are high levels of heterogeneity random effects forest plots will be produced rather than fixed effects.

Meta-regression will be carried out in R or Stata as appropriate to report the effect of change over time or between factors of interest.

Reference

1. Kotecha SJ, Edwards MO, Watkins WJ, Henderson AJ, Paranjothy S, Dunstan FD, Kotecha S. Effect of preterm birth on later FEV1: a systematic review and meta-analysis. Thorax. 2013 Aug;68(8):760-6. doi: 10.1136/thoraxjnl-2012-203079. Epub 2013 Apr 20. PMID: 23604458.

© 2022 Kotecha SJ et al. *JAMA Pediatrics.*

eTable 4. Description of the included articles

Author, Year Published, Country	Quality Score	Objective	Study Design	Study Group	Control Group	Outcome Measure
Hagman, 2021 Sweden(1)	12	"Very preterm birth may be associated with lung function impairment later in life. It is not known if this is caused by prematurity per se or by associated perinatal events, such as maternal-foetal inflammation and severity of early neonatal lung disease. We assessed these factors in a prospective cohort of very preterm infants followed from birth to middle school age."	Prospective cohort	71 infants with a gestational age of median 27.4 (23.9-31.7 weeks). 39 without BPD, 32 with BPD. Pulmonary function in 53 children	No control group	Pro-inflammatory and modulatory cytokines were measured in umbilical cord blood and in arterial blood sampled at 6,24 and 72 h after birth<br>Pulmonary function tests FeNO
Hayden, 2018 USA (2)	12	"Hypothesized that gestational age, birthweight and neonatal lung disease severity will be predictors of long-term lung function in a cohort of former preterm infants with respiratory disease."	Cohort Study	157 preterm-born children. Moderate to severe BPD was present in 84%.	No control group	Lung function pre and post bronchodilator
Vrijlandt, 2018 Netherlands (3)	6	To determine the long-term effects of moderately preterm birth on respiratory health: that is respiratory symptoms, allergic symptoms and exercise capacity"	Community based prospective cohort	37 children born moderately preterm	34 full term	Prevalence of respiratory symptoms (ISAAC Questionnaires) Lung function Exercise tests (maximal workload etc.)
Arigliani 2020 Italy (4)	16	"Provided that Scond is frequently pathological in children with cystic fibrosis or asthma, even in the presence of normal LCI and FEV1, we tested the hypothesis that also in the extremely preterm population this index could be more sensitive than FEV1 to detect conductive airway impairment. A secondary aim was to investigate the relationship between Sacin and the transfer factor of the lung for carbon monoxide (TLco), reflecting intracinar regions."	Cohort	47 extremely preterm children, 40% (17/47) with BPD	60 full-term children	Spirometry TLCO MBW
Arroyas Sanchez, 2015 Spain (5)	6	"Main objective is to get to know the characteristics of our patients who were diagnosed with BPD and the possibility of developing altered lung function testing"	Retrospective study	Neonates born below 1500g and/or 32 weeks gestation 189 patients, 30 (15.8%) diagnosed with BPD (mild 46.6%, moderate 26.6%, severe 26.6%)	No control group	Echocardiography<br>Lung function and disease
Bar-Yoseph, 2019 Israel (6)	11	"Our aim was to evaluate airway hyper-reactivity, systemic inflammatory markers, allergic parameters and respiratory morbidity, 5-7 years following Palivizumab administration to children born at 29-32 weeks of gestation"	Cohort	42 preterm children. 27 received palivizumab (p+ group) 15 did not (p-group)	No term control group	Methacholine challenge test, serum inflammatory cytokines, fractional exhaled nitric oxide, blood tests for eosinophil count, IgE and assessment of respiratory morbidity by questionnaire

© 2022 Kotecha SJ et al. *JAMA Pediatrics.*

Bozzetto, 2016 Italy (7)	12	"The aim of this study was to assess HRQoL in adolescents with BPD, in comparison with age matched and sex matched control groups of healthy volunteers and asthmatic subjects"	Cross sectional design	27 BPD	27 healthy controls term born<br>27 asthmatic patients	HRQOL<br>Spirometry
Cardoen, 2019 Belgium (8)	13	"Many cross-sectional studies have assessed PFT in patients with BPD. However, only a few studies have assessed PFT longitudinally and none have done so using the Global Lung Initiative (GLI) reference values that offer seamless transition from childhood to adulthood. Differences in PFT evolution between old and new type BPD have also not been reported. We therefore set up this retrospective cohort evaluation."	Retrospective observational cohort study	24 patients with BPD 11 early BPD cohort 13 in late type BPD cohort	No control group	Longitudinal lung function<br>Reversibility
Cazzato, 2013 Italy (9)	18	"To evaluate the long-term outcome and the potential risk factors which are likely to influence lung function in very low birth weight (VLBW, birthweight =< 1,500 g) children with or without BPD, born at a gestational age of 32 weeks or less at a single tertiary center during 1996-1999, after the introduction of antenatal steroids and surfactant therapy together with less aggressive ventilator support"	Observational cohort	48 children BW =< 1500g and GA =< 32 weeks - 22 with BPD, 26 without	46 children with BW >2500g and GA >37 weeks born in the same period, recruited from among patients with transient benign non-thoracic diseases discharged from the same hospital	Lung function (Spirometry, DLCO, FeNO)<br>Respiratory symptoms<br>Skin prick testing
Chang 2020 Taiwan (10)	15	"To better understand the importance of early pulmonary insults, we conducted this prospective, observational study to evaluate lung function in a cohort of VLBW preterm survivors at preschool age (5-6 years old). The aim of this study was to compare lung function in a group of former preterm-born children to that of a group of control children born at term with normal birth weight. We also examined the associations between gestational age (GA), birth weight (BW), and the presence of BPD in regards to lung function. Furthermore, we investigated whether the severity of BPD influences lung function"	Prospective, observational study	85 VLBW preterm children born <37 weeks gestation and <1.5kg 73% BPD	29 term controls >/= 37 weeks and BW >2.5kg	Lung spirometry<br>Questionnaire
Choukroun 2013 France (11)	15	"To establish the main antenatal, neonatal and early childhood respiratory correlates of respiratory status in school-aged children born at ≤32weeks of gestation"	Cohort	Ex-preterm children born at ≤32 weeks gestation between 1997 and 2001 at Bordeaux University Hospital. Exclusion criteria included congenital abnormalities or major neurological impairment (children with cognitive impairment, physical limitation due to cerebral	None	Respiratory questionnaire<br>Lung function (spirometry, exercise induced BDR, plethysmography, exercise challenge, DLCO)

© 2022 Kotecha SJ et al. *JAMA Pediatrics.*

				palsy, major surgery during childhood, additional comorbidity which may contribute to respiratory morbidity such as severe reflux, dysfunctional swallowing, cardiovascular malformation.		
Cristea, 2014 USA (12)	12	While physiologic measures have been described for children who were prematurely born, limited data exists regarding the spirometric indices in those patients with severe BPD who were previously ventilator dependent at home. We hypothesized that flow limitation would be present in these patients and these findings would persist over time.	Retrospective cohort study	History of BPD and ventilator-dependency via tracheostomy at home 19 patients of original cohort of 102	N/A	Lung function (spirometry)
Crowther, 2016, Australia (13)	17	To assess if exposure to repeat dose(s) of antenatal corticosteroids has beneficial effects on neurodevelopment and general health in mid-childhood, at 6 to 8 years' corrected age.	Randomized control trial	Repeat dose antenatal intramuscular betamethasone in those at risk of very preterm birth (<32 weeks) Doses repeated weekly up to 32 weeks gestation Repeat steroids: n = 430 Placebo: n = 426	Saline placebo	Health assessment and physical examination<br>Lung function<br>Assessment of gross and fine motor function<br>Psychological assessment<br>Visual assessment
De Sousa Sena, 2013, Canada (14)	7	"Therefore, this study aims to evaluate the profile of physical activity in daily life (PADL) in young adults survivors of BPD and compare it with public guidelines for PA"	Sub study of a large cohort study	11 subjects with BPD	No control group	Lung function<br>Maximal and submaximal exercise capacity (incremental peak and endurance cycling test)<br>PADL
Debevec, 2019, Slovenia (15)	13	The purpose of this study was to determine the effects of acute hypoxia on poikilocapnic HVR and exercise performance in healthy, active pre-term born adult males and compare them to their age and aerobic capacity matched counterparts born at full-term.	Cohort	Male and Gestation ≤ 32 weeks and Birth weight ≤ 1500 g and Hyperoxic treatment at birth	Male and Gestation ≥ 38 weeks and Birth weight ≥ 2500 g	Lung function Hypoxia sensitivity test Exercise test Blood sampling and biochemical analysis
Devakumar, 2015, Nepal (16)	19	"We followed up the cohort at ~8 years of age (an age when the vast majority of children can perform spirometry satisfactorily) to investigate whether a prenatal micronutrient supplementation that increased birth weight was also associated with increased lung function during childhood"	Follow up of a double-blind randomised control trial	101 preterm births total, 58 with lung function data at 8 years	1038 term births, 793 with lung function data at 8 years	Lung function questionnaire data anthropometry
Doyle, 2017, Australia (17)	16	The aim of our study was to compare changes in assisted ventilation and oxygen therapy administered during the newborn period and in lung function at 8 years	Longitudinal cohort study	Three study groups of preterm infants born <28 weeks gestation based on	No control population	Lung function (spirometry) 1991/1992: Spirometry, plethysmography 1997:

© 2022 Kotecha SJ et al. *JAMA Pediatrics.*

		of age in children in Victoria, Australia, whose birth was extremely premature over three distinct periods, all of which followed the introduction of exogenous surfactant into clinical practice.		year of birth - 1991/1992 (Data captured in 2013 SR paper) - 1997 - 2005 BPD defined as oxygen dependence at 36 weeks corrected.		Spirometry, plethysmography 2005: Spirometry, health questionnaire
Doyle, 2017, Australia (18)	16	To determine if children born with birth weight less than 1,251 g who were treated with neonatal caffeine had improved respiratory function at 11 years of age compared with children treated with placebo	Randomised controlled trial	Children with a birth weight < 1251g randomised to receive caffeine citrate less than 10 days after birth	Children randomised to receive placebo	Lung function (spirometry, BDR) Data also collected for CAP study includes academic performance, motor performance, behavioural assessment
Flahault, 2020, Canada (19)	12	This study aimed to determine the relationship between cardiovascular risk factors in young adults born ≤29 weeks GA. We further sought to investigate whether or not inflammation and oxidative stress were increased and associated with cardiovascular risk factors in this population, compared to full-term controls.	Cohort	Adults born very preterm ≤ 29 weeks. Exclusion criteria included severe neurocognitive impairment and pregnancy.	Adults born at term ≥37 weeks	Blood pressure<br>Glucose metabolism<br>Adiposity<br>Lipids<br>Respiratory function
Fortuna, 2016 Italy (20)	14	"Recent advances in perinatal care and neonatal respiratory therapy have led to a new phenotype of bronchopulmonary dysplasia ("new BPD"). The long-term respiratory outcome of this new form of BPD has yet to be adequately described. Aim of this study was to provide longitudinal data on lung function of an unselected cohort of children born extremely preterm with an extremely low birthweight in the post surfactant era"	Longitudinal	48 children born <28 weeks gestation and with a birthweight< 1,000g 28 had BPD	27 term children	Spirometry<br>FeNO<br>Respiratory symptoms
Gaffin, 2020 USA (21)	10	"Here we further aimed to test the hypothesis that exposure to poor indoor air quality disproportionately affects asthma morbidity in children born prematurely"	Longitudinal	47 preterm <37 weeks gestation	250 term children	Spirometry<br>Questionnaire<br>FeNO
Goncalves, 2006 Brazil (22)	12	The objective of this study was to analyse the prevalence of symptoms of asthma and atopic diseases, allergic sensitisation and altered pulmonary function and to determine the factors associated with altered pulmonary function in children and adolescents who were born prematurely.	Cross-sectional	Children and adolescents aged 6-14 years who were born at <37 weeks of gestation and birth weight <2000 g and were outpatients at Federal University of San Paulo between January 2008 and May 2011. Patients with major congenital malformations or acute respiratory disease over the previous 15 days were excluded from the study. n = 84	N/A	Skin prick testing<br>Lung function (Spirometry)<br>Symptom questionnaire

© 2022 Kotecha SJ et al. *JAMA Pediatrics.*

Gough,<br>2013<br>United Kingdom (23)	16	Report on respiratory symptoms, lung function and health status in adult survivors of BPD compared with non-BPD preterm and full-term controls.	Cohort	129 preterm adult survivors born Jan 1978 to April 1993 and cared for in the Royal Maternity Hospital, Belfast UK	Preterm control group: Cared for in the same NICU but did not develop BPD or receive mechanical ventilation or prolonged respiratory support<br>Full term control group: Born in same hospital without evidence of respiratory difficulties during hospital stay	Health questionnaire<br>Lung function (Spirometry)
Greenough,<br>2011<br>UK (24)	12	"The aim of this study was to determine whether respiratory morbidity, lung function, healthcare utilisation and cost of care at school age in prematurely born children who had bronchopulmonary dysplasia were influenced by use of supplementary oxygen at home after neonatal intensive care unit discharge."	Cohort	160 preterm children with BPD, 65 of them had received supplementary oxygen when discharged home. 95 no home oxygen 84 of the 160 agreed to and had analysable lung function tests	No control group	Healthcare utilisation and cost of care respiratory morbidity (parent-completed questionnaire) Lung function
Greenough,<br>2014<br>UK (25)	15	"To compare respiratory and functional outcomes of school-age children born extremely prematurely who received either HFO or CV immediately after birth to test the hypothesis that the use of HFO would be associated with superior small airway function at school age without adverse effects"	Follow up of randomised trial	319 children. 159 CV, 160 HFO. 121 CV had LF results, 129 HFO had full lung function results	No term group	Lung function<br>Echocardiography<br>Examinations and respiratory health related quality of life and functional assessment questionnaires
Hadchouel<br>2018<br>France (26)	16	"We studied lung function in relation to asthma symptoms from birth in adolescents born very preterm and in controls from the Etude EPIdémiologique sur les Petits Ages Gestationnels (EPIPAGE) cohort"	Prospective observational population-based cohort	304 22-32 weeks gestation 273 completed prebronchodilator spirometry	47 term born at 39 and 40 weeks 44 performed complete prebronchodilator spirometry	Lung function questionnaire FeNO
Hamon,<br>2013<br>France (27)	13	"The aim of this study was to describe the characteristics of, and potential determinants to, the airway response to exercise in children born extremely preterm"	Cross sectional study	42 non asthmatic non atopic children born <32 weeks gestation	27 healthy non asthmatic non atopic term children	"Spirometry and respiratory impedance were measured at baseline and repeated after a single-step 6-min treadmill exercise in a climate-controlled room"

© 2022 Kotecha SJ et al. *JAMA Pediatrics.*

Hayden,<br>2018<br>USA (28)	11	" We hypothesize that gestational age, birth weight and neonatal disease severity will be predictors of long-term lung function in a cohort of former preterm infants with respiratory disease"	Cohort	239 preterm children, moderate to severe BPD in 82%	No control group	spirometry
Hirata<br>2015<br>Japan (29)	17	"To assess lung function at 8 years old in extremely low birthweight (ELBW) survivors and to identify perinatal determinants associated with impaired lung function."	Retrospective cohort study	201 ELBW <1001g; 82 had BPD 40.8%	No control group	Lung function
Kaczmarczyk<br>2017<br>Poland (30)	12	"To evaluate the long-term impact of preterm birth on respiratory function in female patients born preterm, we undertook spirometric examinations twice, as they reached the age of puberty, then follow-up examinations of part of the same cohort in adulthood. We sought evidence that preterm birth is correlated with poorer spirometric results into adulthood."	Longitudinal	70 girls and then in adulthood 12 of those were retested born <2.5kg or <37 weeks preterm	Adult group compared to 28 females born >2.5kg at term adolescent group compared to population normal	Spirometry
Karnaushkina,<br>2017<br>not stated authors from Russia (31)	7	"The aim of the investigation was to study the impact of prematurity and severe infections of lower respiratory tract suffered in early childhood on the occurrence of chronic bronchopulmonary pathology in young adults."	A controlled retrospective study "case control"	"16 adult patients born prematurely (n=10) or suffered infections of lower respiratory tract in early childhood (n=6)",	9 healthy volunteers as a control group	"All patients filled in British Medical Research Council (mMRC scale) questionnaires of breathlessness rating, underwent spirometry with bronchodilatation test, 6-minute walk test with determination of blood oxygen saturation level, body plethysmography and examination of lung diffusion capacity."
Kilbride,<br>2012<br>not stated, authors from USA(32)	7	"To assess the impact of preterm birth on childhood pulmonary function and oxygen consumption measurements. Secondary objective is to assess levels of exhaled nitric oxide in preterm versus term children and relationship to clinical findings."	Follow up of children enrolled in neurodevelopment preterm follow-up program	12 ELBW<br>18 heavier preterm born children	9 terms born at normal birthweight	Exhaled nitric oxide, Pulmonary function and treadmill exercise testing
Kilbride,<br>2019<br>USA(33)	11	"The goal was to determine if inhaled nitric oxide (iNO) for 3 weeks during neonatal care of high-risk preterm infants was associated with improved pulmonary function and exercise capacity or altered exhaled nitric oxide (FeNO) levels in later childhood."	Follow up trial	34 very preterm children </= 1.25kg </= 32 weeks gestation. 18 NO group 16 placebo 32 completed spirometry 17 in NO group 15 in placebo group	No term control group	Pulmonary function testing<br>Exercise testing<br>FeNO<br>Impulse oscillometry
Konefal,<br>2013<br>Poland(34)	14	"To assess whether school-age spirometry and lung volume outcomes of preterm infants with history of moderate to severe respiratory distress syndrome (RDS), born in the surfactant era and treated with conventional mechanical ventilation (IMV) and discharged home with or without the diagnosis of BPD (chronic lung disease of prematurity), differ from those of term neonates (controls)."	Cohort	38 preterms no BPD 20 preterms with BPD	90 terms	Spirometry

© 2022 Kotecha SJ et al. *JAMA Pediatrics.*

Kotecha,<br>2012<br>UK(35)	15	"In this study we sought to compare lung function at 8-9 and 14-17 years in a population-based cohort of children born late preterm at 33-34 weeks gestation and near term at 35-36 weeks gestation with children of similar age born at term (>=37 weeks gestation). We also compared the results for these two gestation groups with children of similar age born extremely preterm at 25-32 weeks gestation. The longitudinal change in lung function between 8-9 and 14-17 years of age is also reported."	Cohort Sub-group of Avon Longitudinal Study of Parents and Children	All births from the Avon Longitudinal Study of Parents and Children who had lung spirometry at 8-9 years of age and/or 14-17 years. Children divided into four groups based on gestational age: 25-32w, 33-34w, 35-36w, and >=37-43w	>=37-43 weeks group	Lung function (spirometry)<br>Bronchial challenge
Yen-Ping Kung<br>2021<br>Taiwan(36)	14	"This study aimed to investigate both in utero exposure and childhood exposure to PFAS and the relationships between them and lung function development in childhood"	Prospective birth cohort study	8 preterm	157 term	Lung function<br>Questionnaires
Kwinta<br>2013<br>Poland(37)	15	"To determine if ELBW infants are at higher risk for the development of allergic and respiratory symptoms and to establish if there were any specific risk factors for these symptoms"	Cross sectional observational study	81 children born ELBW<br>"Spirometry was performed in 56% (45/81) of ELBW children and in 80% (32/40) of children in the control group. In 44% of ELBW children it was not possible to perform the spirometry because of their neurological complications or lack of child's cooperation. A quarter of the ELBW group (22/81) and half of the control group (20/40) were able to perform acceptable and repeatable spirometry that conformed to ATS/ERS standards for this age group of children. "	40 full term children spirometry in 32/40<br>"Spirometry was performed in 56% (45/81) of ELBW children and in 80% (32/40) of children in the control group. In 44% of ELBW children it was not possible to perform the spirometry because of their neurological complications or lack of child's cooperation. A quarter of the ELBW group (22/81) and half of the control group (20/40) were able to perform acceptable and repeatable spirometry that conformed to ATS/ERS standards for this age group of children. "	The children were examined for clinical signs of allergy, and were subjected to the following tests: serum total IgE, skin prick tests (SPT), exhaled nitric oxide measurement (FeNO) and spirometry.

© 2022 Kotecha SJ et al. *JAMA Pediatrics.*

Lasry, 2021 (38) Canada	11	“This study was aimed to describe the cardiopulmonary profiles of adult patients with bronchopulmonary dysplasia (BPD), comparing them to normative adult values.”	Single centre retrospective cohort study	44 preterm born (<=33 weeks) adults with BPD from adult BPD clinic	None	Spirometry, echocardiography
Landry, 2016 Canada(39)	12	"To describe the quality of life, pulmonary lung function, bronchial hyperresponsiveness, body composition, and trends in physical activity of adults born prematurely, with or without respiratory complications”	Cohort study	26 preterm subjects with no neonatal respiratory complications 31 preterm subjects with neonatal RDS 31 preterm subjects with BPD	35 term born	Health related quality of life Respiratory health Pulmonary function Methacholine challenge Sedentary behaviour physical activity level
Lista, 2013 Italy (40)	10	To investigate respiratory health and lung function in school-aged children without BPD, who were very low birth weight and randomized at birth to high frequency oscillatory ventilation (HFOV) or volume guarantee (VG) ventilation for severe respiratory distress syndrome.	Observational study	7-y-old ex-preterm infants with severe RDS, randomly assigned at birth to receive assisted/control ventilation + volume guarantee (VG Group) or high frequency oscillatory ventilation (HFOV Group)	No control group	Lung function (Spirometry + BDR, Plethysmography, Airway Resistance) Respiratory questionnaire data
Luo, 2019 Taiwan(41)	14	To examine the relationship between neonatal respiratory status and longitudinal respiratory health outcomes in preterm infants with very low birth weight (VLBW, birth weight <1500 g).	Prospective cohort	GA < 32 weeks Birth weight < 1500 g Absence of congenital or genetic anomalies Being born in a participating hospital or admitted to one shortly after birth 36 (of 55 total) children had PFT at 4 years of age	None	Toce scoring Lung function (Spirometry) 6 minute walk test
MacBean 2018 UK (42)	11	"To determine the impact of viral lower respiratory tract infections (LRTIs) in infancy including rhinovirus (RV) and infancy respiratory syncytial virus (RSV), on school age pulmonary function and healthcare utilization in prematurely born children"	Prospective study	51 children. 21 no LRTI. 14 RV LRTI, !) RSV LRTI and another viral LRTI 33 had spirometry	No control group	Pulmonary function Health related costs of care from aged one to follow up determined.
Maclean 2016 Canada(43)	15	This study examined respiratory mechanics and ventilatory response during exercise in a large cohort of children born extremely preterm (EP). Children diagnosed with non-ambulatory CP or legal blindness at 18 months were excluded due to potential physical limitations to completing exercise testing.	Cohort study	Children 8-12 years of age who were born =< 28 weeks between 1997 and 2004 and treated in a large regionalised neonatal intensive care unit in western Canada. Children categorised as no/mild BPD or moderate/severe BPD based on definitions of NICHD workshop.	Age matched control children with no significant history of cardiorespiratory disease based on parental report. Recruited as friends of EP children, and through posters as well of word of	Cardiorespiratory health questionnaire Lung function (spirometry, DLCO)

© 2022 Kotecha SJ et al. *JAMA Pediatrics.*

					mouth within the community.	
Molgat-Seon, 2019 Canada(44)	13	"The purposes of the present study were to 1) quantitatively assess the shape of the MEFV curve in adults born preterm with and without BPD, and healthy adults born at full-term, 2) identify where along the MEFV curve differences in shape existed between groups, and3) determine the association between an index of MEFV curve shape and characteristics of preterm birth (i.e., gestational age, mass at birth, duration of oxygen therapy) in those born preterm with and without BPD"	Cohort	Adults born preterm (≤32w) with BPD and without BPD	Adults born at term	Lung function (spirometry, plethysmography) MEFV curves
Morata-Alba, 2019 Spain(45)	16	This study aimed to determine the prevalence of asthma at 6-8 years of age, as well as the factors which may protect against it, in a group of moderate-to-late NBs delivered between 32 weeks +1 day and 35 weeks + 0 days	Prospective cohort study	Preterm Infants n = 116 GA: 32 + 1 to 35 + 0 Exclusion criteria: Chronic lung disease (including BPD), airway, lung, and or GI malformations, congenital heart disease, chromosomopathies, immunodeficiencies, chronic neurological, renal, and/or gastrointestinal diseases, or any other condition that might be associated with an increased risk of respiratory morbidity.	Full term infants n = 116 GA: >= 37 weeks	Lung function<br>FeNO<br>Skin prick testing Respiratory questionnaire
Moreno-Galdo 2020 Spain(46)	10	"Data addressing short- and long-term respiratory morbidity in moderate- late preterm infants are limited. We aim to determine the incidence of recurrent wheezing and associated risk and protective factors in these infants during the first 3 years of life."	Prospective multicenter birth cohort study	977 moderate preterm infants, 670 completed all 3 years of follow up. 187 of which had accurate spirometry	No control group	Wheeze<br>Allergen sensitization<br>Pulmonary function
Morris, 2018 not stated(47)	7	"To determine according to BPD status, the lung function of 16- to 18-year-old, very prematurely born young people (<29 weeks of gestational age) of whom more than 90% were exposed to both antenatal steroids and postnatal surfactant"	Not stated	59 preterm children of whom 34 had BPD	No control group	Spirometry<br>DLCO
Morsing, 2011 Sweden(48)	19	"To assess lung function at early school age in children delivered at very early gestation owing to intrauterine growth restriction and abnormal foetal blood flow (IUGR)."	Cohort	31 children born preterm with IUGR (PT-IUGR) 21 with BPD 31 children born preterm with appropriate BW (PT-AGA) 9 with BPD	31 children born at term (T-AGA) PT-AGA and T-AGA matched for gender and year of birth	Lung function<br>FeNO<br>Questionnaire
Narayanan 2013	11	"To examine whether alveolar damage in extreme-preterm survivors persists into late childhood, we compared alveolar	Cross sectional	21 mild preterm (32-36 wk) 19 extreme preterm	61 term-born (37-42 weeks)	"Lung function using spirometry and

© 2022 Kotecha SJ et al. *JAMA Pediatrics.*

UK(49)		dimensions between schoolchildren born term and preterm, using hyperpolarized helium-3 magnetic resonance."		(<32 wk, not oxygen dependent at 4 wk;) 18 extreme preterm with chronic lung disease (<32 wk and oxygen dependent beyond 4 wk).		plethysmography. Apparent diffusion coefficient, a surrogate for average alveolar dimensions, was measured by helium-3 magnetic resonance." questionnaire
Nasanen-Gilmore 2018 Finland(50)	17	To assess lung function and pulmonary health in adults born preterm across the whole gestational age range	Cohort	Early preterm <34 weeks Late preterm 34 to <37 weeks	Term ≥37 weeks	Lung function (spirometry, BDR)
Nixon, 2013 USA(51)	13	"To compare asthma history and pulmonary function in adolescents born prematurely with very low birth weight with and without antenatal steroid exposure"	Cohort	188 preterm children born VLBW <1500g 94 exposed to ANCS of which 23 had BPD. 85 had spirometry 94 unexposed to ANCS of which 26 had BPD. 75 had spirometry	No control group	Spirometry Asthma
Nixon, 2011 USA(52)	12	"To investigate the effects pf postnatal dexamethasone treatment on aerobic fitness and physical activity levels in school-aged children born with very low birthweight (VLBW)"	Follow up study of a randomized control trial	65 preterm born infants' birthweight <1501g 37 dex 28 placebo BPD 49% of dex group and 71% placebo group	No control group	Aerobic fitness Physical activity questionnaire
Nordlund, 2017 Sweden(53)	14	"The objective of this study was to describe hallmarks of BPD at school age in comparison to children with atopic asthma."	Cross-sectional comparative study	30 children diagnosed with BPD. 13 mild, 17 moderate/severe	30 age and sex matched children with asthma and sensitized to airborne allergens	FeNO lung function bronchial provocation with methacholine respiratory symptoms asthma control test
Panagiotounakou,. 2019 Greece(54)	13	The primary aim of the study was to evaluate respiratory function of very preterm neonates at school age by performing spirometry and to compare these measurements with term controls. As a secondary aim, it was decided to assess the growth and long-term pulmonary outcome as expressed with the frequency of lower respiratory tract infections and hospital readmissions up to 8-10 years of age. Furthermore, the impact of the presence of BPD on lung function in school age was investigated.	Cohort	Very preterm neonates with birth weight (BW) <1500 g and gestational age (GA) <32 weeks, without congenital abnormalities, admitted to NICU during the study period	Term neonates hospitalized in level 1 NICU for maximum 4 days in the same period	Lung function Growth Questionnaire
Prais, 2015 Israel(55)	15	This study sought to assess the short- and long-term effects of palivizumab immunization on respiratory morbidity and pulmonary function at school age in children born extremely prematurely	Prospective cross sectional	Children 7-10 years Born at Schneider Children's Medical Center of Israel between 2000 and 2003 Gestation < 29 weeks Received palivizumab	Same as study but did not receive palivuzumab	Lung function (spirometry, plethysmography, DLCO) FeNO Methacholine challenge
Praprotnik, 2015 Slovenia(56)	14	"To examine respiratory morbidity and exercise capacity at school age in children after bronchopulmonary dysplasia (BPD)."	Cohort	Former preterm children after BPD (n = 23), without BPD (n = 33)	Term controls (n = 33) birth weight	Respiratory health questionnaire,

© 2022 Kotecha SJ et al. *JAMA Pediatrics.*

					>2800g >37 weeks gestation	Spirometry and six-minute walk test (6MWT), FeNO
Prenzel, 2020 Germany(57)	14	To assess the exercise capacity, exercise habits, and lung function of preterm born children with bronchopulmonary dysplasia (BPD) compared to term born controls at school age.	Single center case control study	Children born <37 weeks Admitted to NICU at University of Leipzig Very low birth weight (<1500g) Diagnosis of BPD (O2 >28 days)	Age matched (±6 months) term born	Lung function (spirometry, exercise induced bronchoconstriction) CPET (cycle ergometer)
Ronkainen 2015 Finland(58)	15	To investigate whether preterm birth, BPD, and the severity of BPD predict lung function in school children that are born in the surfactant era	Cohort	Children born very preterm ≤32 weeks gestation	Age and sex-matched controls born at term	Lung function (spirometry, plethysmography, DLCO) Exercise induced bronchoconstriction
Ruf, 2019 Germany(59)	14	The objective of this study was to compare exercise capacity and habitual physical activity between children born very and extremely preterm with and without BPD and term-born children	Cohort	GA <32 weeks and BW <1500g treated in hospital between 1997 to 2001	"15 healthy children born at term between 1997 and 2001 with an uneventful neonatal period served as controls. These were friends or siblings of the included preterm children to reduce selection bias. At the time of assessment, participants were aged 7-12 years."	Lung function (spirometry, plethysmography, airway resistence, DLCO) Exercise testing (accelerometry, CPET) Cardiac (ECG, echo)
Simpson, 2017 Australia(60)	17	To obtain comprehensive data on lung structure and function in mid-childhood from survivors of preterm birth. We aimed to explore relationships between lung structure, lung function and respiratory morbidity as well as early life contributors to poorer respiratory outcomes.	Prospective cohort	Children born preterm (≤32 weeks) with BPD and Children born preterm (≤32 weeks) without BPD	Healthy term controls born ≥37 weeks with no lifetime history of cardiopulmonary disease or recurrent respiratory symptoms	Lung function (spirometry, multiple breath washout, DLCO, FOT) CT chest
Sorensen, 2018 Denmark(61)	16	"Ex-premature school children show mild-to-moderate airway obstruction and decreased CO diffusing capacity. Multiple breath nitrogen washout (N2MBW) and NO diffusing capacity (DLNO) measurements may provide new insight into long-term pulmonary and vascular impairment in bronchopulmonary dysplasia (BPD)."	Single center cross sectional population based observational study	Gestational age <28 weeks or birthweight < 1500g who were born at Copenhagen University Hospital (Rigshospitalet) between 2002 and 2006	Age matched term born (>37 weeks and birth weight >2500g) children with no known respiratory conditions	Lung function (MBW, spirometry, plethysmography, DLCO/DLNO, BDR) FeNO Health questionnaire Echocardiogram

© 2022 Kotecha SJ et al. *JAMA Pediatrics.*

					(including asthma) or heart conditions	
Teig<br>2012<br>Germany(62)	15	"To test whether chronic bronchial inflammation may be a contributing risk factor for persistent airflow limitation in children born before 32 weeks of gestation in later life"	Cohort	34 preterm children spirometry reported for 16. 2 had BPD	18 term children spirometry reported for 11	Spirometry<br>Induced sputum<br>Questionnaire
Thunqvist,<br>2018<br>Sweden(63)	16	"The aim of the study was to determine lung function and airway mechanics in school-aged children born in 2004 to 2007 and extremely preterm (after 22-26 weeks of gestation)."	Population based cohort	153 had lung function assessment extremely preterm 22-26 weeks 90% had BPD Spirometry data for 90 extremely preterm 98 controls Oscillometry data for 151 extremely preterm 153 controls	157 had any assessment of lung function term	Spirometry<br>Impulse oscillometry<br>Questionnaire
Thunqvist,<br>2016<br>Sweden(64)	15	"We therefore investigated lung function at early school age and adolescence among children born moderately preterm"	Prospective birth cohort	149 preterm 32-36 weeks gestation	2472 terms	Lung function<br>Asthma symptoms
Tukova,<br>2020<br>Czech Republic(65)	15	The aim of this study was to evaluate the effect of early inhaled corticosteroids on chronic respiratory morbidity.	Cohort included in study	59 survivors enrolled in the Neonatal European Study of Inhaled Steroids. 48 underwent spirometry	No term control group	Respiratory morbidity during the 1st 2 years of life<br>Lung function at 5 years of age
Turner,<br>2013<br>UK{(66)	Data from author					
Um-Bergstrtom,<br>2018<br>Sweden(67)	15	" We hypothesized that pulmonary outcomes and health-related quality of life (HRQoL) were different in adults born preterm with and without a history of BPD compared to asthmatics and healthy individuals."	Cohort	26 preterms BPD </=32 weeks gestation 23 preterms no BPD </= 32 weeks gestation	23 asthmatic terms<br>24 Healthy terms	Lung function<br>HRQoL
Vardar-Yagli,<br>2015<br>Turkey(68)	7	"The aim of this study was to compare functional capacity and peripheral muscle strength in preterm children with bronchopulmonary dysplasia (BPD) with those of age-matched full-term healthy controls."	Cross sectional study	18 BPD <32 weeks and/or birthweight under 1,500g	20 Healthy subjects born full term	Pulmonary function<br>Body composition by skinfold method<br>Muscle strength<br>Functional capacity
Vollsaeter,<br>2015<br>Norway(69)	17	Compare respiratory health in children born extremely preterm (EP) or with extremely low birthweight (ELBW) nearly one decade apart, hypothesizing that better perinatal management has led to better outcome.	Cohort	GA <28w or birth weight < 1000g treated at one of two regional NICUs (Bergen and Stavanger)	For each EP-born participant of both birth-cohorts, the next-born child in the same maternity ward of the same gender with GA>37 weeks and BW>3000 grams were identified from birth protocols and	Lung function (spirometry, plethysmography, DLCO, FeNO, Methacholine challenge, BDR, airway resistance)<br>Respiratory questionnaire

© 2022 Kotecha SJ et al. *JAMA Pediatrics.*

					invited as control. If that individual declined, the next-born eligible child was invited until a match was obtained	
Vollstaeer, 2013 Norway(70)	16	To assess the development of spirometric lung function variables from mid-childhood to adulthood after extreme preterm birth	Population based cohort	Gestation ≤28 weeks or with birth weight ≤1000g Two separate groups: Group 1. Age 10 Group 2. Age 18	Age matched term born controls, recruited from same hospital as children born preterm	Lung function (spirometry)
Vrijlandt, 2018 The Netherlands(71)	14	"To determine the long-term effects of moderately late preterm (MLP) birth on respiratory and allergic symptoms, lung function, and exercise capacity in adolescence."	Prospective cohort	37 Moderate-Late Preterms	34 full terms	Questionnaire spirometry exercise measures
Winck, 2016 Brazil(72)	15	"To compare somatic growth, lung function, and level of physical activity in schoolchildren who had been very-low-birth-weight preterm infants (VLBWPIs) or normal-birth-weight full-term infants."	Case-control study	48 VLBW preterm born </= 1.5kg	45 normal birthweight term infants >/= 2.5kg	Anthropometric measurements Spirometry Questionnaire regarding physical activity
Yaacoby-Bianu, 2019 Israel(73)	12	"We hypothesized that former late preterm children have abnormal physiology parameters, including uneven ventilation distribution, due to premature disruption of normal lung development"	Cross sectional study	29 late preterm children born at 34-36.6 weeks gestation	30 term children born >37 weeks gestation and >2500g	Spirometry multiple breath washout measurement by lung clearance index 6 minute walk test Symptoms related to asthma and allergy Godin Leisure time exercise questionnaire
Yang, 2020 New Zealand(74)	17	" We hypothesized that VLBW adults would have impaired lung function compared with controls, and those with a history of BPD would have worse lung function than those without"	Cohort	226 VLBW survivors of the New Zealand VLBW cohort. 20% had BPD. 224 had spirometry	100 term controls	Spirometry Plethysmorgraphic lung volumes Diffusing capacity of the lung for carbon monoxide Single-breath nitrogen washout
Hacking, 2013 Australia (75)		"To determine if respiratory function at 8 years of age in extremely low birth weight (ELBW; birth weight <1,000 g) or extremely preterm (EPT, <28 weeks' gestation) children born in 1997 remains worse than normal birth weight (NBW; birth weight, >2,499 g) and term (37–42 weeks) controls, particularly in those ELBW/EPT children who had bronchopulmonary dysplasia (BPD). "	Cohort	150 ELBW/EPT 56 with BPD, 94 without BPD	149 term controls	Spirometry ISAAC questionnaire

© 2022 Kotecha SJ et al. *JAMA Pediatrics.*

Aoyama, 2020, USA (76)	14	"To identify potential modifiable risk factors for the development of impaired lung function in patients with a history of prematurity and bronchopulmonary dysplasia."	Retrospective cohort study	88 children born ≤36 weeks recruited from BPD clinic	None	Spirometry
Arroyas, 2020, Spain (77)	14	"Our main objective was to study respiratory evolution and pulmonary and cardiac function in adolescents born preterm in the post-surfactant era."	Observational cross-sectional study	74 adolescents born very preterm (<32 weeks) and 74 age matched adolescents born 32-36+6 weeks	None	Spirometry, FeNO, Blood pressure, Skin prick testing, echocardiogram
Durlak, 2021, Poland (78)	14	"We aimed to evaluate respiratory morbidities and lung function of very low birth weight (VLBW) Polish children followed up at the age of 7 years old"	Cross sectional study	40 VLBW children, 11 BPD, 29 No BPD	30 term controls	Impulse oscillometry, spirometry, electrical impedance segmentography
Filippo, 2021, Italy (79)	17	"To assess the effect of prematurity on respiratory outcomes in children born ≤32 weeks of gestational age at 11 years of age."	Cross sectional study	55 preterm born children (≤32 weeks)<br>5 with BPD, 50 No BPD	55 Term born	Lung function, FeNO, blood sample for eosinophil count, skin prick testing
Hart, 2021, UK (80)	18	"We prospectively studied the largest population to date of children aged 7 – 12 years who were born at ≤34 weeks of gestation to specifically identify those with low spirometry to permit identification of which early-life factors are associated with lung function deficits associated with preterm-birth."	Prospective cohort	544 preterm-born children, 141 with low lung function, 41 (29%) with BPD, and 403 with normal lung function, 67 (16%) with BPD.	195 term-born children.	Spirometry, FeNO and bronchodilator reversibility testing.
Joshi, 2013, UK (81)	15	"In this study, we compared exercise capacity in similarly aged preterm-born children with and without CLD and term-born children. We also measured airway function after exercise and after postexercise bronchodilator administration to assess whether these preterm-born children had exercise induced bronchoconstriction, and whether this was reversible by a b2 bronchodilator administered after exercise."	Cohort study	92 children, 62 preterm-born (29 with CLD)	30 term born children	Spirometry pre and post exercise testing and bronchodilator reversibility
Naples, 2021, UK (82)	10	"To evaluate the feasibility of using optoelectronic plethysmography (OEP) to measure the ventilatory response to exercise in school - aged children born preterm, specifically assessing for evidence of dynamic hyperinflation."	Cross sectional study	20 11–15 year-olds born < 32 weeks gestation<br>16 moderate/severe BPD, 4 no/mild BPD	None	spirometry, body plethysmography, gas transfer testing, optoelectronic plethysmography during exercise
Perez-Tarazona, 2020, Spain (83)	13	"The present study aimed to evaluate the respiratory outcomes of "new" BPD in adolescents who were born preterm."	Multicentre cross-sectional study	286 adolescents, 184 born extremely preterm (<28 weeks) – 92 with BPD, and 102 moderate-late preterm (32 - <37 weeks)	None	Lung function testing, skin prick testing, questionnaires on asthma and QoL.
Santema, 2020, Netherlands (84)	11	"We aimed to predict adult lung function and lung density in a cohort of premature infants born in the surfactant era, representing "new BPD"."	Prospective cohort	64 preterm born adults, 36 with BPD, 28 non-BPD	No control	Lung function and lung density by CT scan
Vanhaverbeke, 2021, Belgium (85)	15	"We aimed to assess BPD using Functional respiratory imaging and to correlate these findings with the clinical presentation."	Case control study	37 children born <31 weeks gestation, 22 developed BPD	No control group	Spirometry, DLCO, CT imaging, functional respiratory imaging

© 2022 Kotecha SJ et al. *JAMA Pediatrics.*

Bui, 2022, Australia (86)	Data from authors unable to quality score	Data from authors	Data from authors	218 children born <37 weeks of which 46 29-<34 weeks and 172 34-<37 weeks gestation	1227 term born children	Spirometry pre and post bronchodilator

© 2022 Kotecha SJ et al. *JAMA Pediatrics.*

eTable 5. Demographics of the included articles

Author	Subjects (SEx)	Gestational Age (weeks)	Birth weight (grams)	Duration on mechanical ventilation	Age Tested (years)	Cohort Birth Year From	Cohort Birth Year To	Surfactant Given	Method of measuring lung function	Method of standardising lung function measurements
Aoyama	45.5% female	Mean 26.8 weeks, SD 2.9 (range 22.9 – 36.0)	Mean 980, SD 518 (range 380 – 3150)	Not given	5 – 7 years	Not given	Not given	Not given	"Testing was performed using MedGraphics Platinum Elite Series Plethysmograph at two different clinic locations and according to the American Thoracic Society (ATS) and European Respiratory Society (ERS) standards."	"Forced vital capacity (FVC) and forced expiratory volume in 1 s (FEV1) were recorded and adjusted for sex, age, height, and race per protocol. Quantitative measurements as well as global lung initiative (GLI) z-scores were recorded. Spirometry measurements were accepted if they met the acceptability and repeatability criteria of the ATS and ERS"
Arroyas	<32 weeks group 36 (48.6%) male 32-36 weeks group 38 (51.4%) male	<32 weeks group mean 28.0 (SD 1.9) 32-36 weeks group 34.2 (1.2)	<32 weeks group mean 1096 (SD 363), 32-36 weeks group 2041 (526)	No duration given, but proportion of group. <32 weeks group 48 (68.9%), 32-36 weeks 3 (4.1%)	<32 weeks group median 13.9 IQR 13.5-14.4 32-36 week group, median 14.3 IQR 14.1 – 14.5	2003	2004	<32 weeks group 41 (55.4%), 32-36 weeks group 2 (2.7%)	Jaeger Master-Scope-PC spirometer (VIASYS HealthCare GmbH, Hoechberg, Germany). At least three reproducible manoeuvres	Results were presented as percentage of predicted values with reference values of Zapletal and z-score of predicted values with

© 2022 Kotecha SJ et al. *JAMA Pediatrics.*

									were performed, selecting the one with best FEV-1 and FVC values.	reference values of the Global Lung Function Initiative (GLI)
Durlak	BPD group 8 (73%) male, No BPD group 16 (55%) male	BPD group mean 27.3 (SD 4), No BPD group 28 (5)	BPD group mean 975 (SD 284), No BPD group 1045 (268)	Not given	BPD group mean 6 (SD 1), No BPD group 7 (0), Term controls 7 (0)	2008	2010	BPD group 8 (73%), No BPD group 14 (48%)	Jaeger MasterScreen system (Carefusion Technologies, San Diego, California, USA)	Spirometry was performed with the same equipment, in accordance with the European Respiratory Society/American Thoracic Society (ERS/ ATS) criteria. The forced expiratory volume in 1 s (FEV1) and forced vital capacity (FVC) results were converted to z-scores using Global Lung Initiative (GLI) reference values
Filippo	Preterm 27/28 male/female. Term 27/28 male/female.	Preterm mean 30.6 (SD 1.6) Term mean 38.4 (SD 1.4)	Preterm mean 1430 (SD 400) Term mean 3120 (600)	Preterm group mean 1.2 days (SD 2.5). Term group none.	Preterm group mean 11 (SD 0.6). Term group 11.1 (0.9)	2006	2007	Not given	Spirometer Master Screen; Viasys GmbH—Erich Jaeger, Hoechberg, Germany. They were made in the standing position using a nose clip. Until three consecutive technically acceptable	Z-scores for DLCO, FEV1, FVC, FEF75, FEF25–75 and FEV1/FVC were derived using prediction equations from the Global Lung Function Initiative (GLI-2012)

© 2022 Kotecha SJ et al. *JAMA Pediatrics.*

									curves were achieved according to ATS/ERS guidelines. Total Lung Capacity (TLC), specific airways resistances (sRaw) and residual volume (RV) were measured using standardized body plethysmography (Vmax R Autobox V62J, Carefusion, Hoechberg, Germany).	
Hart	Preterm-born low lung function 68 (52%) male. Preterm-born controls 211 (52% male). Term-born 100 (51%) male.	Preterm-born low lung function median 31 weeks (range 24-34). Preterm-born controls median 32 weeks (range 23-34). Term-born 40 (37-42)	Preterm-born low lung function median 1450 (range 482-2930). Preterm-born controls 1758 (450-3912). Term-born 3430 (2155-4916)	Not given	Preterm-born low lung function mean 9.9 95% CI (9.7-10.2). Preterm-born controls 9.5 (9.4-9.7). Term-born 9.7 (9.5-9.8)	2005	2013	Not given	Microloop; CareFusion, Wokingham, UK	"Spirometry was quality controlled as per European Respiratory Society/American Thoracic Society guidelines including independent quality control and normalised against Global Lung Function Initiative reference values"
Joshi	CLD group 17 (59%) male, preterm group 15 (46%) male,	CLD group mean 27.3 (SD 2.1), Preterm group mean 30.0 (SD 2.0),	CLD group mean 1100 (SD 400), Preterm group mean 1500 (SD 400),	CLD group median 28 (range 5 – 80), Preterm group median 3	CLD group mean 10.2 (SD 1.4), Preterm group mean 10.3 (SD 1.1),	Not given	Not given	CLD group 29 (100%), Preterm group 15 (45%), Term group none.	An animated, computer-programmed spirometer	"Each child performed at least 3 acceptable and

© 2022 Kotecha SJ et al. *JAMA Pediatrics.*

	term group 15 (50%) male.	Term group 39.8 (SD 1.5)	Term group 3400 (SD 500)	(range 0 – 50), Term group none.	Term group 10.5 (SD 1.5)				(Flowscreen; Viasys Healthcare, Basingstoke, United Kingdom)	reproducible spirometric manoeuvres in accordance with American Thoracic Society and European Respiratory Society guidelines. Absolute values for forced vital capacity, FEV1, forced expiratory flow at 25-75% of vital capacity, and peak expiratory flow, along with percent predicted values corrected for age, sex, and height, were calculated.”
Naples	Not given	Median 27+2 weeks	Not given	Not given	Median 13.2	Not given	Not given	Not given	Standard lung function tests were performed including spirometry, body plethysmography and gas transfer testing. A symptom limited, peak exercise test was performed on a cycle ergometer,	z-scores calculated but not referenced

© 2022 Kotecha SJ et al. *JAMA Pediatrics.*

									with total and compartmental lung volumes measured using OEP.	
Perez-Tarazona	EP-BPD (n=92) 42 (45.7%) female, EP-NoBPD (n=92) 41 (446%) female, MLP (n=102) 46 (45.1%) female	EP-BPD mean 26.1 (SD 1.36), EP-NoBPD 27.0 (0.91), MLP 34.0 (1.13)	EP-BPD mean 839 (SD 188), EP-NoBPD 1004 (220), MLP 1983 (526)	EP-BPD median 15 days (IQR 4, 30), EP-NoBPD 1 (0, 3.75), MLP 0 (0, 0)	EP-BPD mean 14.1 (SD 0.71), EP-NoBPD 14.2 (0.61), MLP 14.5 (0.68)	2003	2005	EP-BPD 86 (93.5%), EP-NoBPD 61 (67%), MLP 4 (4%)	All patients underwent spirometry with bronchodilator testing.	The Global Lung Function Initiative equations were used as references for spirometry and diffusion, while equations from Rosenthal et al. were used for plethysmography. All measurements were performed according to American Thoracic Society/European Respiratory Society guidelines
Santema	Non-BPD group 18 (64%) male, BPD group 24 (67%) male	Non-BPD group mean 31.0 (SD 2.6), BPD group 28.2 (2.3)	Non-BPD group mean 1484 (SD 440), BPD group 1059 (334)	Not given	Non-BPD group mean 21 (SD 2.5), BPD group 19.7 (1.1)	1987	1988	Non-BPD group 11 (39%), BPD group 21 (58%)	Lung function was assessed according to the ATS/ERS recommendations, using Jaeger Masterscreen PFT-Pro and Jaeger Masterscreen CS functional residual capacity (FRC). About 15 min before spirometry, all	z-scores obtained by prediction equations, as recommended by Quanjer et al.

© 2022 Kotecha SJ et al. *JAMA Pediatrics.*

									participants had inhaled 400 µg salbutamol.	
Vanhaverbeke	Not given	No BPD group median 29 (range 26 – 30.6) BPD group 27.93 (24.9 – 30.3)	No BPD group median 1240 (range 637 – 1904). BPD group 973 (469 – 1640)	No BPD group median 3 (1 – 11) BPD group 9 (1 – 45)	No BPD group median 15.2 (13.5 – 16.7) BPD group 15.2 (14 – 16.9)	1999	2002	No BPD group 5 (33%). BPD group 15 (68%)	Lung function testing comprised of conventional spirometry with bronchodilator response (salbutamol), body plethysmography, nitrogen multiple-breath washout testing and a single-breath carbon monoxide diffusion test.	Not given
Hagman	37M/34F who had spirometry 28M/25F without BPD 19m 20f who had spirometry 14m15f WITH bpd 18m 14f who had spirometry 14m10f	<32 weeks MEDIAN 27 + 3 range (23+6-31+5) without BPD 27+6 (23+6-31+5) WITH bpd 26+2 (24-30)	median 960 range (550-2025) without BPD 1065 (658-2025) with BPD 828 (550-1548)	median 2 range 0-41 56% had mechanical ventilation without BPD 17 (44%) median 0 (0-28) with BPD 23 (72%) median 5 (0-41)	Median 12.6 (range 12.3-13.5) years no BPD 12.6 (12.3-13.5) BPD 12.6 (12.3-13.3)	2001	2003	40 (56%) without BPD 16 (41%) with BPD 24 (75%)	NOT STATED	"Results of spirometry and body plethysmography are reported as per cent of predicted by height and sex using reference equations from Zapletal."
Hayden	50% Male	Range 23 +3 to 36+1 weeks Mean 27 +1	Range 455-2870g Mean 915g	Not given	Mean age 7 SD (2)	1999	2013	Not given	Not given	Not given
Vrijlandt	Preterm 21/16 male/female Controls 18/16 male/female	Preterm 34 (SD +/- 1 weeks, range 32-35) Term 39 (SD +/- 0.9 weeks, range 38-41)	Preterm (2442 +/- SD 539g, range 1345-3900) Term (3693 +/- 393g, range 3070-4390)	Not given	Preterm 13.6 SD 0.6 (range 12-14) Controls 13.5 SD 0.5 (range 12-14)	Not given	Not given	Not given	Not given	Not given
Arigliani	Preterms n=25 53% boys	Preterms born <28 weeks Preterms range	Preterms <1kg BPD Median 0.7 (IQR 0.6,0.85)	BPD median 8 days (IQR 5,15) Non-BPD 2 (1,4)	8-14 years Preterms mean 11.3 SD 2 years	2004	2010	BPD n=14 (82%) non-BPD 24 (80%)	"N2 MBW (Exhalyzer D; Eco Medics AG,	"Spirometry and TLco results were converted

© 2022 Kotecha SJ et al. *JAMA Pediatrics.*

	Controls n=30 50% boys	23-27 BPD median 27 (IQR 25.2, 27.2) non-BPD 26.1 (25.2, 27.0)	Non-BPD 0.88 (IQR 0.77,0.94) Controls no data		Controls mean 11.6 SD 1.9 years				Switzerland, running Spiroware Version 3.1.6 software), spirometry, and TLco (Jaeger MasterScreen PFT; Viasys Healthcare, Germany) were performed in this order on the same day, according to European Respiratory Society/American Thoracic Society (ERS/ATS) standards adapted for children."	to z-scores according to the Global Lung Initiative (GLI) reference values"
Arroyas Sanchez	55% female	Average weeks 28-30 (25-32)	Weight 870g (518-1450)	During resuscitation, comparing BPD vs non-BPD 56.6% ventilated vs 22.4% Respiratory assistance during admission: mechanical ventilation (mild 59,7 h, moderate 210h, severe 806 h), oxygen (mild 919 h, moderate 1279 h, severe 2549 h).	Tested by 5 years of age	2005	2008	First 24 hr admission Comparing BPD vs non-BPD 80% vs 40.4%	Not given	Not given

© 2022 Kotecha SJ et al. *JAMA Pediatrics.*

Bar-Yoseph	Boys p- 9 (60%) p +18 (67%)	29-32 weeks gestation p- (mean SD) 31.09 ± 0.59 p+ 30.3 ± 0.91	p- (mean SD) 1540.3 ± 243.6 p+ 1329.8 ± 228.6	Excluded babies mechanically ventilated for >24 hours	5-7 years Age (yrs) mean (SD) p- (mean SD) 5.7 ± 0.5 p+ 5.7 ± 0.86	2007	2010	P- 1/15 (7%) P+ 8/27 (29%)	"Spirometry: was performed according to the American Thoracic Society (ATS) and European Respiratory Society (ERS) guidelines for preschool children using a KoKo spirometer (nSpire Healthcare Inc.,Longmont, CO). Force expiratory volume (FEV) curves were measured using the program incentive targets. Tests were performed with participants standing with a nasal clip. Baseline maneuvers were repeated until achievement of the best possible visual effort on at least three technically acceptable FEV curves."	Not given
Bozzetto	groups were sex matched but gender split not stated	BPD median 29 weeks interquartile range 27-30	BPD median 1020g IQR 855-1190g	BPD median 20 days of mechanical ventilation at	11 to 19 years BPD median 14.3 years other groups	Not given	Not given	17 of the BPD group had surfactant	"Spirometry was performed according to the American	Quanjer PH, Stanojevic S, Cole TJ, Baur X, Hall GL, Culver

© 2022 Kotecha SJ et al. *JAMA Pediatrics.*

				birth IQR 9.5-31.7	were age matched				Thoracic Society/European Respiratory Society (ATS/ERS) standards"	BH, Enright PL, Hankinson JL, Ip MS, Zheng J et al. Multi-ethnic reference values for spirometry for the 3-95-yr age range: the 4 (2016) Asian Pacific Society of Respirology 1116 S Bozzetto et al. Respirology (2016) 21, 1113-1117 © 2016 Asian Pacifi c Society of Respirology global lung function 2012 equations. Eur. Respir. J. 2012; 40: 1324-43.
Cardoen	13 FEMALES, 11 MALES	median 29 IQR 26.9, 31.3	Median 1227 IQR 900,1647 G	Assisted ventilation days 31 IQR 19,38	median age first PFT 7.6 years (6.3,8.4) last 18.2 years (15.5, 26.3) mean 7.79 (1.98 and 19.90 (5.68)	2001	2011	Not given	All lung function tests had been performed on a Jaeger spirometer following ATS guidelines.	Lung function tests were reviewed, and results were expressed as percent predicted (pp or % pred) according to the GLI equations
Cazzato	Male/Female All VLBW: 27/21 - No-BPD: 15/11 - BPD: 12/10 Control: 25/21	Mean (range) All VLBW: 28 (24-32) - No BPD: 30 (28-32) - BPD: 26 (24-30) *p <0.001 No-BPD vs BPD	Mean +/- SD All VLBW: 1050 +/- 278 - No BPD: 1220 +/- 230 - BPD: 840 +/- 160 *p <0.001 No-BPD vs BPD	Day's median (IQR) All VLBW: 0 (3) - No BPD: 0 (0) - BPD: 2 (11) *p <0.001 No-BPD vs BPD	Mean +/- SD All VLBW: 8.5 +/- 1.0 - No BPD: 8.4 +/- 1.2 - BPD: 8.7 +/- 0.8 Control: 8.8 +/- 1.4	1996	1999	% All VLBW: 56% - No BPD: 20% - BPD: 94% *p <0.001 No-BPD vs BPD	Performed in accordance to ATS/ERS guidelines, with investigator masked to birth status	Lung function values (except RV/TLC ratio) were expressed as z-scores to adjust for height, sex, and age based on Stanojevic 2008

© 2022 Kotecha SJ et al. *JAMA Pediatrics.*

										and Rosenthal 1993
Chang	Preterms 46% male terms 59%	preterm mean gestation 28.6 SD 2.7 terms mean 38.5 SD 1.0	preterm mean 1,039 g SD 247g TERMS 3030g SD 322	preterms 62% ventilated mean duration 17.6 SD 27.4	5-6 years Preterms mean 5.6 SD 0.6 Terms mean 5.9 SD 0.5	2015	2017	Preterms 40% given	"Spirometry was performed by a single experienced technician, and data were analyzed by respiratory specialists who were all blinded to the clinical details of the participant. Spirometry was performed using a spirometer (Ultima PF with RTD; MGC Diagnostics, Saint Paul, MN, USA). Measurements were performed according to the American Thoracic Society and European Respiratory Society guidelines (23). During spirometry, attempts were made to achieve at least three technically acceptable sequences. The best maneuvers among those	"Lung function data were expressed as z-scores after adjustment for height, sex, age, and race according to the Global Lung Function Initiative reference. Any z-scores of lung function measurements <-1.96 were considered abnormal."

© 2022 Kotecha SJ et al. *JAMA Pediatrics.*

									considered technically acceptable were recorded. Flow volume curves were obtained in order to determine the following spirometric parameters: forced expiratory volume in one second (FEV1), forced vital capacity (FVC), FEV1/FVC ratio, and forced expiratory flow between 25 and 75% of expired FVC (FEF25–75)."	
Choukroun	n total (ratio M:F) All: 151 (1.5:1) Without BPD: 96 (1.5:1) Mild BPD: 41 (1.3:1) Moderate and severe BPD: 14 (3.7:1)	Median [min - max] - Weeks All: 31 [25 - 32] Without BPD: 31 [26 - 32] Mild BPD: 29 [26 - 32] Mod/severe BPD: 28 [25 - 30]	Median [min - max] - grams All: 1330 [570 - 2510] Without BPD: 1455 [700 - 2510] Mild BPD: 1250 [740 - 1820] Mod/severe BPD: 845 [570 - 1120]	Measured but not reported by group 103/151 (68.2%) of whole study group received mechanical ventilation	Mean (SD) - Years All: 8.6 (0.8) Without BPD: 8.7 (0.8) Mild BPD: 8.5 (0.8) Mod/severe BPD: 8.6 (0.8)	1997	2001	Measured but not reported by group 70/151 (46.4%) of whole group received surfactant	Spirometry performed in accordance with ERS/ATS criteria. DLCO was measured using the single breath method (Hyp'air Medisoft Dinant Belgium). After baseline lung function was measured, exercise - induced bronchial reactivity was assessed using a cycle	Spirometry: Quanjer 1989 (% and z) DLCO: Routabi 2006 (%)

© 2022 Kotecha SJ et al. *JAMA Pediatrics.*

									ergometer (Ergoline, GmbH& Co.KG, Germany). (See Supplement 1)	
Cristea	Female: 10 (52.6%)	Mean (95% CI) - weeks 27.5 (26.2; 28.8)	Median (IQR) - grams 795 (737.5; 1179)	Mean (95% CI) - Years 2.44 (2.1; 2.8)	Median (IQR) - Age of first spirometry 6.6 (4.9; 8.3) Full age range figures not given but based on figures between ~5 years and 25 years	1984	2010	Not given	Conventional spirometry was performed when the patient was weaned from ventilation, decannulated, and able to follow commands. Testing was done standing and with a nose clip. All the tests were performed using special incentive software. Not all subjects could complete all respiratory function tests because of poor cooperation. Only those values that met the ATS/ERS acceptability criteria were considered.	Stanojevic 2008
Crowther	% (n) Male Treatment: 56 (275) Placebo: 54 (268)	Mean (SD) - weeks Treatment: 32.5 (3.6) Control: 32.4 (3.6)	Mean (SD) - grams Treatment: 1917 (831) Control: 1910 (808)	Not given	Mean (SD) - corrected age in years Treatment: 7.7 (1.1) Control: 7.8 (1.2)	1998	2004	Not given	Lung function was measured by portable flow spirometry (EasyOne 2001; NDD Technologies, Zurich, Switzerland) by	Z-scores - Stanojevic 2008

© 2022 Kotecha SJ et al. *JAMA Pediatrics.*

									using forced expiratory maneuvers. Spirogram quality was determined by 2 independent examiners, and children with at least 2 adequate and reproducible spirograms were included in the analysis	
De Sousa Sena	not stated	mean 27.9 (3.6) weeks	mean 1.12 (0.5) kg	Not given	mean age 22.5 (1.6) years	1993	1997	Not given	Not given	Not given
Debevec	All male subjects	Mean (±SD) 29 ± 4 weeks	1264 ± 297 g	Not given	21 ± 1	Not given	Not given	Not given	The lung function testing was performed using the pneumotachograph (Cardiovit AT-2plus, Schiller, Baar, Switzerland) in line with the established criteria (Miller et al., 2005). The device was calibrated prior to each test using a 3-L syringe. Each test was performed three consecutive times and the highest of the three values of forced vital capacity (FVC) and forced expiratory	The percentage predicted FVC and FEV1 values were calculated based on the GLI ERS Task Force equations (Quanjer et al., 2012).

© 2022 Kotecha SJ et al. *JAMA Pediatrics.*

									volume in 1 s (FEV1) were recorded and subsequently used to calculate the FEV1/FVC ratio.	
Devakumar	Term 381 G 403 B Preterm 25 G 32B	If preterm, mean gestational age = 35.1 weeks (SD 2.1) If term, mean gestational age = 39.7 weeks (SD 1.4)	Not given for preterm and term groups	Not given	Average age at time of testing = 8.46 years Average age if term = 8.45 years Average age if preterm = 8.52 years	2002	2004	Not given	"Lung function was measured using two identical EasyOne World Spirometers (ndd Medical, Zurich, Switzerland), auto-calibrated before use and alternated fortnightly. American Thoracic Society/European Respiratory Society quality control criteria for spirometry, adapted for use in children, were used. Parents were requested to bring their children for assessment only if they were well. Three local investigators were trained to conduct spirometry tests. They explained and demonstrated the procedure	"Lung function data were adjusted for sex, age and height using the Global Lung Function Initiative (GLI)-2012 multi-ethnic, "all-age" reference ranges "

© 2022 Kotecha SJ et al. *JAMA Pediatrics.*

									to the child and parent/guardian in advance. The child performed spirometry wearing a nose clip while seated. A biological control (member of staff) was tested every fortnight to monitor any potential shift in both spirometers over time. All spirographs were interpreted by a clinician (D. Devakumar) and one in 10 were over-read by a respiratory physiologist (J. Kirkby), who had provided initial spirometry training. "	
Doyle	note: data extracted from supplementary data which gives values based on those who performed lung function, not main paper which also includes those in cohort who	mean (SD) 1997: 25.6 (1.2) 2005: 25.8 (1.2)	mean (SD) 1997: 822 (176) 2005: 869 (190)	Not given	mean (SD) 1997: 8.4 (0.3) 2005: 7.7 (0.4)	1991	1992	n (%) 1997: 97 (87%) 2005: 104 (85%)	Spirometry was performed in accordance with guidelines from the American Thoracic Society by respiratory scientists who were unaware of the clinical	z-scores (GLI 2012) - Quanjer 2012

© 2022 Kotecha SJ et al. *JAMA Pediatrics.*

	could not do lung function no. male (no. total) 1997: 59 (112) 2005: 56 (123)								de-tails regarding the participants.	
Doyle	n. total (n. male) Caffeine: 74 (31) Control: 68 (41)	mean (SD) Caffeine: 27.7 (1.7) Control: 27.3 (1.8)	mean (SD) Caffeine: 967 (182) Control: 926 (181)	Not given	mean (SD) Caffeine: 11.4 (0.4) Control: 11.4 (0.6)	1999	2004	n (%) Caffeine: 42 (57%) Control: 48 (71%)	According to ATS/ERS standards.	Spirometry - Quanjar 2012 (GLI)
Flahault	n (%) / Total Term: 43 (41%) / 105 Preterm: 45 (45%) / 101	Median (IQR) Term: 40.0 (39.0 to 40.3) Preterm: 27.4 (26.1 to 28.2)	Median (IQR) Term: 3400 (3150 to 3670) Preterm: 920 (800 to 1130)	Not given	Median (IQR) Term: 23.1 (21.3 to 24.8) Preterm: 23 (21.2 to 24.8)	1987	1997	n (%) 41 (41%) / 101	Not given	Not given
Fortuna	BPD 13/15 F/M EP No BPD 12/8 F/M Controls 12/15 F/M	BPD mean 25.5 SD 1.4 EP no BPD 26.4 (1.1) Controls 39.5 (1.1)	BPD mean 716 (143) EP no BPD 850 (147) Terms 3278 (387)	BPD mean 35.9 SD 20 EP no BPD 6.8 (10)	Lung function tested at 8 and 12 years of age. Just data for 8 years used in systematic review BPD mean 8.2 (1.1) and 11.6 (1.3) EP no BPD 8.1 (1.3) and 11.8 (1.3) Controls 8.3 (1.5) and 11.7 (1.6)	1999	2002	BPD 25/28 (86%) EP no BPD 10/20 (50%)	" Spirometry was performed using a 10-L bell spirometer (Biomedin, Padova, Italy) according to international recommendations. The same model of spirometer was used at both assessments. The spirometer's calibration was checked weekly with a 3-L syringe (Biomedin, Padova, Italy). Children were asked not to take short-acting bronchodilators for at least a day before assessment."	"FVC, FEV1, FEF25-75% were measured and expressed as z-scores and percent predicted values, calculated using the Global Lung Function Initiative 2012 Equations."

Gaffin,	preterm group 28/47 male term group 128/51	Preterm <37 weeks term	Not given	Not given	preterm mean 7.4 SD 1.9 TERM MEAN 8.0 SD 1.9	2008	2013	Not given	Not given	Not given
Goncalves	n (%) 44 (52.4%) males	31.8 +/- 2.4	1423 +/- 49	4 +/- 10 days	9.3 +/- 2.3	2008	2011	Not given	Current standardised pulmonary function testing was conducted according to the 1st Brazilian Consensus on Spirometry and ATS/ERS TaskForce 2005 with Pony FX spirometer, Cosmed, after daily calibration. Each patient completed at least threere producible flow volume curves before and 15 min after the inhalation of salbutamol400ug (Flumax aerosol spacer).	The spirometric values were analysed in relation to the percentage of predicted values by Polgar and Promadhat. Bronchodilator response was calculated as a percentage of increase in relation to the predicted FEV1 value as described by Pellegrino et al.
Gough	Male: # (%) BPD: 31 (55) non-BPD: 15 (38%) Term: 27 (49%)	Mean (SD) BPD: 27.1 (2.1) non-BPD: 31.2 (2.3) Term: 39.8 (1.2)	Mean (SD) BPD: 939 (246) non-BPD: 1234 (223) Term: 3556 (429)	Hours, Median [IQR] BPD: 783 (425.8-1478.8)	Mean (SD) BPD: 24.1 (4.0) non-BPD:25.3 (4.0) Term: 25.8 (3.9)	1978	1993	Mean (SD) BPD: 18 (32)	Lung function tests were performed using a portable MicroLab ML3500 Mk8 spirometer (Micro Medical Ltd, Basingstoke, UK) according to European Respiratory Society lung	Percent predicted and z-score

© 2022 Kotecha SJ et al. *JAMA Pediatrics.*

									function testing guidelines	
Greenough,	not stated	median gestation 27 range 22-31 weeks home O2 median 27 (22-31) range no home O2 27 (24-31) 28 home O2 had LF 28 (24-31) 56 no home O2 had LF 27 (24-31)	Home O2 median 993 (510-1,826) No home O2 median 1,003 (515-1,960) 28 home O2 had LF 1015 (650-1826) 56 no home O2 977 (514-1520)	duration not given rates given IPPV home O2 95% no home O2 97% those that had LF home O2 100% no home O2 96%	Home O2 median 10 (9-12) No home O2 10 (8-12)	1994	1997	home O2 91% No home O2 79% Those that had LF home O2 100% no home O2 73%	"No child was tested within 2 weeks of an upper respiratory tract infection. Short-acting bronchodilators were withheld from midnight on the previous day, if possible, but always for at least 4h before testing. At baseline, forced expiratory volume in 0.75 s, forced vital capacity, forced expiratory flow (measured from the top of the breath such that FEF75 is the flow at 75% FEF50 is the flow at 50% and FEF25 at 25% of the vital capacity) and peak expiratory flow were measured using a Lilley pneumotachograph (Vitalograph 2120, Vitalograph, Buckingham, UK)"	"The lung function results were expressed as percentage predicted for height"

© 2022 Kotecha SJ et al. *JAMA Pediatrics.*

Greenough.	CV 85/159 male HFO 77/160 male	CV mean 27 SD 1.18 HFO mean 26.7 SD 1.45	CV mean 923 SD 206 HFO mean 867 SD 209	Not given	mean (SD) [range] CV: 12.5 (0.6) [11.2-14.4] HFO: 12.6 (0.62) [11.5-14.4]	1998	2001	CV 158/159 HFOV 152/160	"Airway function was assessed by spirometry FEF75, FEF50 or FEF25, FEV1, PEF. A minimum of three flow-volume loops with results 10% of each other were recorded, and the flow-volume loop with the highest FEV1 analysed. Assessment was also made by impulse oscillometry. In addition, inhomogeneity of ventilation distribution was assessed by a multiple breath technique including the lung clearance index (LCI). Plethysmographic assessment of FRCpleth, total lung capacity (TLC) and residual volume (RV) were made. Measurements were made at least twice and mean values within 10% of	"All lung function results were standardised for sex and height using the reference ranges of Rosenthal et al.36,37 and Nowowiejska et al.38"

© 2022 Kotecha SJ et al. *JAMA Pediatrics.*

									each other were recorded. Total lung gas transfer, alveolar volume (VA) and gas transfer per unit volume were assessed using the single breath gas transfer technique. All lung function results were standardised for sex and height using the reference ranges of Rosenthal et al and Nowowiejska et al. Airway hyperreactivity was assessed by a bronchial challenge tailored to the child's baseline lung function. Children with a baseline FEV1≤ 70% of predicted received a bronchodilator and their FEV1 and FRC were remeasured. Children with a FEV1> 70% of that predicted underwent a cold-air challenge. FEV1	

									was measured prior to, and then every, 2 minutes for 12 minutes after the cold-air challenge had finished.”	
Hadchouel	not given “more girls in control subjects"	study group 22-32 weeks gestation controls term	Not given	Not given	15 years	1997	1997	Not given	"LFT were performed according to the recommendations of the American Thoracic Society/European Respiratory Society task force. FEV1 measures were expressed as Z-score values according to GLI 2012 lung function regression equations (www.lungfunction.org). Forced vital capacity (FVC), slow vital capacity (SVC), FEV1, functional residual capacity by plethysmography (FRC), total lung capacity (TLC), residual volume (RV), forced expiratory flow 50 (FEF50),	"LFT were performed according to the recommendations of the American Thoracic Society/European Respiratory Society task force. FEV1 measures were expressed as Z-score values according to GLI 2012 lung function regression equations (www.lungfunction.org). Forced vital capacity (FVC), slow vital capacity (SVC), FEV1, functional residual capacity by plethysmography (FRC), total lung capacity (TLC), residual volume (RV), forced expiratory flow 50 (FEF50),

© 2022 Kotecha SJ et al. *JAMA Pediatrics.*

									and forced expiratory flow 25-75 (FEF25-75) were measured, before and 15 minutes after inhalation of 400µg salbutamol. A positive response to bronchodilators was defined by an increase in FEV1 > 12% after inhalation of salbutamol. An obstructive ventilatory defect was defined by a FEV1 Z-score < -1.64. FeNO and DLCO were also measured"	and forced expiratory flow 25-75 (FEF25-75) were measured, before and 15 minutes after inhalation of 400µg salbutamol. A positive response to bronchodilators was defined by an increase in FEV1 > 12% after inhalation of salbutamol. An obstructive ventilatory defect was defined by a FEV1 Z-score < -1.64. FeNO and DLCO were also measured"
Hamon	Preterm 17 m 25 f controls 9m 18f	Preterms born <32 weeks gestation mean 28.9 SD 1.8 controls mean 39.7 SD 1.2	Preterms mean 1.16KG SD 0.32 controls 3.26kg SD 0.42	Preterms (41/42) median 28 days range 1-102	7 years preterms mean 7.1 years SD 0.4 controls 7.0 years SD 0.6	1999	2001	Not given	Forced spirometry was performed using an electronic flowmeter with computer animation programs (Masterscope, Erich Jaeger, Wuertzburg, Germany).	Baseline FVC, FEV1, and the ratio of FEV1 to FVC were expressed as z-scores using recently published algorithms
Hayden	55% male	22 6/7 to 36 4/7 weeks mean 27 1/7 weeks	370-3100g mean 909g	Not given	mean 5.4 years SD 2.3	1999	2013	Not given	Not given	Not given

© 2022 Kotecha SJ et al. *JAMA Pediatrics.*

Hirata	81/201 Male 40.3%	median 26.6 weeks (range 22.7-34.6)	median 756g (range 390-1000)	Ventilation discontinued median 34 weeks (Range 27-118)	8 years median 8.02 range 7.02-9.25 years	1990	2004	135/201	"Portable spirometers (1999-2010: AS-502, Minato Medical Science, Osaka, Japan; 2011: ARFEL III, AiVision, Tokyo, Japan). FVC, FEV1 and forced expiratory flow at 50% and 75% were obtained from maximum expiratory flow-volume curves. During spirometry, attempts were made to achieve at least three acceptable and two repeatable forced expiratory manoeuvres. Results of spirometric data are expressed as per cent predicted for age, height and sex relative to Japanese children."	"Results of spirometric data are expressed as per cent predicted for age, height and sex relative to Japanese children."
Kaczmarczyk	All female, no BPD	Mean 34.7 weeks SD 1.86 range 32-36 for the 70 tested and mean 34.5 weeks SD 1.92 range 32-36 for	Preterms <2.5kg terms >/=2.5kg	Not given	Examination one mean 12.5 years SD 1.52 range 10-14 years examination 2 mean 13.5 SD	1983	1987	Not given	"All measurements were taken in accordance with the guidelines of the European	"Data on race, sex, age, and height of the participant were entered into the ECSC (European Coal

© 2022 Kotecha SJ et al. *JAMA Pediatrics.*

		the 12 tested in adulthood controls were term (>37 weeks, >2.5kg birth weight) for the adult group			1.52 range 11-15 then spirometry 17 years later in 12 children aged 28.08 SD 2.43 range 23-29 controls mean 28.32 SD 2.13 range 23-32 control adult group 28.3 SD 2.16				Respiratory Society and American Thoracic Society. For the first and second sessions, spirometric measurements were taken using a Lungtest 1000/MES spirometer (MES LLC, Cracow, Poland), whereas for the third a portable Lungtest Handy (MES LLC, Cracow, Poland) was used. The first and second examinations recorded the following parameters: VC (vital capacity), FEV1 (forced expiratory volume in 1 s) and MVV (maximal voluntary ventilation). The third recorded the parameters: VC, FEV1, FVC (forced vital capacity), MMEF (maximal mid-expiratory flow) and forced	and Steel Community) calculator (for 1997 and 1998 data) or GLI 2012 (Global Lung Function Initiative, Berlin, Germany) calculator (for 2015 data), yielding a percentage of the norm for healthy children."

© 2022 Kotecha SJ et al. *JAMA Pediatrics.*

									expiratory flow at 25-75% of forced vital capacity (FEF25-75), MEF75 (maximal expiratory flow at 75% of forced vital capacity), MEF50 (maximal expiratory flow at 50% of forced vital capacity), MEF25 (maximal expiratory flow at 25% of forced vital capacity). FEV1, FVC, FEF25-75, MEF25 and data on race, sex, age, and height of the participant were entered into the ECSC (European Coal and Steel Community) calculator (for 1997 and 1998 data) or GLI 2012 (Global Lung Function Initiative, Berlin, Germany) calculator (for 2015 data).	
Karnaushkina,	in total 17 men and 8 women	preterms mean 32.8 SD 1.2 LRTI	preterms mean 1910 SD 484.1	Preterms were ventilated, not	preterms mean 21.1 SD 2.8 LRTI	Not given	Not given	Not given	Respiratory functional	Respiratory functional

© 2022 Kotecha SJ et al. *JAMA Pediatrics.*

		39.5 SD 1.0 Healthy 39.6 SD 1.1	LRTI 3583.3 SD 511.5 Healthy 3600.0 SD 405.3	stated how many	22.8 SD 1.5 Healthy 21.2 SD 2.2				diagnosis was carried out according to the standard protocol of the European Coal and Steel Community using MasterScreen Body equipment (Erich Jaeger, Germany). The data obtained were compared with the reference values calculated with the help of the formulas given in the mentioned protocol. Organs of the thoracic cavity of all patients were examined using multispiral computed tomography (MSCT) with functional tests conducted by means of Somatom Sensation 40 CT Scanner (Siemens, Germany)."	diagnosis was carried out according to the standard protocol of the European Coal and Steel Community using MasterScreen Body equipment (Erich Jaeger, Germany). The data obtained were compared with the reference values calculated with the help of the formulas given in the mentioned protocol. Organs of the thoracic cavity of all patients were examined using multispiral computed tomography (MSCT) with functional tests conducted by means of Somatom Sensation 40 CT Scanner (Siemens, Germany)."
Kilbride,	Not given	ELBW mean 24.7 SD 1.3 heavier	ELBW 674g SD 83g Heavier preterms mean 1952g SD 278	Not given	12-15 years	1993	1995	Not given	not stated	Not given

© 2022 Kotecha SJ et al. *JAMA Pediatrics.*

		preterms mean 33.2 SD 1.7								
Kilbride,	NO group 8/18 (44%) male placebo group 11/16 (69%) male	NO group 25.6 +/- 1.5 weeks placebo group 25.3 +/- 1.5 weeks	NO group 766 +/- 179g placebo group 731 +/- 145g	NO group 30 +/- 16 days placebo group 42 +/- 21 days	7-9 years mean 9.5 +/- 1.5 years NO group 9.1 +/- 1.5 years placebo group 9.9 +/- 1.4 years	2000	2005	Not given	"Spirometry was performed, including forced vital capacity (FVC), forced expiration volume at 1 second (FEV1), FEV1/FVC, mid expiratory flows (25-75%), and peak flow rates, determined as percentage of predicted levels for gender/age. Children with FEV1 < 80% were restudied after inhaled bronchodilator (albuterol 90 mcg/puff, 4 puffs). Children with reactive bronchospasm were referred for treatment. All other children proceeded to exercise testing."	percent predicted; method not given
Konefal	Preterm BPD male 12/20 (60%) preterm no BPD male 19/38 (50%) terms male 45/90 (50%)	Preterm BPD mean 29.2 SD 3.12 preterm no BPD mean 34.3 SD 3.3 terms mean 39.2 SD 1.36	preterm BPD mean 1291 SD 598 preterm no BPD mean 2184 SD 788 terms mean 3371.6 SD 310	All preterms were ventilated	preterm BPD mean 10.25 SD 2.7 preterm no BPD mean 9.22 SD 2.8 terms mean 10.6 SD 2.9	1995	1999	Preterm BPD 9/20 (45%) preterm no BPD 6/38 (15.8%) terms male 0/90 (0%)	"The pulmonary function was assessed by spirometry (Lungtest 500; MES, Cracow, Poland). The pulmonary function testing and its duration were compliant	"Standard reference equations (variables included gender, height, and weight) were used for all lung parameters in order to obtain

© 2022 Kotecha SJ et al. *JAMA Pediatrics.*

									with the standards recommended by the American Thoracic Society. The results were calculated from three comparable values of parameters that did not differ by more than 5% (or 0.1 liter) and were determined from the best of three forced vital capacity (FVC) results. "	the percentage of predicted normal values. Results were expressed as percentage predicted, with generally accepted reference ranges of 80% to 120%."
Kotecha,	# (% male) Had spirometry at 8-9 and/or 14-17 25-32w: 48 (62%) 33-34w: 54 (61%) 35-36w: 144 (54%) Term: 3446 (49%)	Mean (SD) - weeks Had spirometry at 8-9 or 14-17 25-32w: 29.7 (2.0) 33-34w: 33.6 (0.5) 35-36w: 35.7 (0.5) Term: 39.7 (1.3)	Mean (SD) - grams / z score Had spirometry at 8-9 or 14-17 25-32w: 1437 (439.5) / 0.051 (1.297) 33-34w: 2449 (416.8) / 0.396 (1.267) 35-36w: 2634 (424.7) / 0.180 (1.066) Term: 3478 (476.6) / 0.117 (0.976)	Mean (range) - hours Had spirometry at 8-9 or 14-17 25-32w: 245 (1-1416) 33-34w: 77 (9-168) 35-36w: 15 (8-22) Term: 107 (1 - 576)	Mean (SD) - years Spirometry at 8-9 25-32w (n=68): 8.7 (0.4) 33-34w (n=81): 8.6 (0.3) 35-36w (n=248): 8.7 (0.3) Term (n=6308): 8.6 (0.3) Spirometry at 14-17 25-32w (n=43): 15.5 (0.2) 33-34w (n=49): 15.5 (0.4) 35-36w (n=132): 15.5 (0.3) Term (n=4284): 15.5 (0.3)	1990	1992	n/N (%) Had spirometry at 8-9 or 14-17 25-32w: 35/78 (44.9) 33-34w: 6/88 (6.8) 35-36w: 0/269 (0) Term: 1/6979 (0.01) Spirometry at 14-17 25-32w (n=43): 33-34w (n=49): 35-36w (n=132): Term (n=4284):	Spirometry performed at 8-9 years and 14-17 years of age. FEV1, FVC, FEV27-75 recorded.	Chinn et al ALSPAC cohort
Yen-Ping Kung,	Term 78/157 female preterm 5/8	Preterm mean 33.6 SD 2.8	Preterm mean 2167.5 SD 609.7 terms	Not given	8 YEARS	2004	2005	Not given	"We used the Micro Medical MicroLab	Not given

© 2022 Kotecha SJ et al. *JAMA Pediatrics.*

		terms 39.0 SD 1.1	3275.8 SD 412.2						Spirometer (CareFusion 232 Ltd.), complying with the American Thoracic Society spirometry standards (Miller et al., 2005), to conduct lung function tests for these eight-year-old children. After height and weight were measured, these children underwent lung function assessments by the same trained technician. Lung function values assessed were forced expiratory volume in 1 s (FEV1), forced vital capacity (FVC), peak expiratory flow (PEF), and forced expiratory volume in 1 s as a proportion of the forced vital capacity (FEV1/FVC). "	
Kwinta	ELBW 52/81 64% female	ELBW <30 weeks gestation means 27.2 SD	ELBW mean birthweight 845g SD 130G	Not given	6-7 years mean age 6.7 years SD 0.4	2002	2004	Not given	" Spirometry was performed using a Lungtest	"Results are presented as percent of

© 2022 Kotecha SJ et al. *JAMA Pediatrics.*

	terms 21/40 (53%) female	2.1 Terms 39.9 SD 1.4	(all <1000g) Terms mean 3554 SD 512 g						1000 spirometer with a pneumotachometer-based system (MES, Kraków, Poland). The forced vital capacity (FVC) forced expiratory volume in one second (FEV1), ratio of FEV1/ FVC and the forced expiratory flow at 50% of FVC (FEF50) was measured before and after the inhalation of 400 ug of salbutamol, according to the recommendations of ATS/ERS. "	predicted values with reference values of Zapletal et al. "
Lasry,	31 (70.5%) male	Mean 26.4 (SD 2.7)	Mean 792.8 (SD 175.1) n=19	26 (59.1%) intubated	Mean 19 (SD 4.7)	1980	2000	13 (29.5%)	Pulmonary function was assessed by patients' most recent spirometry measurements	Not given.
Landry,	BPD 11 Male (35%) RDS 18 (58) preterm 6 (23) term 11 (31)	Days mean (SD) BPD 192 (18) RDS 224 (25) preterm 230 (23) term 277 (10)	kg mean (SD) BPD 1.06 (0.37) RDS 1.96 (0.78) preterm 2.17 (0.80) term 3.45 (0.42)	Not given	21 or 22 years of age mean (SD) BPD 22 (2) RDS 21 (2) preterm 22 (2) term 22 (2)	1987	1993	Not given	"Whole body plethysmography, slow vital capacity, spirometry, diffusion capacity of the lung for carbon monoxide and	"We used the European Community of Coal and Steel (ECCS) predicted set of values. Predicted lung volumes were

© 2022 Kotecha SJ et al. *JAMA Pediatrics.*

									airway resistance were determined in accordance with the American Thoracic Society (ATS) guidelines in a BodyBox 5500 (Medisoft, Sorrines, Belgium) Use of short acting B agonists were withheld on the day of the test. FVC, FEV1, FEV1/FVC FEF25-75, IC FRC, TLC, VC, RV, RV/TLC, DLCO and Raw were recorded and considered acceptable if they followed the acceptability and reproducibility criteria of the ATS."	adjusted for ethnicity other than white (10% diminution)"
Lista	# males / total VG: 7/13 HFOV: 5/12	Mean ± SD VG: 27 ± 2 HFOV: 27 ± 2	Mean ± SD VG: 1086 ± 158 HFOV: 1090 ± 139	Days: Mean ± SD VG: 9.8 ± 3 HFOV: 9.6 ± 4	7 years (Range 6.5 to 7.3y)	Not given	Not given	# Doses VG: 2 ± 1 HFOV: 2 ± 1	Lung function measured by whole body plethysmograp hy (Masters Screen TM, Vyasis). Spirometry was done after setting a nose clip to close the nares and the children	"Forced vital capacity (FVC), forced expiratory volume at 1 s (FEV1), the ratio FEV1/FVC (FEV%), total lung capacity (TLC), airway resistance (RAW) and residual volume

© 2022 Kotecha SJ et al. *JAMA Pediatrics.*

									performed the exhalation maintaining a sitting position. At least three acceptable flow-volume curves were obtained according to the reproducibility criteria of the European Respiratory Society	(RV) were recorded and calibrated according to age, sex, height and weight." No mention of specific equations used
Luo,	n (%) 27 (49) males	Weeks - median (25th - 75th centile) 28.0 (26.0 - 29.4)	grams - median (25th - 75th centile) 900 (800 - 1168)	Not given	4 years (Nil further detail found)	2003	2004	Not given	QRS SpiroCard PC Spirometer equipped with incentive animation (QRS Diagnostic, LLC, Totowa, NJ). A spirometric attempt was considered acceptable by the examiner when it met the criteria of the Thoracic Society/European Respiratory Society Statement: Pulmonary Function Testing in Preschool Children	GLI z-scores
MacBean,	25M 26F No LRTI 10M 11F RV LRTI 5M 9F RSV LRTI 4M 6F Other viral LRTI 6M	median 33 + 6 weeks IQR (30+5-34+6) No LRTI 34 (33-35) RV LRTI 34 (28-35) RSV LRTI 34	median (IQR) 1866 (1192-2310) No LRTI 2260 (1576-2620) RV LRTI 1913 (1113-	Days ventilated median (IQR) 0 (0-4) No LRTI 0 (0-1) RV LRTI 2 (0-66) RSV LRTI 0 (0-9) Other	5 to 7 years median (IQR) No LRTI 6.56 (6.30-7.25) RV LRTI 7.2 (7.08-7.41) RSV LRTI	2008	2009	Not given	"All pulmonary function testing was performed by the same operator. Spirometry was	"Expressed as standardised residuals (z-scores) relative to published predicted

© 2022 Kotecha SJ et al. *JAMA Pediatrics.*

		(29-35) other viral LRTI 33 (28-34)	2153) RSV LRTI 1558 (751-1911) other viral LRTI 1984 (1250-2461)	viral LRTI 0 (1-23)	6.12 (5.95-7.02) other viral LRTI 6.83 (6.68-7.10)				conducted in accordance with ATS/ERS criteria using a Jaeger masterscreen device. Forced vital capacity (FVC), forced expiratory volume in one second (FEV1) and FEV1/FVC ratio were measured and expressed as standardized residuals (z-scores) relative to published predicted values taking into account sex, age and height"	values taking into account sex, age and height" GLI equations
Maclean,	# Participants (# male, % male) BPD (mod/severe): 47 (27, 57%) BPD (no/mild): 53 (34, 64%) Control: 64 (37, 58%)	Mean (SD) BPD (mod/severe): 26.0 (1.9) BPD (no/mild): 26.7 (1.1)	Mean (SD) BPD (mod/severe): 813 (191) BPD (no/mild): 982 (163)	Mean (SD) BPD (mod/severe): 29 (22) BPD (no/mild): 6 (11)	Mean (SD) BPD (mod/severe): 11.9 (1.6) BPD (no/mild): 12.3 (1.5) Control: 11.6 (1.9)	1997	2004	Mean (SD) - Doses of surfactant BPD (mod/severe): 2.3 (2.1) BPD (no/mild): 1.1 (1.8)	Performed according to published criteria using mass flow sensor and phlethysmography (Vmax series; SensorMedics Corporation, Yorba Linda, California, USA) Miller J 2005 - Spirometry Macintyre N 2005 - DLCO Wanger J 2005 - Lung volumes	Spirometry: GLI z-scores Lung volumes and DLCO: Rosenthal 1993
Molgat-Seon,	n total (n male) Term: 20 (11)	Mean (SD) - weeks Preterm	Mean (SD) Preterm	Not given	Mean (SD) - Years Term:	1979	1997	Not given	Spirometry and whole body	Spirometry: Quanjer 2012

© 2022 Kotecha SJ et al. *JAMA Pediatrics.*

	Preterm without BPD: 19 (12) Preterm with BPD: 25 (13)	without BPD: 28.8 (0.5) Preterm with BPD: 27.2 (0.4)	without BPD: 1240 (100) Preterm with BPD: 990 (50)		24.1 (0.5) Preterm without BPD: 21.2 (0.6) Preterm with BPD: 21.8 (0.7)				plethysmograp hy were performed using a commercially available system (Control: VmaxEncore 229, V62J Autobox; CareFusion, Yorba Linda,CA; Preterm: Elite Series Plethysmograp h; MedGraphics, St.Paul, MN) according to standard recommendatio ns.	
Morata-Alba,	Not given	Preterm: 32+1 to 35+0 (Nil further details)	Grams: Mean (SD) Preterm: 1941.65 (383.83) Term: 3377.38 (405.29)	Duration not reported In preterm group 23 (19.8%) received invasive mechanical ventilation and 27 (23.3%) received non-invasive mechanical ventilation	Preterm infants 6/7/8 years: N = 16 (13.8%); N = 19 (16.4%); N = 81 (69.8%) Full term infants 6/7/8 years: N = 4 (3.4%); N = 0; N = 112 (96.6%) p < 0.001	2006	2008	Not given	"At the last visit at 7-8 years of age, all the children underwent forced spirometry with a BDT (Master V5.1 Spirometer, Viasys Healthcare, Würzburg, Germany)"	Reference equations not referenced
Moreno-Galdo	For the 670 who completed 3 years of follow up 386/670 male (57.6%)	32 - 35 weeks gestation for the 670 who completed 3 years of follow up mean 33.7 SD 0.9	For the 670 who completed 3 years of follow up mean 1962.6 SD 393.2 g	For the 670 who completed 3 years of follow up 54/670 (8.1%) ventilated	approx 3 years	2006	2008	Not given	Pulmonary function testing was also performed in the centers with available spirometry using a Jaeger MasterScope	Z-scores were calculated based on Stanojevic's Equations,

© 2022 Kotecha SJ et al. *JAMA Pediatrics.*

									spirometer (CareFusion) or a Datospir-600 spirometer (Sibel Group), according to ERS/ATS guidelines. The following spirometry values were collected: forced vital capacity (FVC), forced expiratory volume at 0.5 seconds (FEV0.5), and forced expiratory flow, mid-expiratory phase (FEF25-75).	
Morris	Not given	<29 weeks	Not given	Not given	16–18-year-olds	Not given	Not given	more than 90% were exposed to both antenatal steroids and postnatal surfactant	Not given	Results were converted to z-scores, corrected for height, sex and ethnicity using GLI reference ranges
Morsing,	PT-IUGR 16/31 girls	PT-IUGR MEDIAN 26.9 range 24-29 PT-AGA matched for GA PT-IUGR MEAN 27.0 SD (1.4) PT-AGA mean 27.0 SD (1.5) T-AGA mean 39.9 SD (0.6)	PT-IUGR MEDIAN 650 range 395-976g PT-AGA median 1010 range 660-1790g T-AGA median 3530 range 3000-4390	days on ventilation did not differ between the groups	Median age 8.4 range 6.5-10.7 PT-IUGR mean 8.4 SD 1.2 PT-AGA mean 8.4 SD 1.3 T-AGA mean 8.4 SD 1.3	1998	2004	Not given	"Pulmonary function was measured using the Vitalograph 2170 spirometer (Spirotrac IV, Vitalograph). The children were tested in a standing position breathing through a	"The measurements were expressed as z-scores and were adjusted for age (corrected for any prematurity), height and gender according to Stanojevic"

© 2022 Kotecha SJ et al. *JAMA Pediatrics.*

									mouthpiece. No nose clips were used. One experienced and trained nurse, who was blinded to the children's background, took the measurements according to ATS⁄ERS standard. The manoeuvres were repeated for at least three but maximal eight times to achieve the best measurements. Respiratory function was assessed from the measurements of forced vital capacity (FVC), forced expiratory volume in 1 sec (FEV1), FEV1 ⁄FVC and forced mid-expiratory flow rate (FEF25-75%)."	
Narayanan,	50.4% were boys	Term means 39.6 SD (1.4) mild preterm 35.2 (1.2) extreme no CLD 29.7 (1.3) EXTREME CLD 28 (2.0)	birthweight centile median (IQR) EXTREME PRETERM WITHOUT CLD 63.4 (34-78) EXTREME PRETERM WITH	MEDIAN (IQR) [RANGE DAYS] EXTREME PRETERM WITHOUT CLD 0 (0-2) [0-8] EXTREME PRETERM WITH	10-14 years mean age 11.85 SD (1.01) Term mean 12.0 SD (1.2) mild preterm 12.2 (0.9) extreme no CLD 11.3	Not given	Not given	EXTREME PRETERM WITHOUT CLD 8/19 (47.1%) EXTREME PRETERM WITH CLD 14/18 (82.4%)	" We performed anthropometric measurements, spirometry, and plethysmograp hy according to	" We calculated within-sample z scores for lung function measurements that were adjusted for

© 2022 Kotecha SJ et al. *JAMA Pediatrics.*

			CLD 36 (20-56) No birthweight for mild preterm or term controls	CLD 7 (5-19) [0-44]	(0.6) EXTREME CLD 11.6 (0.6)				standardized guidelines."	age, height, sex, and ethnicity"
Nasanen-Gilmore,	# (# m) <34 weeks: 139 (66) 34-37 weeks: 239 (118) ≥37 weeks: 341 (164)	mean (sd) <34 weeks: 1777 (482) 34-37 weeks: 2670 (521) ≥37 weeks: 3583 (485)	days on ventilator <34 weeks: <7 days: 51 7 to ≤ 14: 9 >14: 7 34-37 weeks: <7: 26 7 to <14: 3 ≥14: 0 ≥37 weeks: <7: 2 7 to <14: 0 ≥ 14: 0	mean (sd) <34 weeks: 23.1 (1.4) 34-37 weeks: 23.2 (1.2) ≥37 weeks: 23.5 (1.1)	1985	1986	None (pre-surfactant era cohort)	Spirometry (n= 724) was performed (Medikro Windows, Spiro 20001.3) sitting upright following the standard spirometry technique described by the European Respiratory Society Guidelines	GLI - Quanjer 2012	
Nixon,	84 males unexposed to ANCS 39 (42%) exposed to ANCS 45 (48%)	unexposed to ANCS median (5th, 95th percentile) 27(23,33) exposed to ANCS 28 (24,32)	unexposed to ANCS median (5th,95th percentile) 1043 (595,1461) exposed to ANCS 1003 (618,1466)	unexposed to ANCS median (5th,95th percentile) 4 (0, 60.3) days exposed to ANCS 5 (0,55.3)	14 years	1992	1996	unexposed to ANCS 59 (63%) exposed to ANCS 58 (62%)	"Pulmonary function testing was conducted in accordance with standard guidelines using a Viasys Vmax Encore Metabolic Cart equipped with a heated-wire pneumotachometer and spirometry software"	Values were expressed as a % of predicted based on reference data specific for gender, race, age, and height and values below the 5th percentile were considered below normal."
Nixon,	males 52% of dex group and 49% placebo group	dex median (5th, 95th percentile) 25 (23, 28) placebo (26 (23, 30)	dex median (5th, 95th percentile) 732g (527, 1182) placebo (789 (553, 1298)	Not given	8-11 years dex median (5th, 95th percentile) 9.3 (8.0, 10.9) placebo 9.3 (8.2, 10.9)	1992	1995	Not given	not stated in this paper but referenced	not stated in this paper but reference
Nordlund,	BPD 20 males Asthma 19 males	BPD 26.6 weeks, mild mean 27.1 SD	BPD mild mean 1128 SD 293,	assisted ventilation median IQR	BPD mean 10.4 years SD 1.0	1998	1999	82% mild BPD group 63%	"Pre-bronchodilator lung function	"Pre-bronchodilator lung function

© 2022 Kotecha SJ et al. *JAMA Pediatrics.*

		1.6, moderate/severe 26.1 (2.6) asthma group born at term,	moderate/severe 924 SD 343	days mild 5 (8), moderate/severe 14 (14)	asthma mean 10.7 SD 0.7			moderate/severe BPD group	was assessed by dynamic spirometry (Vitalograph® 2120 Ennis of Ireland) and included measurements of forced vital capacity (FVC), FEV1%, EV1/FVC%, forced expiratory flow of 50% (FEF50%), utilizing European Respiratory Society and Polgar reference values. Plethysmography (CareFusion, Hoechberg, Germany) was used to measure total lung volumes including vital capacity (VC), total lung capacity (TLC), functional residual capacity (FRC), and residual volumes (RV). Diffusion capacity for carbon monoxide (DLCO) was measured using the single-	was assessed by dynamic spirometry (Vitalograph® 2120 Ennis of Ireland) and included measurements of forced vital capacity (FVC), FEV1%, EV1/FVC%, forced expiratory flow of 50% (FEF50%), utilizing European Respiratory Society and Polgar reference values. Plethysmography (CareFusion, Hoechberg, Germany) was used to measure total lung volumes including vital capacity (VC), total lung capacity (TLC), functional residual capacity (FRC), and residual volumes (RV). Diffusion capacity for carbon monoxide (DLCO) was measured using the single-

© 2022 Kotecha SJ et al. *JAMA Pediatrics.*

									breath technique, according to international guidelines. The reference values of Hedenström Solymar were applied to static lung volumes and DLCO."	breath technique, according to international guidelines. The reference values of Hedenström Solymar were applied to static lung volumes and DLCO."
Panagiotounakou,	Male (%) Term: 39 (62.9) Preterm: 63 (58.3) Preterm with BPD: 29 (59.2) Preterm without BPD: 34 (57.6)	Mean (SD) [range] Term: Not given Preterm: Not given Preterm with BPD: 27.6 (1.7) [23+5 to 32] Preterm without BPD: 30.9 (1.2) [29 to 32]	Mean (SD) Term: Not given Preterm: Not given Preterm with BPD: 1003.8 (186.2) Preterm without BPD: 1346.5 (284.2)	Mean (SD) Preterm with BPD: 9.5 (13.9) Preterm without BPD: 0.91 (1.46)	8-10 years Preterm mean 8.4 years (SD 0.8) Term mean 8.3 years (SD 1.2) (BPD n=42, non-BPD n=43, Term n=62) **NOT full cohort tested**	2007	2009	Not given	The Spirolab spirometer of Mir technology was used for the spirometry according to ATS and ERS guidelines.	Respiratory function expressed as the percentage of predicted values for height, weight, and sex
Prais,	n total (n male) Palivizumab: 30 (17) No palivizumab: 33 (16)	mean (SD) Palivizumab: 26.5 (1.2) No palivizumab: 26.7 (1.2)	mean (SD) - grams Palivizumab: 889 (167) No palivizumab: 944 (205)	median [range] - days Palivizumab: 16 [0 - 58] No palivizumab: 8 [0 - 81]	mean (SD) - years Palivizumab: 8.87 (0.6) No palivizumab: 8.84 (0.8)	2000	2003	n (%) Palivizumab: 23 (79%) No palivizumab: 28 (87%)	Pulmonary function was measured in the lung function laboratory of the hospital's Pulmonary Institute according to the American Thoracic Society guidelines. 17,18 Spirometry was performed with a ZAN100 flow sensor (ZAN Messgeräte GmbH), and plethysmography and	Spirometry: % predicted - Varuni 1971 Plethysmography: % predicted - Varuni 1971 DLCO: % predicted - Levinson 1969

© 2022 Kotecha SJ et al. *JAMA Pediatrics.*

									measurement of the diffusing capacity of the lung for carbon monoxide (Dlco) were performed with a constant-volume body plethysmograph and a Dlco unit (ZAN500; ZAN Messgeräte GmbH).	
Praprotnik,	Preterm group 50% male BPD 15m 8f preterms no BPD 13m 20f All preterms 28m 28f controls 13m 20f	Preterm born at 24-30 weeks gestation preterm group mean 26.7 SD 1.7 BPD 26.2 SD 2.4 Preterm no BPD 27.2 SD 1.7 Terms >37 weeks gestation	Preterm group mean 996g SD 246 BPD 867 SD 276 Preterm no BPD 1056 SD 252	"The length of ventilatory support (32 [95% confidence interval 18.0 to 49.0] days vs 5.5 [3.5 to 21.5] was significantly longer in the BPD group	Preterms 7.6 years 0.9 controls 8.0 (1.0) BPD 7.5 (1.0) preterms no BPD 7.7 (0.8)	2000	2002	86% bpd 9% non-BPD group	"Blinded to the clinical details of the participant. Flow-volume spirometry (Vitalograph, Birmingham, UK) was used, which was performed in accordance with the American Thoracic Society guidelines. The best of three manoeuvres was recorded. The following variables reflecting airflow were measured: the forced expiratory volume in one second (FEV1) and the forced	"Blinded to the clinical details of the participant. Flow-volume spirometry (Vitalograph, Birmingham, UK) was used, which was performed in accordance with the American Thoracic Society guidelines. The best of three manoeuvres was recorded. The following variables reflecting airflow were measured: the forced expiratory volume in one second (FEV1) and the forced

© 2022 Kotecha SJ et al. *JAMA Pediatrics.*

									expiratory flow between 25 and 75% of the forced vital capacity (FEF25-75). The measured lung volumes included the forced vital capacity (FVC) and the vital capacity (VC). The results were expressed as percentages of the standardized values predicted for normal children according to sex and height. The prediction equations of Polgar and Promadhat were used for the lung-function measurements. "	expiratory flow between 25 and 75% of the forced vital capacity (FEF25-75). The measured lung volumes included the forced vital capacity (FVC) and the vital capacity (VC). The results were expressed as percentages of the standardized values predicted for normal children according to sex and height. The prediction equations of Polgar and Promadhat were used for the lung-function measurements. "
Prenzel,	n total (n male) BPD: 42 (19) Term: 42 (17)	mean (range) - weeks BPD: 26.6 (24 - 31) Term: 39.8 (37 - 42)	mean (range) - grams BPD: 862.8 (487 - 1375) Term: 3505.4 (2705 - 4630)	mean (range) - days BPD: 8.8 (0-33) Term: 0	mean - years BPD: 11.5 Term: 11.8	1994	2002	n (%) BPD: 28 (67%)	All study participants performed flow volume spirometry using a spirometer (Jaeger, MasterScope, Wuerzburg, Germany). Short-acting β-agonists were withheld one	Z-scores: Quanjer 2012

© 2022 Kotecha SJ et al. *JAMA Pediatrics.*

									day before testing. Children older than 9 years had to fulfil general ERS/ ATS quality criteria (Miller 2005), and children younger than 9 years had to meet ERS/ATS criteria for children according to Beydon and colleagues (Beydon 2007).	
Ronkainen,	n total (n male) Preterm: 88 (47) Term: 88 (47)	mean (SD) - weeks Preterm: 28.8 (2.1) Term: 39.9 (1.2)	mean (SD) - grams Preterm: 1133 (409) Term: 3574 (514)	mean (SD) Preterm: 9.0 (13.2)	mean (SD) - years Preterm: 10.9 (1.4) Term: 11.6 (1.7)	1997	2003	n (%) Preterm: 56 (64%)	Spirometry was recorded using USB spitometry (SpiroStar USB, Medikro Oy, Kuopio, Finland) DLCO was measured using single breath technique using Jaeger Master-Screen PFT device (Viasys Healthcare, GmbH, Hoechberg, Germany) and corrected for hemoglobin if there was a history of anaemia. Measurements were performed according to	Spirometry: % predicted (Koillinen 1998 "Terveiden suomalaislasten spirometrian ja uloshengitykse n huippuvirtaukse n viitearvot.") DLCO: % predicted (Polger 1971)

© 2022 Kotecha SJ et al. *JAMA Pediatrics.*

									ATS and ERS guidelines	
Ruf,	n (% total) BPD (n=9): 4 (40%) No-BPD (n=13): 7 (47%) Control (n=15): 8 (53%)	Mean (SD) [range] BPD: 26.6 (1.6) [24.6 - 29.7] No-BPD:29.1 (1.8) [25.0 - 31.0] Control: 39.3 (0.9) [38.0 - 41.0]	Mean (SD) [range] BPD: 766.7 ± 212.4 (450-1080) No-BPD: 117.3 ± 242.6 (780-1440) Control: 3382.7 ± 327.5 (2800-3950)	SIMV BPD (n=9/9): 24.9 (14.4) [2-47] No-BPD (n=8/13): 3.7 (5.3) [0-19] Control (n=0/15) HFOV BPD (n=4/9): 3.4 (5.0) [0-12] No-BPD (n=0/13) Control (n=0/15):	BPD: 10.9 (1.7) No-BPD: 10.4 (1.5) Control: 9.9 (1.3)	1997	2001	BPD: 8/9 No-BPD: 4/13 Control: 0/15	The Jaeger MasterScreen System (CareFusion, Hoechberg, Germany) was used to measure spirometric data, static lung volumes, airway resistance and diffusing capacity for carbon monoxide (TLCO). Spirometry has been applied to assess forced expiratory volume in 1 s (FEV1) and forced vital capacity (FVC), which reflect a possible obstructive or restrictive airway disease. Likewise, static lung volumes were assessed using bodyplethysmography. Residual volume (RV) and total lung capacity (TLC) were analysed to gain information	All lung function testing was performed according to current standards, values are expressed as percent of predicted values, limits of normal ranges were determined according to Pellegrino et al.

© 2022 Kotecha SJ et al. *JAMA Pediatrics.*

									about restrictive airway disease or a possible hyperinflation. Especially, RV to TLC ratio (RV%TLC) indicates a pulmonary hyperinflation.	
Simpson,	Total # of participants (# male) Term: 58 (26) Preterm (all): 163 (100) Preterm no BPD: 64 (42) Preterm with BPD: 99 (58)	Median (IQR) - weeks Preterm (all): 28.0 (25.0, 29.6) Preterm no BPD: 29.8 (28.6, 31.0) Preterm with BPD: 26.0 (24.4, 27.6)	Median (IQR) - grams Preterm (all): 955 (755, 1335) Preterm no BPD: 1388 (1186, 1645) Preterm with BPD: 825 (700, 955)	Median (IQR) - days Preterm (all): 4 (0, 28) Preterm no BPD: 0 (0, 1) Preterm with BPD: 20 (5, 39)	Mean (SD) - years Term: 10.7 (0.9) Preterm (all): 10.9 (0.6) Preterm no BPD: 10.9 (0.6) Preterm with BPD: 10.8 (0.6)	1997	2003	n (%) Preterm (all): 115 (70.6) Preterm no BPD: 27 (42.7) Preterm with BPD: 88 (89.8)	Lung function tests were performed according to American Thoracic Society/European Respiratory Society standards with spirometry, multiple breath washout, and DLCO using Sensormedics Vmax, Yorba Linda, California, USA) and forced oscillation technique using I2M, Chess Medical, Ghent, Belgium	Lung function outcomes were expressed as z-scores to adjust for the relevant anthropometric factors, with the exception of Lung Clearance Index (LCI), residual volume (RV)/total lung capacity (TLC) and functional residual capacity (FRC)/TLC that are independent of anthropometrics. Spirometry - Quanjer 2012 (GLI) DLCO - Kim 2012 FOT - Calogero 2013
Sorensen,	n total (n male) BPD: 42 (23) non-BPD: 28 (20) Control: 38 (14)	mean (SD) - weeks BPD: 26.8 (1.6) non-BPD: 26.9 (1.9) Control: 39.9 (1.2)	mean (SD) - grams BPD: 863 (219) non-BPD: 987 (224) Control: 3519 (382)	mean (SD) - Intubation days BPD: 11.9 (22.0) non-BPD: 4.7 (12.4) Control: 0	mean (SD) - years BPD: 11 (1.5) non-BPD: 11.1 (1.4) Control: 10.9 (1.1)	2002	2006	Not given	Spirometry, whole-body plethysmography, and DLCO and DLNO measurements were performed using Jaeger	Spirometry: Stanojevic 2008 Plethysmography: Koopman (7) DLCO/DLNO: Thomas (4)

© 2022 Kotecha SJ et al. *JAMA Pediatrics.*

									Master Screen Pro (CareFusion, Hochberg, Germany) following ATS and ERS recommendations, with the exception that DLNO was measured as described by Thomas et al. EP participants were asked to perform a test for bronchodilator responsiveness involving inhalation of Salbutamol (200 µg). A positive bronchodilator responsiveness test was defined as an increase of >12% in FEV1.	
Teig	Preterm group 8/16 male term group 6/11 male	Preterm group mean 29.4 SD 1.5 Term group term	Terms had a birthweight >3000g preterm group mean 1342g SD 228	Preterm group median 10 days IQR 4.3-13.5	preterm median age of 11 years and terms were age matched Preterm mean 11 SD 1.63 years Terms 12.3 SD 1.4 years	1988	1991	Preterm 8/16 given surfactant	"Participants performed spirometry (jaeger Bodyscreen, Viasys Healthcare, Wurzburg, Germany) prior to the inhalation procedure. Three acceptable forced	"Values were expressed as percent predicted and as z-scores using the software of the UK Asthma Inititiative (growth charts for lung function v 2.01.97) which is based on reference data

© 2022 Kotecha SJ et al. *JAMA Pediatrics.*

									expiratory flow (FEF) curves were recorded according to current recommendations. The measurement with the highest sum of FEV1 and FVC was accepted fur further analysis. FEV1 and FEF25-75 were recorded from this best curve."	published by stanojevic et al"
Thunqvist	Preterm 82/153 m term 90/157 male	Preterm mean 25 range 22-26 term mean 39.8 range 37-41	Preterm mean 782 SD 171 term 3583 SD 467	Preterm mean 14.2 days SD 15.9 term 0	6 1/2 years +/- 3 months mean (SD) preterm mean 6.6 (0.2) term 6.6 (0.2)	2004	2007	Preterm 151/153 term 0	"Lung function tests consisted of dynamic spirometry and impulse oscillometry (IOS). The tests were performed using the Jaeger MasterScreen-IOS system (Carefusion Technologies, San Diego, CA, USA). Spirometry was performed according to ATS/ERS criteria. The test subject performed at least three maximum expiratory flow volume recordings in	"In addition to quantitative data, FVC, FEV1, and FEV1/FVC were converted to z-scores using the Global Lung Initiative reference values (GLI)."

© 2022 Kotecha SJ et al. *JAMA Pediatrics.*

									sitting position, wearing a nose clip. The highest values of forced vital capacity (FVC) and forced expiratory volume in 1 s (FEV1) were extracted and used for analysis, provided that the subject's effort was coded as being maximal by the test leader, the curve passed visual quality inspection, and that the two highest FVC and FEV1 readings were reproducible."	
Thunqvist,	Preterm females 73 males 76 Terms females 1287 males 1185	Preterm females mean (range) 34.9 32-36 Terms females 39.6 37-41 preterm males 35.1 32-36 term males 39.6 37-41	Preterm females mean (SD) 2582 (476) Terms females 3512 (477) preterm males 2624 (515) term males 3613 (476)	Not given	Preterm females mean (SD) 8.4 (0.5) and 16.7 (0.4) Terms females 8.3 (0.5) and 16.7 (0.4) preterm males 8.4 (0.4) and 16.7 (0.4) term males 8.4 (0.5) and 16.7 (0.4)	1994	1996	Not given	"At 8 years of age, patients underwent lung function testing, performed by using the 2200 Pulmonary Function Laboratory (SensorMedics, Anaheim, CA), and at 16 years of age, using the Jaeger MasterScreen-IOS system (Carefusion Technologies,	"z-scores according to the Global Lung Initiative reference values were also evaluated"

© 2022 Kotecha SJ et al. *JAMA Pediatrics.*

									San Diego, CA).22 At both occasions, spirometry was performed according to American Thoracic Society/European Respiratory Society criteria. The highest values of forced vital capacity (FVC) and forced expiratory volume in 1 second (FEV1) were used for analysis. FEV1/FVC ratios were expressed as percentages. Midexpiratory flow rate was extracted from the curve with the highest sum of FEV1 and FVC."	
Tukova,	Budesonide group 15/26 female Placebo 8/22 female	Budesonide group median 26 range 24-27 Placebo median 25 range 23-27	Budesonide group median 779 range 570-1270 Placebo median 790 range 530-1050	Ventilatory support (days) Budenoside median 47 range (6-70) Placebo 50.5 (3-165)	mean 5.9 range 5-7 years	2010	2013	Not given	Spirometry was performed with the same equipment JAEGER Master-Scope Spirometer according to ATS guidelines for preschoolers8 from April 2018 to April 2019. Flow-volume	The results were presented as Z scores to adjust for body size, age, and sex using an online calculator (provided by the European Respiratory Society, the Global Lung Function

© 2022 Kotecha SJ et al. *JAMA Pediatrics.*

									curves were carried out measuring forced vital capacities and expiratory flows. Forced vital capacity (FVC) was chosen to assess lung volume. Although forced expiratory flow in 1 second (FEV1) and the ratio of forced expiratory flow in 1 second to forced vital capacity (FEV1/FVC) were reported, the required expiratory flow limitation is usually not achieved at FEV1 in young children.9,10 Therefore, forced expiratory flow during mid (25-75%) portion of FVC (FEF25-75) and forced expiratory flow when 75% of FVC has been exhaled (FEF75) were proposed to be more accurate parameters for monitoring of	Initiative, 2012).

© 2022 Kotecha SJ et al. *JAMA Pediatrics.*

									an expiratory flow pattern in young children.	
Turner, Data from author										
Um-Bergstrtom,	BPD 11/26 M preterm 10/23 M asthma 10/23 M Terms 12/24 M	MEDIAN RANGE BPD 26 (24-31) preterm 29.5 (26-32) asthma 40 (38-42) Terms 40 (37-43)	MEDIAN RANGE BPD 960 (583-1510) preterm 1470 (659-2200) asthma 3505 (2660-4840) Terms 3458 (2670-4550)	MEDIAN RANGE BPD 4 (0-38) preterm 0 (0-8)	MEDIAN RANGE BPD 19.6 (18.2-21.2) preterm 19.1 (18.3-22.4) asthma 20.2 (18.6-23.3) Terms 20.5 (18.3-23.8)	1992	1998	MEDIAN RANGE BPD 8/26 preterm 2/23	"Lung function testing was performed according to American Thoracic Society/European Respiratory Society guidelines. Lung function testing included dynamic spirometry (Sensormedics 6200, SensorMedics, Yorba Linda, California, USA) where the highest values of forced expiratory volume in 1 s (FEV1) and forced vital capacity (FVC), were extracted and FEV1/FVC ratio calculated and used for analysis."	"FVC, FEV1 and FEV1/FVC were converted to z-scores using the Global Lung Initiative reference values (GLI)"
Vardar-Yagli,	BPD 10F 8M Controls 12 F 8M	BPD 28.6 +/- 3.4 controls 37.5 +/- 2.3	BPD 1,197.2 +/- 742.5g controls 3,206.2 +/- 348.9g	12/18 BPD were ventilated (66.7%)	6-13 years BPD 8.8 +/- 2.5 years controls 9.0 +/- 1.5 years	Not given	Not given	12/18 BPD (66.7%)	"Pulmonary function testing was performed using a spirometer (SensorMedics, 6200 Body Box, Viasys, USA).	expressed as percentages of the expected values in accordance with a subject's age, height,

© 2022 Kotecha SJ et al. *JAMA Pediatrics.*

									The FVC, FEV1, FEV1/FVC ratio, peak expiratory flow (PEF), and forced expiratoryflow from 25 to 75% (FEF25-75%) were recorded and expressed as percentages of the expected values in accordance with a subject's age, height, body weight and gender"	body weight and gender"
Vollsaeter,	n (n male) Control: 54 (29) All EP: 57 (29) non-BPD: 26 (10) BPD: 31 (19)	mean (SD) All EP: 26.8 (1.6) non-BPD: 27.3 (1.6) BPD: 26.3 (1.4)	mean (SD) All EP; 850 (175) non-BPD 873 (200) BPD: 831 (151) SDS-score All EP: -0.80 (1.3) non-BPD: -0.97 (1.4) BPD: 26.3 -0.66 (1.2)	median (range) - days All EP: 5.0 (0-24) non-BPD: 2.5 (0-21) BPD: 8.0 (0-24)	mean (SD) Control: 11.7 (0.7) All EP: 11.4 (0.6) non-BPD: 11.4 (0.7) BPD: 11.5 (0.6)	1999	2000	n (%) All EP: 49/56 (88) non-BPD: 20/25 (80) BPD: 29 (94)	Spirometry and pulmonary diffusing capacity for carbon monoxide (DLCO) (single breath method) were measured with Vmax 22 equipment (SensorMedics Inc, Anaheim, USA), static lung volumes with V6200 Autobox Body Plethysmograp h (SensorMedics Inc, Anaheim, USA), all in sitting position, wearing a nose clip, applying standard quality criteria [2-6]. The	Spirometry: Quanjer 2012 Plethysmograp hy: Rosenthal 1993 DLCO: Expressed as raw

© 2022 Kotecha SJ et al. *JAMA Pediatrics.*

									spirometry values from the second test day were used, for those subjects (2 EP and 3 term-born) that declined (or were unable to perform) after the first day, values from the first test day were used.	
Vollstaeer,	n total (n female) 10y Preterm: 35 (22) Control: 35 (22) 18y: Preterm: 48 (21) Control: 46 (21)	mean (range) 10y Preterm: 26.9 (23-31) no BPD: 28.3 (26-31) mild BPD: 26.5 (24-28) m/s BPD: 25.8 (23-28) 18y: Preterm: 27.3 (23-32) no BPD: 28.5 (27-32) mild BPD: 27.0 (23-28) m/s BPD: 27.2 (26-30)	mean (range) 10y Preterm: 933 (570 - 1400) no BPD: 1053 (930 - 1400) mild BPD: 927 (620 - 1370) m/s BPD: 851 (570 - 1200) Control: 3564 (3010 - 4000) 18y: Preterm: 1012 (580 - 1480) no BPD: 1151 (960 - 1480) mild BPD: 1013 (580 - 1340) m/s BPD: 892 (670 - 1080) Control: 3441 (3000 - 4000)	days, median (range) 10y Preterm: 4.0 (0-54.5) no BPD: 0.5 (0-1.7) mild BPD: 3.0 (0-40.0) m/s BPD: 12.7 (1.7-54.5) 18y: Preterm: 8.0 (0-54.0) no BPD: 0.3 (0-4.8) mild BPD: 8.6 (0.8-35) m/s BPD: 25.2 (0.7-54.0)	mean (sd) 10y: 10.5 (0.4) 18y: 17.8 (0.4)	1991 1982	1992 1985	n (%) 10y Preterm: 17 (49%) no BPD: 1 (11%) mild BPD: 6 (43%) m/s BPD: 10 (83%) 18y: not given	Spirometry was performed with a Vmax spirometer (SensorMedics, Anaheim, USA) at both visits, according to standard quality criteria. (Quanjer 1993)	Stanojevic 2008
Vrijlandt,	MLP 21M 16F Full term 18M 16F	MLP mean +/- SD 34 +/- 1 WEEK RANGE 32-35 TERM 39 +/- 0.9 RANGE 38-41	MLP mean +/- SD 2442 +/- 539g range 1345-3900 Term 3693 +/- 393g	Not given	13-14 years MLP mean +/- SD 13.6 +/- 0.6 range 12-14 Term 15.5 +/- 0.5 range 12-14	2002	2003	Not given	"All measurements were performed by professional laboratory technicians according to guidelines of the European	"Reference values for lung function were according to Quanjer et al (FVC, FEV1, MEF25) or Zapletal et al (other measures)."

© 2022 Kotecha SJ et al. *JAMA Pediatrics.*

									Respiratory Society. Forced expiratory volume in 1 second (FEV1), maximum expiratory flow (MEF) at 75%, 50%, 25% of Forced Vital Capacity and peak expiratory flow were measured using a pneumotachograph (Jaeger Ltd, Viasys Healthcare GmbH, Hoechberg, Germany). At least 3 similar curves were required before any spirometric test variable was accepted. Reference values for lung function were according to Quanjer et al (FVC, FEV1, MEF25) or Zapletal et al (other measures). Functional residual capacity and ventilation inhomogeneity measurements (LCI 2.5) during normal tidal	

© 2022 Kotecha SJ et al. *JAMA Pediatrics.*

									breathing by multiple-breath nitrogen washout principle using 100% oxygen was performed with the EXHALYZER D device (Eco Medics AG, Dürnten, Switzerland) and its associated software (Spiroware 3.2; Eco Medics AG). LCI 2.5 was measured according to the European Respiratory Society/American Thoracic Society consensus statement.	
Winck,	Not given	VLBW mean 30.4 ± SD 4.5	VLBW mean 1.210.42 ± SD 168.72	VLBW 31/48 (64.6%)	8-11 YEARS OF AGE Controls mean 10.23 SD 1.27 VLBW mean 10.18 SD 1.39	2001	2005	VLBW 31/48 (64.6%)	" Spirometry was performed with a portable spirometer (Koko; Ferraris Respiratory, Louisville, CO, USA). All tests were performed in accordance with the American Thoracic Society standards and acceptability and	The results were expressed as absolute values and normalized to Z scores.

© 2022 Kotecha SJ et al. *JAMA Pediatrics.*

									reproducibility criteria. The children were verbally encouraged to perform a maximal expiratory maneuver at maximal effort following a maximal inspiratory maneuver. The following spirometric parameters were assessed: FVC; FEV1; and FEF25-75%.	
Yaacoby-Bianu,	Late preterms 17/29 58% male terms 15/30 50% males	Late preterms mean 35.5 (0.9) weeks Terms 39.6 (1.1)	Late preterms mean 2336g (400) Terms 3386g (485)	Not given	6 to 12 years Late preterm mean 8.2 (1.7) years terms 8.8 (1.8) years	2005	2010	Not given	"Spirometry was performed in accordance with the American Thoracic Society/European Respiratory Society (ATS/ERS) Task Force, using a KoKo spirometer (n-Spire Healthcare, Inc, Longmont, CO). "	"Results were expressed as absolute values and z-scores derived from Polgar and Quanjer"
Yang	VLBW 99/226 male controls 37/100	VLBW mean 29.2 weeks SD 2.5 terms term	VLBW means 1132g (238) Term 3372 (565)	147 were ventilated mean 16.6 days (16.4)	26 to 30 years VLBW mean 28.5 SD 1.1 years controls mean 28.3 SD 0.9 years	1986	1986	Not available when participants were born	" Lung function tests were undertaken at the Respiratory Physiology Laboratory, Christchurch Hospital which is accredited by	"Values for spirometry, lung volumes and DLCO were presented as z scores and percentages of the predicted on the basis of

© 2022 Kotecha SJ et al. *JAMA Pediatrics.*

									the Thoracic Society of Australia and New Zealand. All tests were performed on 1 days in the following order: spirometry, plethysmographic lung volumes, single-breath diffusing capacity of the lung for carbon monoxide (DLCO) (all with Autobox V62); CareFusion, Yorba Linda, CA), and single-breath nitrogen washout (SBN2) (SensorMedics Vmax Spectra exercise test station; CareFusion). The tests were performed with the subject sitting and a nose clip in place; respiratory physiologists were blinded as to the participants group. Testing protocols adhered to the American Thoracic Society and European	references values from the GLI 2012, Quanjer et al (193), and GLI (2017) respectively"

© 2022 Kotecha SJ et al. *JAMA Pediatrics.*

									Respiratory Society pulmonary function standards and interpretation"	
Hacking	Preterm 50.7% female<br>Controls 47.7% female	Preterm mean 26.5 weeks SD 1.9<br>Controls 39.3 (1.1)	Preterm mean 835 (165)<br>Controls 3,508 (473)	Not given	Preterm 8.4 (0.4)<br>Controls 8.5 (0.4)	1997	1997	Preterm 84.%<br>Controls 0.7%	"Respiratory function was measured accord-<br>ing to relevant guidelines by specialist respiratory scientists who were blind to clinical details of the sub-jects; only acceptable measures have been reported. Spirometry and lung volumes were measured using a Jaeger Body-screen II Bodybox (Jaeger, Germany) with JLAB version 4.54 software. Maximum expiratory flow–volume curves were recorded while the subject sat in the body plethysmograp h with the door open. Flow was measured with a pneumotachogr	"Results at body temperature and pressure saturated with water vapor were expressed as SD scores to adjust for height, sex, and age."

© 2022 Kotecha SJ et al. *JAMA Pediatrics.*

									aph, and volume was obtained by integration of flow.”	
Bui	Data from authors	Pretem mean 35.2 SD 1.5 range 28-36 Term mean 39.9 SD 1.1	Data from authors	Data from authors	Preterm mean 52.9 SD 0.9 Term mean 52.9 SD 0.9	1961	1961	Data from authors	Data from authors	Data from authors

© 2022 Kotecha SJ et al. *JAMA Pediatrics.*

eTable 6. Lung function outcomes of the included articles

**Author, Year Published, Country**	FEV_1 predicted	FVC	FEF 25-75	FEV1/FVC	RV	DLCO
Aoyama	Z score mean -1.31 SD 1.39 (range -5.05 – 2.67)	Z score mean -0.72 SD 1.59 (range -4.48 – 5.46)	Z score mean -1.40 SD 1.27 (range -4.15 – 1.65)	Z score mean -0.89 SD 1.61 (range -3.72 – 2.06)	X	X
Arroyas	<32 weeks group %pred mean 94.8 (SD 13.3) 32-36 weeks group 95.1 (13.9)	<32 weeks group %pred mean 94.3 (SD 13.6). 32-36 weeks group 94.7 (13.6)	<32 weeks group %pred mean 87.2 (SD 21.5) 32-36 weeks group 94.1 (24.7)	<32 weeks group %pred mean 99.9 (SD 9.1) 32-36 weeks group 100.1 (9.4)	X	X
Durlak	z-score BPD group n=6 mean -1.15 (SD 1.12), No BPD group n = 22 -0.39 (1.28), Controls n = 30 -0.06 (0.85)	z-score BPD group n=4 mean -0.63 (SD 0.95), No BPD group n = 15 0.08 (1.47), Controls n = 30 -0.11 (0.87)	X	X	X	X
Filippo	All preterms z-score mean 0.5 (SD 1.3). Preterms with BPD 0.1 (1.1) Preterms No BPD 0.6 (1.3). Terms 0.7 (0.9)	All preterms z-score mean 0.2 (SD 1.2). Preterms with BPD -0.1 (1.1) Preterms No BPD 0.2 (1.2). Terms 0.2 (0.6)	All preterms z-score mean 0.3 (SD 0.9). Preterms with BPD -0.1 (0.5) Preterms No BPD 0.3 (0.9). Terms 0.5 (0.7)	All preterms z-score mean 0.6 (SD 1.0). Preterms with BPD 0.4 (0.6) Preterms No BPD 0.6 (1.1). Terms 0.7 (0.9)	All preterms % mean 105.9 (SD 54.4). Preterms with BPD 103.2 (23.3) Preterms No BPD 106.2 (56.9). Terms 106.9 (32.6)	X
Hart,	Preterm-born low lung function mean 75.6 (74 – 77.1), preterm-born controls 96.6 (95.8 – 97.4), term-born 95.7 (94.2 - 97)	Preterm-born low lung function mean 83.2 (81.8 – 84.6), preterm-born controls 98.2 (97.2 – 99.1), term-born 96.2 (94.8 – 97.7)	Preterm-born low lung function mean 57.0 (54.2 – 59.7), preterm-born controls 84.0 (82.3 – 85.6), term-born 86.4 (83.6 – 89.1)	Preterm-born low lung function mean 0.80 (0.78 - 0.81), preterm-born controls 0.86 (0.86 – 0.87), term-born 0.87 (0.86 – 0.88)	X	X
Joshi	CLD group mean 81.9 (95% CI 77.1 – 86.7), Preterm group 92.0 (87.0 – 97.0), Term group 97.5 (93.2 – 101.9)	CLD group mean 98.9 (95%CI 94.7 – 103.1), Preterm group 100.8 (96.8 – 104.9), Term group 102.0 (97.2 – 106.8)	CLD group mean 49.2 (95%CI 43.1 – 55.4), Preterm group 69.2 (60.5 – 78.0), Term group 80.0 (73.6 – 86.4)	X	CLD group mean 131.6 (95%CI 110.0 – 153.1), Preterm group 103.2 (91.8 – 114.7), Term group 96.9 (85.6 – 108.1)	CLD group mean 88.0 (95%CI 83.7 – 92.3), Preterm group 95.9 (91.9 – 100), Term group 93.5 (88.6 – 98.4)
Naples	Median z score -1.5 (IGR -2.3, -0.6)	X	MEF25-75 Median z score -1.8 (IQR -2.1, -1.3)	X	X	KCO z score median -1.8 (IQR -2.3, -1.5)
Perez-Tarazona	% pred mean(SD) EP-BPD 85.4 (15.2), EP-NoBPD 94.7 (12.5), MLP 97 (14.2)	% pred mean(SD) EP-BPD 91.7 (14.9). EP-NoBPD 97.4 (11.9), MLP 97.7 (14.1)	% pred mean(SD) EP-BPD 69.6 (23.4), EP-NoBPD 82.7 (22.6), MLP 89.5 (24.5)	% mean(SD) EP-BPD 81.8 (9.48), EP-NoBPD 85.3 (7.95), MLP 86.7 (7.33)	% pred mean (SD) EP-BPD n=44 158.2 (49), EP-NoBPD n=41 142.7 (43.7), MLP n=36 142.4 (47.8)	% pred mean (SD) EP-BPD n=34 91.6 (13.3), EP-NoBPD n=38 93.6 (17.8), MLP n=29 110.3 (16.1)
Santema	Non-BPD group z score mean -0.27 (SD 1.24) BPD group -2.00 (1.61)	Non-BPD group z score mean -0.28 (SD 1.18) BPD group -1.18 (1.29)	X	Non-BPD group z score mean 0.00 (SD 1.14) BPD group -1.28 (1.60)	X	Non-BPD group z score mean -0.16 (SD 0.78) BPD group -0.80 (0.83)
Vanhaverbeke	No BPD group median 104 (range 69 – 121), BPD group 98 (80 – 125)	No BPD group median 111 (range 67 – 122), BPD group 108.5 (77 – 138)	X	No BPD group median 98 (range 88 – 110), BPD group 87 (68 – 111)	No BPD group median 107 (range 56 – 179), BPD group 135.5 (80 – 207)	No BPD group median 87% (range 64 – 127), BPD group 78 (58 – 102) KCO

© 2022 Kotecha SJ et al. *JAMA Pediatrics.*

Hagman,	52 children no BPD 29 BPD 23 median (range) all 92 (70-120) No BPD 92 (70-119) BPD 91 (72-120)	52 children no BPD 29 BPD 23 median (range) all 98 (73-124) No BPD 97 (73-113) BPD 99 (81-124)	52 children no BPD 29 BPD 23 median (range) all 70 (34-130) No BPD 76 (36-130) BPD 65 (34-117)	52 children no BPD 29 BPD 23 median (range) all 0.80 (0.60-0.99) No BPD 0.82 (0.66-0.99) BPD 0.79 (0.60-0.93)	43 children no BPD 22 BPD 21 median (range) all 105 (62-175) No BPD 108 (72-175) BPD 104 (62-155)	47 children no BPD 27 BPD 20 median (range) all 98 (71-121) No BPD 96 (76-121) BPD 95 (71-109)
Hayden,	Mean pre-bronchodilator 81% (SD 18) 113 tests improved postbronchodilator mean change was 15% (12)	88% (SD 18) prebronchodilator	X	83 (SD 11) prebronchodilator	X	X
Vrijlandt,	Preterm 92 (12) Term 97 (10)	Preterm 91 (12) Term 94 (10)	X	X	X	X
Arigliani	The BPD group had lower mean FEV1 (by − 1.20 z-scores, 95% CI-: − 1.98; − 0.82), than the group without BPD, whereas zFEV1 mean (SD) Preterms−0.41 ± 1.13 controls 0.26 ± 0.83 BPD −1.18 ± 0.85 No BPD 0.02 ± 1.05	The BPD group had lower mean FVC (by − 1.05 z-scores,95% CI − 1.58; − 0.51), than the group without BPD, whereas Preterms −0.31 ± 1.00 Controls 0.16 ± 0.76 BPD −0.98 ± 0.71 No BPD 0.07 ± 0.95	X	Preterms z-score −0.18 ± 1.05 Controls 0.09 ± 0.64 BPD −0.41 ± 0.92 no BPD −0.04 ± 1.11	X	X
Arroyas Sanchez,	FEV1 89,8 (68,3-104,2),	FVC 87,3 (62,9-100,5),	X	X	X	X
Bar-Yoseph,	Mean (SD) p- 84.2 ± 18.3 p+ 86.7 ± 10.7	X	Mean (SD) p- 90.0 ± 22.8 p+ 90.2 ± 22.6	Mean (SD) p- 0.96 (0.90-0.99) 110.3 (5.8) p+ 0.94 (0.90-0.99) 107.9 (7.8)	X	X
Bozzetto,	mean and SD z-scores BPD -2 (1.4) Asthmatic -0.54 (1.23) controls 0.69 (0.9)	mean and SD z-scores BPD -1.29 (1.23) Asthmatic 0.02 (1.12) controls 0.33 (1.07)	mean and SD z-scores BPD -2.12 (1.45) Asthmatic -1 (1.12) controls 0.66 (0.96)	mean and SD z-scores BPD -1.35 (1.4) Asthmatic -0.84 (1.19) controls 0.6 (1)	X	X
Cardoen,	there was significant worsening in FEV1 from a mean of 71.34% pred (SD 18.27) to 66.66% pred (SD 21.71) (p<0.05) further information available on LF for early and late cohort	FIRST MEASUREMENT 83.26 (17.29) % second measurement 81.67 (16.34) further information available on LF for early and late cohort	X	there was significant worsening in FEV1/FVC from 85.38% pred (SD 15.15) to 79.8% pred (SD 17.32) (p = 0.01). further information available on LF for early and late cohort	X	X
Cazzato,	Mean (SD) No BPD: -0.93 (1.33) *** BPD: -1.37 (0.77) *** All VLBW: -1.13 (1.13) *** Control: 0.18 (0.89) *P<0.05 vs controls **P<0.01 vs controls ***P<0.001 vs controls	Mean (SD) No BPD: -1.06 (1.26) ** BPD: -1.46 (0.93) *** All VLBW: -1.24 (1.14) *** Control: 0.11 (1.08) *P<0.05 vs controls **P<0.01 vs controls ***P<0.001 vs controls	Mean (SD) No BPD: -0.53 (1.29) * BPD: -0.97 (1.05) ** All VLBW: -0.72 (1.20) *** Control: 0.15 (0.80) *P<0.05 vs controls **P<0.01 vs controls ***P<0.001 vs controls	Mean (SD) No BPD: 0.16 (1.07) BPD: 0.12 (1.23) All VLBW: 0.15 (1.14) Control: 0.03 (0.78) *P<0.05 vs controls **P<0.01 vs controls ***P<0.001 vs controls	Mean (SD) No BPD: 0.40 (1.23) *** BPD: 1.10 (1.81) *** All VLBW: 0.71 (1.53) *** Control: -0.35 (1.39) *P<0.05 vs controls **P<0.01 vs controls ***P<0.001 vs controls	Mean (SD) No BPD: -2.03 (1.13) BPD: -2.33 (1.60) All VLBW: -2.14 (1.29) ** Control: -1.16 (1.34) *P<0.05 vs controls **P<0.01 vs controls ***P<0.001 vs controls

© 2022 Kotecha SJ et al. *JAMA Pediatrics.*

Chang	z scores term 0.04 ± 1.18 preterm −0.73 ± 1.12 preterm subgroup </= 28 weeks -0.89 ± 1.08 29-36 weeks -0.55 ± 1.16 preterm </= 1kg -1.17 ± 0.98 preterm 1,001-1,500g -0.38 ± 1.12 preterm BPD subgroup BPD -0.96 ± 1.08 NO BPD -0.31 ± 1.10	z scores term -0.12 ± 1.14 preterm −0.60 ± 1.20 preterm subgroup </= 28 weeks -0.64 ± 1.20 29-36 weeks -0.56 ± 1.22 preterm </= 1kg -1.00 ± 1.08 preterm 1,001-1,500g -0.29 ± 1.22 preterm BPD subgroup BPD -0.78 ± 1.23 NO BPD -0.29 ± 1.10	z scores term 0.00 ± 1.23 preterm −0.93 ± 1.14 preterm subgroup </= 28 weeks -1.32 ± 1.13 29-36 weeks -0.49 ± 1.01 preterm </= 1kg -1.26 ± 1.19 preterm 1,001-1,500g -0.66 ± 1.04 preterm BPD subgroup BPD -1.18 ± 1.14 NO BPD -0.46 ± 1.01	z scores term 0.39 ± 0.85 preterm −0.22 ± 0.16 preterm subgroup </= 28 weeks -0.46 ± 1.32 29-36 weeks -0.03 ± 0.89 preterm </= 1kg -0.30 ± 1.28 preterm 1,001-1,500g -0.17 ± 1.06 preterm BPD subgroup BPD -0.32 ± 1.29 NO BPD -0.07 ± 0.84	X	X
Choukroun	Mean (SD) - % / Z All: 96.9 (13.4) / -0.45 (1.49) Without BPD: 98.1 (13.0) / -0.31 (1.45) Mild BPD: 95.7 (12.3) / -0.53 (1.32) Mod/severe BPD: 92.6 (18.1) / -1.13 (2.04)	Mean (SD) All: 93.9 (13.3) / -0.92 (1.83) Without BPD: 94.6 (12.7) / -0.82 (1.77) Mild BPD: 93.7 (14.0) / -0.89 (1.82) Mod/severe BPD: 89.9 (15.1) / -1.71 (2.18)	Mean (SD) All: 82.8 (19.8) / -0.59 (0.67) Without BPD: 83.7 (19.2) / -0.57 (0.66) Mild BPD: 82.8 (19.6) / -0.58 (0.65) Mod/severe BPD: 77.1 (24.8) / -0.75 (0.8)	Mean (SD) All: 98.6 (8.0) / -0.26 (1.49) Without BPD: 99.3 (6.7) / -0.14 (1.24) Mild BPD: 98.2 (10.2) / -0.34 (1.86) Mod/severe BPD: 95.5 (9.7) / -0.84 (1.8)	Mean (SD) All: 109.4 (30.6) / 0.25 (1.55) Without BPD: 106.4 (28.8) / 0.11 (1.55) Mild BPD: 113.9 (29.6) / 0.51 (1.36) Mod/severe BPD: 116.9 (43.2) / 0.47 (2)	Mean (SD) - DLCO (raw) / % pred DLCO / KCO All (n = 97): 5.15 (0.80) / 95.2 (14.3) / 1.85 (0.20) Without BPD (n=67): 5.19 (0.80) / 95.2 (14.1) / 1.86 (0.23) Mild BPD (n = 25): 5.10 (0.90) / 95.6 (15.9) / 1.86 (0.17) Mod/severe BPD (n=5): 4.96 (0.60) / 93.8 (11.6) / 1.85 (0.10)
Cristea,	Mean (95% CI) - Z-score -2.7 (-3.3; -1.9)	Mean (95% CI) - Z-score -1.5 (-2.5; -0.4)	Mean (95% CI) - Z-score -3.6 (-4.3; -2.9)	Mean (95% CI) - Z-score -2.3 (-3.1; -1.5)	X	X
Crowther,	Mean (SD), number with data Treatment: -0.92 (1.10), n=98 Control: -0.74 (1.04), n=87	Mean (SD), number with data Treatment: -0.04 (0.94), n=98 Control: 0.02 (1.01), n=87	Mean (SD), number with data Treatment: -1.55 (1.25), n=98 Control: -1.29 (1.19), n=87	Mean (SD), number with data Treatment: -1.54 (0.85), n=98 Control: -1.35 (0.88), n=87	X	X
De Sousa Sena,	mean 75 (6.8)	X	X	X	X	X
Debevec,	Mean (±SD) (L) [% predicted] Preterm: 4.3 ± 0.6 [96%] Term: 4.7 ± 0.8 [98%]	Preterm: 5.1 ± 0.6 [97%] Term: 5.5 ± 0.8 [97%]	X	Preterm: 0.85 ± 0.06 Term: 0.86 ± 0.07	X	X
Devakumar,	FEV1 z score preterm n=48 Mean = -1.22 SD = 0.84 Term n = 697 Mean = -1.12 SD = 0.79	FVC z score Preterm n=48 Mean = -1.15 SD = 0.83 Term n= 697 Mean = -1.04 SD = 0.81	Forced expiratory flow 25-75% z score Preterm n = 48 Mean = -0.59 SD = 0.82 Term n = 697 Mean = -0.46 SD = 0.99	X	X	X
Doyle,	mean (SD) 1997 raw: 1.43 (0.30) z-score: -0.65 (1.30) % pred: 92.0 (15.7) 2005 raw: 1.25 (0.28) z-score: -1.19 (1.17) % pred: 85.4 (14.4)	mean (SD) 1997 raw: 1.65 (0.34) z-score: -0.47 (1.26) % pred: 94.4 (14.9) 2005 raw: 1.50 (0.33) z-score: -0.75 (1.17) % pred: 91.0 (14.2)	mean (SD) 1997 (n = 110) raw: 1.42 (0.46) z-score: -1.30 (1.06) % pred: 72.2 (21.9) 2005 (n = 110) raw: 1.35 (0.45) z-score: -1.27 (1.08) % pred: 72.3 (23.4)	mean (SD) 1997 raw: 0.86 (0.09) z-score: -0.30 (1.33) % pred: 96.8 (10.1) 2005 raw: 0.84 (0.08) z-score: -0.77 (1.20) % pred: 93.4 (9.2)	X	X

© 2022 Kotecha SJ et al. *JAMA Pediatrics.*

Doyle,	mean (SD) - z-score / % pred Caffeine: -1.00 (1.17) / 88.4 (13.7) Control: -1.53 (1.35) / 82.0 (15.8)	mean (SD) - z-score / % pred Caffeine: -0.51 (1.15) / 94.2 (13.2) Control: -0.95 (1.30) / 89.2 (15.0)	mean (SD) - z-score / % pred Caffeine: -1.30 (1.14) / 74.0 (22.1) Control: -1.75 (1.27) / 65.7 (22.7)	mean (SD) - z-score Caffeine: -0.73 (1.37) Control: -0.97 (1.39)	X	X
Flahault,	Z-scores: Median (IQR) Term: 0.06 (-0.65 to 0.54) Preterm: -0.81 (-1.52 to -0.17)	Z-scores: Median (IQR) Term: 0.29 (-0.43 to 0.80) Preterm: -0.11 (0.74 to 0.58)	Not measured	Z-scores: Median (IQR) Term: -0.3 (-0.95 to 0.21) Preterm: -1.09 (-1.92 to -0.23)	X	X
Fortuna,	First visit all mean SD BPD %pred 85.2 (12.9) z-sc0re -1.27 (1.07) no BPD 94.2 (9.9) -0.47 (0.82) controls 105.8 (9.6) 0.5 (0.8) Second visit BPD 80.8 (13) -1.64 (1.1) no BPD 93.7 (11) -0.54 (0.9) controls 106.2 (9) 0.54 (0.8)	First visit all mean (SD) %pred and then z-scores BPD 87.7 (12.9) -1.03 (1.08) no BPD 96.7 (8.99) -0.28 (0.73) controls 102.2 (11.3) 0.18 (0.93) Second visit BPD 87.7 (12.2) -1.06 (1) no BPD 95.9 (9) -0.36 (0.8) controls 103.4 (11) 0.28 (0.9)	first visit all mean (SD) %pred and then z-scores BPD 90.2 (16.63) -1.17 (0.9) no BPD 97.0 (7.42) -0.41 (0.99) controls 111.6 (19) 0.46 (0.78) second visit BPD 64.7 (17.9) -1.77 (1) no BPD 85.8 (20.9) -0.7 (1.1) controls 110.8 (17) 0.45 (0.7)	first visit all mean (SD) percent predicted and them z-score BPD 86.2 (6.51) -0.4 (0.95) no BPD 86.7 (6.3) -0.39 (1.02) controls 91.6 (4.97) 0.57 (0.85) second visit BPD 80.2 (7.1) -1.07 (1) NO BPD 85.35 (5.7) -0.38 (0.9) controls 89.48 (5) 0.44 (0.9)	X	X
Gaffin,	preterm mean 98.5 SD 17.3 Term mean 102.1 SD 18.5	preterm mean 95.3 SD 18.1 Term mean 101.5 SD 17.0	X	preterm mean 0.88 SD 0.07 Term mean 0.87 SD 0.07		
Goncalves,	Median (Min - Max) 87.5% (64.0 - 125.0%)	Median (Min - Max) 93.0% (73.0 - 126.0%)	Median (Min - Max) 79.0% (28.0 - 201.0%)	Median (Min - Max) 86.5% (66.0 - 98.0%)	X	X
Gough,	Mean (SD) - Z-score BPD: -1.41 (1.25) non-BPD: -0.19 (1.16) Term: 0.14 (0.96) Mean (SD) - % predicted BPD: 81.89 (15.90) non-BPD: 96.98 (15.22) Term: 101.16 (11.40)	Mean (SD) BPD: -0.79 (1.14) non-BPD: 0.17 (0.98) Term: 0.12 (0.94) Mean (SD) - % predicted BPD: 90.11 (14.46) non-BPD: 101.85 (12.60) Term: 101.67 (0.83)	Mean (SD) BPD: -1.80 (1.10) non-BPD: -1.13 (1.02) Term: -0.56 (1.45) Mean (SD) - % predicted BPD: 61.63 (23.59) non-BPD: 74.93 (22.06) Term: 90.96 (21.55)	Mean (SD) - Z score BPD: -0.68 (0.22) non-BPD: -0.13 (1.22) Term: 0.34 (0.89) Mean (SD) - % predicted BPD: 94.32 (13.41) non-BPD: 98.45 (10.32) Term: 102.67 (7.18)	X	X
Greenough,	X	home O2 median 89 (73-120) no home O2 95 (69-125)	X	X	X	X
Greenough,	z-score CV N=121 mean SD -0.95 (1.02) HFO N=129 mean SD -0.60 (1.08)	z-score CV N=121 mean SD -0.44 (0.89) HFO N=129 mean SD -0.29 (1.05)	z-score CV N=121 mean SD -1.58 (1.05) HFO N=129 mean SD -1.34 (1.09)	z-score CV N=121 mean SD -1.75 (1.78) HFO N=129 mean SD -1.16 (1.75)	z-score CV N=121 mean SD 0.46 (1.19) HFO N=129 mean SD 0.31 (1.35)	z-score CV N=121 mean SD -1.10 (0.92) HFO N=129 mean SD -0.81 (1.19)
Hadchouel	PRE z score preterm mean -0.6 SD1.3 term -0.1 SD 1.0 preterm BPD -1.4 (1.2) preterm without BPD -0.4 (1.2) POST z score preterm mean -0.2 SD 1.3 term 0.2 SD 0.9 preterm BPD -1.0	z score PRE preterm mean -0.3 SD 1.2 term -0.2 SD 0.8 preterm BPD -1.1 (1.4) preterm without BPD -0.2 (1.1)	PRE z score preterm mean -0.6 SD 1.2 term 0.0 SD 1.2 preterm BPD -1.4 (1.2) preterm without BPD -0.4 (1.2) POST z score preterm mean -0.1 SD 1.2 term 0.5 SD 1.0 preterm BPD -0.9	z score preterm mean -0.3 SD 1.3 term 0.2 SD 1.2 preterm BPD -0.6 (1.4) preterm without BPD -0.2 (1.3) POST z score preterm mean 0.1 SD 1.2 term 0.6 SD 0.9 preterm BPD -0.4	X	% PREDICTED preterm mean113.1 SD 23.1 term 116.6 SD 27.4 preterm BPD 112.5 (21.3) preterm without BPD 113.2 (23.7)

© 2022 Kotecha SJ et al. *JAMA Pediatrics.*

	(1.3) preterm without BPD -0.1 (1.2)		(1.2) preterm without BPD -0.0 (1.1)	(1.1) preterm without BPD 0.2 (1.2)		
Hamon,	z-score preterm mean 0.18 SD 1.05 control mean 0.68 SD 0.81	z-score preterm mean 0.31 SD 1.04 control mean 0.41 SD 0.71	X	z-score preterm mean -0.12 SD 1.36 control mean 0.47 SD 0.81	X	X
Hayden,	pre-bronchodilator mean 81% SD 18% Mean change was 12%	pre-bronchodilator means 88% SD 18%	X	pre-bronchodilator means 83% SD 11%	X	X
Hirata	Data is median and ranges ELBW (n=201) 85.5 (31.6-120) without BPD (N=40) 91.8 (50.4-118) MILD bpd (n=79) 86.2 (48.0-120) moderate BPD (n=64) 85.4 (40.7-113) severe BPD (n=18) 79.9 (31.6-92.7)	Data is median and ranges ELBW (n=201) 90.9 (42.6-125) without BPD (N=40) 93.1 (58.7-125) MILD bpd (n=79) 91.6 (64.1-125) moderate BPD (n=64) 90.8 (61.5-124) severe BPD (n=18) 85.4 (42.6-105)	X	Data is median and ranges ELBW (n=201) 85.1 (40.0-100) without BPD (N=40) 85.1 (54.0-100) MILD bpd (n=79) 85.8 (40.0-100) moderate BPD (n=64) 85.9 (56.0-100) severe BPD (n=18) 79.8 (52.0-96.8)	X	X
Kaczmarczyk	examination one mean 93.8 SD 21.1 examination two 100.7 SD 23.6 examination three preterms 95.54 SD 14.75 examination three terms 102.75 SD 12.7	examination one mean 95.4 SD 18.24 examination two 102.0 SD 18.38 examination three preterms 98.63 SD 14.40 examination three terms 103.79 SD 12.54	PRED examination three preterm 3.65 SD 0.36 examination three terms 3.83 SD 0.22	examination three preterm 0.86 SD 0.01 examination three terms 0.85 SD 0.01	x	x
Karnaushkina,	pre bronchodilator test preterms 79.4 +/- 2.7 LRTI 83.4 +/- 5.4 Healthy 91.2 +/- 4.6 post bronchodilator test preterms 88.8 +/- 5.7 LRTI 88.8 +/- 4.8 Healthy 92.3 +/- 5.2	X	x	X	X	X
Kilbride,	ELBW children 97% +/- 18, heavier preterms 103% +/- 13 NBW 107 +/- 13	NOT STATED	ELBW children 87% +/- 28, heavier preterms 97% +/- 26 NBW 103 +/- 25	X	X	X
Kilbride,	NO group 82 +/- 14 placebo group 83 +/- 17	NO group 86 +/- 15 placebo group 92 +/- 17	NO group 69 +/- 18 placebo group 65 +/- 20	NO group 98 +/- 14 placebo group 95 +/- 7	X	X
Konefal,	Median 95% CI preterm BPD 1.7 95% CI 0.6-4.08 preterm no BPD 1.71 95% CI 0.6-5.03 terms 2.29 95% CI 0.93-5.26 From authors n-CPAP 95,5 ± 18,42 IMV 89,0 ± 20,24 Control 98,3 ± 14,04	FVC expiratory Median 95% CI preterm BPD 1.92 95% CI 0.69-4.33 preterm no BPD 1.88 95% CI 0.69-5.41 terms 2.47 95% CI 1.17-5.56 FVC inspiratory Median 95% CI preterm BPD 1.82 95% CI 0.86-3.79 preterm no BPD 1.77 95% CI 0.86-5.14 terms 2.36	X	X	X	X

© 2022 Kotecha SJ et al. *JAMA Pediatrics.*

		95% CI 1.18-5.45 From authors n-CPAP 85,7 ± 14,66 IMV 79,7 ± 15,58 (n=58) Control 87,5 ± 12,24 (n=90)				
Kotecha,	Mean (SD) Z-score Spirometry at 8-9 25-32w (n=65): -0.461 (0.876) 33-34w (n=79): -0.498 (1.094) 35-36w (n=238): 0.008 (0.908) Term (n=6144): 0.011 (1.000) Spirometry at 14-17 25-32w (n=42): -0.186 (0.850) 33-34w (n=49): -0.022 (1.135) 35-36w (n=129): 0.057 (0.859) Term (n=4105): 0.001 (1.004) Those who had spirometry at 8-9 and 14-17 8-9 / 14-17 25-32w (n=30): -0.441 (0.946) / -0.318 (0.932) 33-34w (n=42): -0.483 (1.039) / -0.079 (1.191) 35-36w (n=107): 0.148 (0.863) / 0.021 (0.860) Term (n=3431): -0.004 (1.006) / 0.010 (1.002)	Mean (SD) Z-score Spirometry at 8-9 25-32w (n=65): -0.204 (0.950) 33-34w (n=79): -0.313 (1.036) 35-36w (n=238): 0.015 (0.950) Term (n=6144): -0.005 (1.001) Spirometry at 14-17 25-32w (n=42): -0.151 (1.034) 33-34w (n=49): 0.169 (0.905) 35-36w (n=129): 0.095 (0.956) Term (n=4105): 0.005 (1.001) Spirometry at 8-9 and 14-17 25-32w (n=30): -0.032 (1.050) / -0.052 (0.918) 33-34w (n=42): -0.090 (0.938) / 0.130 (0.960) 35-36w (n=107): 0.115 (0.941) / 0.045 (0.955) Term (n=3431): -0.019 (1.003) / 0.005 (1.001)	Mean (SD) Z-score Spirometry at 8-9 25-32w (n=65): -0.573 (0.928) 33-34w (n=79): -0.425 (1.095) 35-36w (n=238): -0.010 (0.879) Term (n=6144): 0.013 (1.001) Spirometry at 14-17 25-32w (n=42): -0.438 (0.899) 33-34w (n=49): -0.279 (1.143) 35-36w (n=129): -0.077 (0.877) Term (n=4105): 0.011 (1.001) Spirometry at 8-9 and 14-17 25-32w (n=30): -0.717 (0.944) / -0.572 (0.909) 33-34w (n=42): -0.622 (1.127) / -0.336 (1.173) 35-36w (n=107): 0.086 (0.900) / -0.076 (0.888) Term (n=3431): 0.014 (0.997) / 0.010 (1.001)	Mean (SD) Z-score Spirometry at 8-9 25-32w (n=65): -0.362 (1.183) 33-34w (n=79): -0.312 (1.169) 35-36w (n=238): 0.004 (0.906) Term (n=6144): 0.009 (0.997) Spirometry at 14-17 25-32w (n=42): -0.195 (1.117) 33-34w (n=49): -0.401 (1.104) 35-36w (n=129): -0.079 (1.047) Term (n=4105): 0.010 (0.995) Spirometry at 8-9 and 14-17 25-32w (n=30): -0.605 (1.294) / -0.438 (1.132) 33-34w (n=42): -0.621 (1.272) / -0.432 (1.156) 35-36w (n=107): 0.006 (0.911) / -0.048 (1.076) Term (n=3431): 0.016 (1.003) / 0.003 (0.982)	X	X
Yen-Ping Kung	term 1685.5 (242.7) preterm 1561.2 (248.8)	term 1842.2 (294.5) preterm 1708.8 (283.9)	X	term 91.9 (5.9) preterm 91.6 (5.0)	X	X
Kwinta	ELBW mean 81.3 SD 13 CONTROL mean 95.8 SD 8	ELBW mean 79 SD 13 CONTROL mean 89 SD 7	x	X	X	X
Lasry,	Mean (SD) 74.1 (18.6)	Mean (SD) 80.7 (20.5)	X	Mean (SD) 82.5 (13.6)	X	X
Landry,	mean SD BPD 80 (18) RDS 94 (12) preterm 94 (14) term 98 (19)	BPD 100 (15) RDS 99 (9) preterm 104 (14) term 109 (10)	BPD 68 (26) RDS 92 (19) Preterm 89 (26) term 96 (18)	BPD 70 (12) RDS 81 (9) preterm 79 (7) term 79 (7)	BPD 158 (43) RDS 138 (31) preterm 134 (34) term 125 (26)	BPD 86 (11) RDS 93 (19) Preterm 98 (18) term 99 (10)
Lista,	% (mean ± SD) Pre BDR-VG: 86 ± 14 HFOV: 84 ± 17 Post BDR: VG: 86 ± 14 HFOV: 84 ± 17	Results measured but not given	X	Results measured but not given	% (mean ± SD) Pre BDR-VG: 143 ± 46 HFOV: 194 ± 45 Post BDR: VG: 157 ± 46 HFOV: 150 ± 22	X
Luo,	Median (25th, 75th centiles) FEV1 (mL): 610 (500, 730) FEV1 (z score): -2.57 (-3.50, - 1.78)	Median (25th - 75th centiles) FVC (mL): 654 (543, 773) FVC (z score): -2.77 (-3.46, -1.79)	X	Median (25th, 75th centiles) FEV1/FVC (%) 93 (84, 99) FEV1/FVC (z): -0.36 (-1.66; 0.91)	X	X

© 2022 Kotecha SJ et al. *JAMA Pediatrics.*

MacBean	z-scores median (IQR) No LRTI 0.02 (-0.45 to 0.49) RV LRTI -1.08 (-2.07 to -0.09) RSV LRTI 0.14 (-1.04 to 1.33) Other viral LRTI -1.78 (-3.76 to 0.21)	No LRTI 0.60 (0.16 to 1.04) RV LRTI -0.97 (-1.98 to 0.04) RSV LRTI 0.04 (-1.17 to 1.24) Other vital LRTI -2.61 (-4.63 to -0.59)	X	No LRTI -0.97 (-1.44 to -0.50) RV LRTI -0.25 (-1.22 to 0.71) RSV LRTI 0.09 (-1.06 to 1.24) Other viral LRTI 1.44 (-0.49 to 3.37)	X	X
Maclean	Mean (SD) - z-score BPD (mod/severe): -0.92 (1.36) BPD (no/mild): -0.50 (1.10) Control: 0.09 (1.01)	Mean (SD) - z-score BPD (mod/severe): -0.85 (1.67) BPD (no/mild): 0.35 (1.25) Control: 0.47 (0.90)	Mean (SD) - z-score BPD (mod/severe): -1.6 (1.22) BPD (no/mild): -1.3 (0.87) Control: -0.48 (1.12)	Mean (SD) - z-score BPD (mod/severe): -1.14 (1.5) BPD (no/mild): -1.3 (0.80) Control: -0.57 (1.14)	Mean (SD) - z-score BPD (mod/severe): 0.092 (1.12) BPD (no/mild): -0.16 (0.95) Control: -0.47 (0.97)	Mean (SD) - z-score BPD (mod/severe): -0.49 (0.76) BPD (no/mild): -0.52 (0.82) Control: -0.32 (0.69) KCO BPD (mod/severe): -1.64 (0.89) BPD (no/mild): -1.57 (0.76) Control: -1.06 (0.95)
Molgat-Seon,	Mean (SD) - % predicted / Z-score Term: 103 (2) / -0.13 (1.15) Preterm without BPD: 92 (2) / -0.90 (0.26) Preterm with BPD: 82 (4) / -1.61 (0.29)	Mean (SD) - % predicted / Z-score Term: 103 (2) / 0.34 (0.16) Preterm without BPD: 95 (3) / -0.17 (0.21) Preterm with BPD: 90 (3) / -0.54 (0.24)	Mean (SD) - % predicted / Z-score Term: 94 (3) / -0.46 (0.15) Preterm without BPD: 75 (5) / -1.33 (0.24) Preterm with BPD: 63 (5) / -1.96 (0.28)	Mean (SD) - % predicted / Z-score Term: 95 (1) / -0.76 (1.16) Preterm without BPD: 92 (2) / -1.11 (0.23) Preterm with BPD: 85 (3) / -1.62 (0.26)	Mean (SD) - % predicted / Z-score Term: 87 (6) Preterm without BPD: 111 (9) Preterm with BPD: 126 (9)	N/A
Morata-Alba,	FEV1 % Mean (SD) [min - max] Preterm: 90.2 (13.9) [49.7 - 113.1] Term: 96.0	FVC % Mean (SD) [min - max] Preterm (n = 116): 85.3 (16.6) [44.5 - 114.8] Term (n = 116): 88.3 (14.7) [47.3 - 127.4]	MEF 25-75 % Mean (SD) [min - max] Preterm (n = 116): 100.4 (21.5) [49.1 - 155.6] Term (n = 116): 106.4 (20.7) [45.9 - 164.5]	FEV1/FVC % Mean (SD) [min - max] Preterm (n = 116): 108.4 (8.3) [79.2 - 117.3] Term (n = 116): 112.3 (6.7) [75.7 - 116.3]	Not measured	Not measured
Moreno-Galdo	X	Mean (±SD) Z-score with 95% CI of FVC was: −0.36 ± 0.91 [−0.23 to −0.49],	Mean (±SD) Z-score with 95% CI FEF25-75%: −0.79 ± 1.05 [−0.64 to −0.96].	X	X	X
Morris,	z-scores mean (SD) BPD -1.41 (1.44) no BPD -0.52 (1.23)	z-scores mean (SD) BPD -0.54 (1.36) no BPD 0.13 (1.53)	z-scores mean (SD) BPD -1.88 (1.42) no BPD -1.10 (0.84)	X	X	z-scores mean (SD) BPD -1.50 (1.22) no BPD -0.72 (1.22)
Morsing,	Mean (SD) PT-IUGR -1.1 (1.1) PT-AGA -0.52 (0.96) T-AGA -0.15 (1.21)	Mean (SD) PT-IUGR -0.68 (1.6) PT-AGA -0.20 (1.1) T-AGA 0.01 (0.98)	Mean (SD) PT-IUGR -1.68 (1.1) PT-AGA -1.21 (1.0) T-AGA -0.4 (1.2)	Mean (SD) PT-IUGR -0.99 (1.3) PT-AGA -0.87 (1.19) T-AGA -0.17 (1.2)	X	X
Narayanan	Z-scores (SD) Term 0.15 (0.91) Mild preterm 0.31 (0.87) extreme preterm without CLD -0.37 (1.23) extreme preterm with CLD -0.51 (0.98)	Term 0.04 (1.02) Mild preterm 0.25 (0.81) extreme preterm without CLD -0.16 (1.07) extreme preterm with CLD -0.28 (1.09)	X	X	Term -0.15 (1.00) Mild preterm 0.22 (0.88) extreme preterm without CLD -0.03 (0.85) extreme preterm with CLD 0.24 (1.29)	X
Nasanen-Gilmore	mean (sd) - Z score <34 weeks: -0.43(0.99) 34-37 weeks: -0.10 (1.02) ≥37 weeks: 0.02 (0.93)	mean (sd) <34 weeks: -0.08 (0.89) 34-37 weeks: 0.13 (0.91) ≥37 weeks: 0.14 (0.85)	mean (sd) <34 weeks: 0.00 (2.30) 34-37 weeks: 0.59 (2.50) ≥37 weeks: 0.95 (2.30)	mean (sd) <34 weeks: -0.55 (0.95) 34-37 weeks: -0.38 (0.99) ≥37 weeks: -0.23 (0.84)	X	X
Nixon,	unexposed to ANCS median (5th,95th	unexposed to ANCS median (5th, 95th	X	unexposed to ANCS median (5th, 95th	X	X

© 2022 Kotecha SJ et al. *JAMA Pediatrics.*

	percentile) 95 (73,119) exposed to ANCS 94 (73,121)	percentile) 103 (81,122) exposed to ANCS 102 (84,125)		percentile) 96 (78,107) exposed to ANCS 94 (77,104)		
Nixon,	dex median (5th, 95th percentile) 83 (48, 107) placebo 77 (52, 115)	X	X	X	X	X
Nordlund,	BPD mean (SD) 76.9 (11.6) asthma mean (SD) 84.1 (10.9)	BPD mean (SD) 95.5 (13.1) asthma mean (SD) 98.0 (11.5)	X	BPD mean (SD) 85.3 (9.2) asthma mean (SD) 91.3 (10.9) POST BRONCHODILATOR BPD mean (SD) 92.4 (8.0) asthma mean (SD) 96.4 (1.6)	X	BPD mean (SD) 81.4 (13.6) asthma mean (SD) 88.3 (10.0)
Panagiotounakou,.	Mean (SD) Term n=62: 92.6 (12.38) Preterm n=85: 91.0 (10.1) Preterm with BPD: 89.8 (9.2) Preterm without BPD: 92.2 (10.9)	Mean (SD) Term: 88.1 (11.6) Preterm: 88.1 (10.5) Preterm with BPD: 87.8 (9.3) Preterm without BPD: 88.5 (11.6)	X	X	X	X
Prais,	mean (SD) - % predicted Palivizumab (n = 30): 84 (13) No palivizumab (n = 32): 84 (11)	mean (SD) - % predicted Palivizumab (n = 30): 89 (12) No palivizumab (n = 32): 89 (11)	mean (SD) - % predicted Palivizumab (n = 30): 78 (28) No palivizumab (n = 32): 76 (29)	mean (SD) - raw Palivizumab (n = 30): 0.9 (0.1) No palivizumab (n = 32): 0.9 (0.1)	RV mean (SD) - % predicted Palivizumab (n = 28): 128 (45) No palivizumab (n = 29): 145 (38) RV % TLC mean (SD) - % predicted Palivizumab (n = 28): 0.28 (0.08) No palivizumab (n = 29): 0.31 (0.06)	KCO mean (SD) - % predicted Palivizumab (n = 17): 93 (8) No palivizumab (n = 26): 90 (12)
Praprotnik,	Mean SD Preterm BPD 75.3 (10.7) Preterm no BPD 89.8 (11.2) Term 91.1 (9.5)	Mean SD Preterm BPD 89.1 (12.2) Preterm no BPD 98.5 (11.6) Term 100.1 (9.4)	Mean SD Preterm BPD 58.9 (17.5) Preterm no BPD 82.6 (16.9) Term 90.1 (21.9)	Mean SD Preterm BPD 78.3 (18.6) Preterm no BPD 86.4 (15.9) Term 87.5 (5.0)	X	X
Prenzel,	mean (SD) - Z score BPD (n = 39): -0.75 (1.38) Term (n = 40): 0.52 (0.91)	mean (SD) - Z score BPD (n = 39): -0.38 (1.13) Term (n = 40): 0.01 (0.87)	mean (SD) - Z score BPD (n = 39): -1.48 (1.17) Term (n = 40): 0.25 (1.05)	mean (SD) - Z score BPD (n = 39): -0.63 (1.24) Term (n = 40): 0.98 (1.22)	X	X
Ronkainen	mean (SD) - % predicted All preterm (n = 88): 86.4 (11.8) No BPD (n = 39): 89.4 (11.1) BPD (n = 49): 84.3 (11.4) Term (n = 88): 94.9 (10.1)	mean (SD) - % predicted All preterm (n = 88): 91.0 (11.8) No BPD (n = 39): 92.4 (11.5) BPD (n = 49): 90.4 (11.0) Term (n = 88): 95.0 (10.2)	X	mean (SD) - % predicted All preterm (n = 88): 94.7 (7.8) No BPD (n = 39): 96.1 (6.6) BPD (n = 49): 92.9 (8.5) Term (n = 88): 98.9 (7.3)	RV mean (SD) - % predicted All preterm (n = 88): 144.2 (35.3) No BPD (n = 39): 133.5 (33.4) BPD (n = 49): 153.5 (34.4) Term (n = 88): 150.7 (35.1) RV % TLC mean (SD) - % predicted All preterm (n = 88): 140.5 (35.3) No BPD (n = 39): 128.2 (33.9) BPD (n	mean (SD) - % predicted All preterm (n = 88): 87.6 (13.9) No BPD (n = 39): 91.6 (11.6) BPD (n = 49): 84.6 (14.7) Term (n = 88): 93.7 (12.0)

© 2022 Kotecha SJ et al. *JAMA Pediatrics.*

					= 49): 152.0 (33.0) Term (n = 88): 141.8 (29.3)	
Ruf,	Mean (SD) - % predicted BPD: 83 (22) No-BPD: 97 (11) Control: 105 (8)	Mean (SD) BPD: 95 (17) No-BPD: 101 (13) Control: 106 (7)	X	X	Reported as RV%TLC Mean (SD) BPD: 122 (52) No-BPD: 109 (32) Control: 109 (28)	Mean (SD) BPD: 75 (16) No-BPD: 85 (8) Control: 92 (11)
Simpson,	Mean (SD) Term (n = 48): 0.04 (0.90) Preterm (n = 131): -0.72 (1.13) preterm No BPD (n=52) -0.21 (0.99) preterm BPD (n=70) -1.06 (1.09)	Mean (SD) Term (n = 48): 0.17 (0.95) Preterm (n = 131): 0.13 (1.04) preterm No BPD (n=52) 0.43 (0.86) preterm BPD (n=70) -0.07 (1.11)	Mean (SD) Term (n = 48): -0.42 (0.90) Preterm (n = 131): -1.46 (1.11) preterm No BPD (n=52) -1.06 (1.02) preterm BPD (n=70) -1.75 (1.08)	Mean (SD) Term (n = 48): -0.27 (0.92) Preterm (n = 131): -1.25 (1.01) preterm No BPD (n=52) -1.02 (0.93) preterm BPD (n=70) -1.14 (1.04)	Measured by MBW Mean (SD) Term (n = 50): -0.45 (1.10) Preterm (n = 140): -0.16 (1.23) preterm No BPD (n=55) -0.11 (1.13) preterm BPD (n=85) -0.2 (1.34)	Mean (SD) Term (n = 38): 0.10 (0.97) Preterm (n = 74): 0.28 (1.27) preterm No BPD (n=39) 0.34 (1.34) preterm BPD (n=35) 0.21 (1.19)
Sorensen,	Mean (SD) BPD (n=42): -1.07 (1.18) non-BPD (n=28): -0.43 (1.2) Control (n=38): 0.23 (1.05)	mean (SD) BPD (n=42): -0.23 (0.84) non-BPD (n=28): 0.38 (0.88) Control (n=38): 0.18 (0.92)	mean (SD) z score BPD (n=42): -1.71 (1.23) non-BPD (n=28): -1.29 (1.29) Control (n=38): -0.27 (1.01)	mean (SD) BPD (n=42): -1.33 (1.08) non-BPD (n=28): -1.14 (1.25) Control (n=38): 0.024 (0.86)	RV BPD (n=42): 0.23 (1.03) non-BPD (n=28): 0.29 (0.99) Control (n=38): -0.13 (0.69) TLC BPD (n=42): -0.24 (1.17) non-BPD (n=28): 0.04 (1.13) Control (n=38): -0.22 (1.14) RV/TLC BPD (n=42): 0.25 (1.18) non-BPD (n=28): 0.07 (1.02) Control (n=38): -0.32 (0.66)	DLCO BPD (n=42): -0.87 (1.11) non-BPD (n=28): -0.86 (1.30) Control (n=38): -0.23 (0.93) KCO BPD (n=42): -0.58 (1.30) non-BPD (n=28): -0.86 (0.90) Control (n=38): 0.16 (0.95) *Note: Also has partitioned data, Dm
Teig	Median (IQR-3) z-score preterm -0.72 (-1.08, -0.33) term 1.08 (-0.69, 1.94) percent predicted preterm 91.1 (87.7, 99.9) term 114 (91.3, 122)	median (IQR-3) z-score preterm 0.1 (-1,2, 0.47) term 0.37 (-0.64, 0.93) % predicted preterm 99.8 (88.5, 114) term 105 (92.3, 110)	median (IQR-3) z-score preterm -0.84 (-1.99, -0.26) term 0.61 (-0.41, 1.16) % predicted preterm 73.4 (57.2, 93.1) term 116 (97.5, 130)	median (IQR-3) z-score preterm -0.33 (-0.97, -0.22) term 0.2 (-0.27, 1.41) % predicted preterm 97 (96-104) term 101 (98-105)	X	X
Thunqvist,	z-score preterm mean (SD) -0.72 (1.0) term 0.41 (1.0) severe BPD n= 17 -1.24 (1.21) moderate BP -0.62 (1.20)	z-score preterm mean (SD) -0.44 (1.0) term 0.3 (1.0) severe BPD n= 17 -0.65 (1.17) moderate BP -0.42 (1.15)	X	z-score preterm mean (SD) -0.43 (1.1) term 0.2 (1.14) severe BPD n= 17 -1.03 (1.15) moderate BP -0.30 (1.14) % preterm 86 (8.3) term 90 (7.4) severe BPD 82.6 (8.2) moderate BP 87.8 (7.8)	X	X
Thunqvist,	z-score 8y Preterm females (46) mean (SD) 0.19 (1.05) Terms females (811) 0.5 (0.94) preterm males (53) 0.26 (0.87) term males (753) 0.36 (0.93) z-score 16y Preterm females (59) mean (SD) -0.32 (1.03)	only given in mls	X	% 8y Preterm females (46) mean (SD) 86 (6) Terms females (811) 87 (5) preterm males (53) 83 (5) term males (753) 85 (6) % 16y Preterm females (59) mean (SD) 84 (6) Terms females (896) 87 (6)	X	X

© 2022 Kotecha SJ et al. *JAMA Pediatrics.*

	Terms females (896) -0.01 (0.89) preterm males (47) -0.39 (1.00) term males (790) -0.01 (0.95)			preterm males (47) 81 (5) term males (790) 84 (7)		
Tukova,	Z-score Budenoside mean -0.591 SD 0.96 Placebo mean -0.615 SD 1.15	Z-score Budenoside mean -0.286 SD 0.71 Placebo mean 0.043 SD 0.77	Z-score Budenoside mean -1.004 SD 1.06 Placebo mean -1.458 SD 0.95	Z-score Budenoside mean -0.376 SD 1.46 Placebo mean -0.983 SD 1.19	X	X
Turner, Data from authors						
Um-Bergstrtom,	MEDIAN IQR RANGE Z-SCORES BPD -0.94 (-1.57, -0.08) preterm 0.28 (-0.27, 1.18) asthma 0.14 (-0.52,0.77) Terms 0.78 (-0.03,1.26) % PRED BPD 88.9 (81.4, 99.1) preterm 103.3 (96.9, 113.3) Asthma 101.7 (94.1, 109.0) Terms 108.9 (99.7,114.1)	MEDIAN IQR RANGE Z-SCORES BPD -0.32 (-1.24, 0.14) preterm -0.26 (-0.64,0.60) asthma 0.13 (-0.48, 0.85) Terms 0.37 (-0.18,0.85) % PRED BPD 96 (85.6, 101.7) preterm 96.9 (92.2, 107.5) Asthma 101.6 (94.5, 110.4) Terms 104.6 (97.9, 109.8)	X	MEDIAN IQR RANGE Z-SCORES BPD -0.67 (-1.86, 0.18) preterm 0.82 (0.13, 1.33) asthma -0.20 (-0.43, 0.33) Terms 0.42 (-0.26, 0.89)	MEDIAN IQR RANGE % PRED BPD 88 (60.5, 99.5) preterm 79 (65,132) Asthma 72.5 (52,96) Terms 84.5 (68.5, 105.5)	MEDIAN IQR RANGE % PRED BPD 67 (64, 73) preterm73 (65,83) Asthma 81.5 (75,91) Terms 86.5 (80,96)
Vardar-Yagli,	BPD 78.0 +/- 21.2 controls 98.0 +/- 11.7	BPD 98.0 +/- 14.1 controls 98.3 +/- 10.5	BPD 57.0 +/- 25.9 controls 101.5 +/- 20.8	BPD 73.7 +/- 13.1 controls 91.1 +/- 5.9	X	X
Vollsaeter,	mean (95% CI) Control: -0.31 (-0.57, -0.04) All EP: -0.65 (-0.90, -0.41) non-BPD: -0.56 (-0.91, -0.21) BPD: -0.73 (-1.10, -0.37)	mean (95% CI) Control: -0.16 (-0.42, 0.09) All EP: -0.17 (-0.41, 0.07) non-BPD: -0.17 (-0.48, 0.13) BPD: -0.17 (-0.54, 0.20)	mean (95% CI) Control: -0.53 (-0.79, -0.27) All EP: -1.14 (-1.39, -0.89) non-BPD: -1.04 (-1.40, -0.68) BPD: -1.22 (-1.58, -0.87)	mean (95% CI) Control: -0.30 (-0.54, -0.05) All EP: -0.80 (-1.07, -0.54) non-BPD: -0.69 (-1.06, -0.31) BPD: -0.90 (-1.29, -0.52)	mean (95% CI) Control: 0.27 (-0.09, 0.63) All EP: 0.003 (-0.30, 0.30) non-BPD: 0.05 (-0.40, 0.51) BPD: -0.04 (-0.47, 0.38)	mean (95% CI) Control: 88.2 (84.3, 91.7) All EP: 86.5 (80.6, 92.4) non-BPD: 87.4 (81.4, 93.3) BPD: 85.7 (75.0 96.4)
Vollstaeer,	mean (95% CI) 10y control: -0.054 (-0.31 to 0.20) preterm no BPD: -0.25 (-0.75 to 0.24) preterm mild BPD: -0.75 (-1.15 to -0.35) preterm m/s BPD: -1.41 (-1.84 to -0.98) 18y control: 0.22 (-0.16 to 0.60) preterm no BPD: -0.41 (-1.19 to 0.38) preterm mild BPD: -1.24 (-1.76 to -0.71) preterm m/s BPD: -1.36 (-2.08 to -0.64)	mean (95% CI) 10y control: -0.12 (-0.38 to 0.14) preterm no BPD: -0.38 (-0.89 to 0.14) preterm mild BPD: -0.63 (-1.04 to -0.22) preterm m/s BPD: -0.61 (-1.05 to -0.16) 18y control: -0.16 (-0.58 to 0.27) preterm no BPD: -0.043 (-0.92 to 0.84) preterm mild BPD: -1.30 (-1.89 to -0.71) preterm m/s BPD: -1.07 (-1.87 to -0.25)	mean (95% CI) 10y control: -0.16 (-0.46 to 0.14) preterm no BPD: -0.36 (-0.95 to 0.23) preterm mild BPD: -0.84 (-1.31 to -0.37) preterm m/s BPD: -1.84 (-2.35 to -1.33) 18y control: 0.47 (0.18 to 0.77) preterm no BPD: -0.63 (-1.25 to -0.01) preterm mild BPD: -0.76 (-1.17 to -0.34) preterm m/s BPD: -1.19 (-1.76 to -0.62)	mean (95% CI) 10y control: 0.009 (-0.32 to 0.33) preterm no BPD: 0.22 (-0.42 to 0.86) preterm mild BPD: -0.18 (-0.70 to 0.33) preterm m/s BPD: -1.34 (-1.89 to -0.78) 18y control: 0.53 (0.21 to 0.85) preterm no BPD: -0.55 (-1.21 to 0.12) preterm mild BPD: 0.037 (-0.40 to 0.48) preterm m/s BPD: -0.42 (-1.04 to 0.19)	X	X
Vrijlandt,	MLP % pred mean (SD) 92 (12) z-score mean (SD) -0.6 (1.0) Terms % pred mean (SD) 97 (10) z-score mean (SD) -0.2 (0.8)	MLP % pred mean (SD) 91 (12) z-score mean (SD) -0.72 (1.2) Terms % pred mean (SD) 94 (10) z-score mean (SD) -0.49 (1)	X	X	X	X

© 2022 Kotecha SJ et al. *JAMA Pediatrics.*

Winck,	z score control mean 0.71 ± SD 1.12 VLBW MEAN 0.40 ± SD 1.62	z score control mean 0.83 ± SD 1.03 VLBW MEAN 0.66 ± SD 1.44	z score control mean -0.69 ± SD 1.04 VLBW MEAN - 0.14 ± SD 1.37	z score control mean -0.23 ± SD 0.95 VLBW MEAN - 0.38 ± SD 1.13	X	X
Yaacoby-Bianu,	medians (25th-75th percentile) late preterms 0.03 (-1.10-0.45) terms 0.36 (-0.30-0.76)	medians (25th-75th percentile) Late preterms - 0.32 (-0.13-0.4) terms 0.16 (-0.13-0.54)	medians (25th-75th percentile) late preterms - 0.13 (-1.04-0.51) terms 0.25 (-0.53-0.78)	medians (25th-75th percentile) late preterms 0.18 (-0.59-0.74) terms 0.04 (-0.63-1.02)	X	X
Yang,	z-score mean (SD) VLBW - 0.67 (1.2) term -0.13 (1.17) VLBW BPD -1.34 (1.41) n=46 VLBW no BPD -0.5 (1.08) n=178	z-score mean (SD) VLBW 0.18 (0.99) term 0.31 (1.03) VLBW BPD -0.14 (1.10) n=46 VLBW no BPD 0.26 (0.95) n=178	z-score mean (SD) VLBW - 1.29 (1.28) term -0.52 (1.18) VLBW BPD -1.9 (1.45) n=46 VLBW no BPD - 1.13 (1.19) n=178	z-score mean (SD) VLBW - 1.21 (1.13) term 0.65 (1.01) VLBW BPD -1.67 (1.28) n=46 VLBW no BPD - 1.09 (1.06) n=178	z-score mean (SD) VLBW - 0.8 (0.98) n=226 term - 1.22 (0.9) VLBW BPD -0.33 (1.23) n=46 VLBW no BPD - 0.92 (0.87) n=178	z-score mean (SD) VLBW - 0.71 (1.01) n=224 term - 0.11 (0.79) n=97 VLBW BPD -0.98 (1.06) n=46 VLBW no BPD -0.64 (0.99) n=178
Hacking	Preterm 90.9 (SD) 15.4<br>Control 102.5 (15.5)<br>BPD -1.19 (1.25)<br>No BPD -0.43 (1.09)	Preterm (SD) 93.3 (15.1)<br>Control 101.6 (14.8)<br>BPD -1.02 (1.26)<br>No BPD -0.23 (1.02)	Preterm 71.6 (SD) 22.2<br>Contro 93 (26.4)l<br>BPD -1.7 (1.11)<br>No BPD -1.12 (1.03)	Preterm 96.6 (SD) 10.1<br>Control 98.4 (12.2)<br>BPD -0.31 (1.39)<br>No BPD -0.29 (1.24)	X	X
Bui	Very Preterm 28-<34 weeks Mean (SD)<br>Post-BD FEV1 % predicted 97.2(16.9)<br>Post-BD FEV1 z-score -0.18(1.2)<br>Pre-BD FEV1 % predicted 92.5(18.8)<br>Pre-BD FEV1 z-score -0.52(1.3)<br><br>Late Preterm 34-<37 weeks<br>Post-BD FEV1 % predicted 102.6(12.8)<br>Post-BD FEV1 z-score 0.20(0.9)<br>Pre-BD FEV1 % predicted 98.9(13.8)<br>Pre-BD FEV1 z-score -0.06(1.0)<br>Term<br>Post-BD FEV1 % predicted 101.6(23.9)<br>Post-BD FEV1 z-score 0.15 (2.0)<br>Pre-BD FEV1 % predicted 98.8(29.3)	X	X	Very pretem 28-<34 weeks mean (SD)<br>Post-BD FEV1/FVC 0.76(0.09)<br>Pre-BD FEV1/FVC 0.74(0.1)<br>Late preterm (34-<37 weeks)<br>Post-BD FEV1/FVC 0.79(0.05)<br>Pre-BD FEV1/FVC 0.77(0.06)<br>Term<br>Post-BD FEV1/FVC 0.79(0.06)<br>Pre-BD FEV1/FVC 0.77(0.06)<br>Preterm <37 weeks<br>Post-BD FEV1/FVC 0.78(0.06)<br>Pre-BD FEV1/FVC 0.76(0.07)	X	X

© 2022 Kotecha SJ et al. *JAMA Pediatrics.*

	Pre-BD FEV1 z-score<br>-0.05(2.6)<br>Preterm <37 weeks<br>Post-BD FEV1 % predicted<br>101.5 (13.9)<br>Post-BD FEV1 z-score<br>0.12 (1.0)<br>Pre-BD FEV1 % predicted<br>97.5(15.2)<br>Pre-BD FEV1 z-score<br>-0.16(1.1)					

© 2022 Kotecha SJ et al. *JAMA Pediatrics.*

eReferences

1. Hagman, C., et al., *Perinatal inflammation relates to early respiratory morbidity and lung function at 12 years of age in children born very preterm.* Acta Paediatr, 2021. **110**(7): p. 2084-2092.
2. Hayden, L.P., ; Hersh, C. P., ; Gaffin, J. M., ; Rhein, L. M., ; Sheils, C. A., *Abnormal childhood lung function in former preterm infants: Who is at risk?* Pediatric Pulmonology, 2018. **53 (Supplement 1)**: p. S87-S88.
3. Vrijlandt, E., ; Reijneveld, S., ; Aris-Meijer, J., ; Bos, A., *Few symptoms, almost normal lung function and good exercise capacity in adolescents born moderately preterm: Findings from a community-based cohort.* Pediatric Pulmonology, 2018. **53 (Supplement 1)**: p. S131-S132.
4. Arigliani, M., ; Valentini, E., ; Stocco, C., ; De Pieri, C., ; Castriotta, L., ; Barbato, V., ; Cuberli, E., ; Orsaria, M., ; Cattarossi, L., ; Cogo, P., *Regional ventilation inhomogeneity in survivors of extremely preterm birth.* Pediatric Pulmonology, 2020. **55**(6): p. 1366-1374.
5. Arroyas Sanchez, M., ; Valdeolmillos Padrino, E., ; Quevedo Teruel, S. J., ; Rayo Fernandez, A. I., ; Garcia Garcia, M., ; Gonzalez Carrasco, E., *Bronchopulmonary dysplasia and its long term morbidity.* Journal of Perinatal Medicine. Conference: 12th World Congress of Perinatal Medicine, 2015. **43**(SUPPL. 1).
6. Bar-Yoseph, R., ; Haddad, J., ; Hanna, M., ; Kessel, I., ; Kugelman, A., ; Hakim, F., ; Bentur, L., *Long term follow-up of Palivizumab administration in children born at 29-32 weeks of gestation.* Respiratory Medicine, 2019. **150**: p. 149-153.
7. Bozzetto, S., ; Carraro, S., ; Tomasi, L., ; Berardi, M., ; Zanconato, S., ; Baraldi, E., *Health-related quality of life in adolescent survivors of bronchopulmonary dysplasia.* Respirology, 2016. **21**(6): p. 1113-1117.
8. Cardoen, F., ; Vermeulen, François, ; Proesmans, Marijke, ; Moens, Marleen, ; De Boeck, Kris, *Lung function evolution in children with old and new type bronchopulmonary dysplasia: a retrospective cohort analysis.* European Journal of Pediatrics, 2019. **178**(12): p. 1859-1866.
9. Cazzato, S., ; Ridolfi, L., ; Bernardi, F., ; Faldella, G., ; Bertelli, L., *Lung function outcome at school age in very low birth weight children.* Pediatric Pulmonology, 2013. **48**(8): p. 830-837.
10. Chang, H.Y., ; Chang, J. H., ; Chi, H., ; Hsu, C. H., ; Lin, C. Y., ; Jim, W. T., ; Peng, C. C., *Reduced Lung Function at Preschool Age in Survivors of Very Low Birth Weight Preterm Infants.* Frontiers in Pediatrics, 2020. **8**.
11. Choukroun, M.L., ; Feghali, H., ; Vautrat, S., ; Marquant, F., ; Nacka, F., ; Leroy, V., ; Demarquez, J. L., ; Fayon, M. J., *Pulmonary outcome and its correlates in school-aged children born with a gestational age ≤ 32 weeks.* Respiratory Medicine, 2013. **107**(12): p. 1966-1976.
12. Cristea, A., ; Ackerman, V., ; Davis, S. D., *Physiologic findings in children previously ventilator dependent due to bronchopulmonary dysplasia.* American Journal of Respiratory and Critical Care Medicine. Conference: American Thoracic Society International Conference, ATS, 2013. **187**(MeetingAbstracts).
13. Crowther, C.A., ; Anderson, Peter J., ; McKinlay, Christopher J. D., ; Harding, Jane E., ; Ashwood, Pat J., ; Haslam, Ross R., ; Robinson, Jeffery S., ; Doyle, Lex W., ; Actords Follow-up Group, *Mid-Childhood Outcomes of Repeat Antenatal Corticosteroids: A Randomized Controlled Trial.* 2016.
14. De Sousa Sena, R., ; Tremblay, G., ; MacMillan, N., ; Tanja, T., ; Jean, B., ; Landry, J., *Physical activity in daily life: A new outcome to evaluate young adult survivors of bronchopulmonary dysplasia?* European Respiratory Journal. Conference: European Respiratory Society Annual Congress, 2013. **42**(SUPPL. 57).
15. Debevec, T., ; Pialoux, Vincent, ; Millet, Gregoire P., ; Martin, Agnes, ; Mramor, Minca, ; Osredkar, Damjan, *Exercise Overrides Blunted Hypoxic Ventilatory Response in Prematurely Born Men.* Frontiers in Physiology, 2019. **10**: p. 437.

© 2022 Kotecha SJ et al. *JAMA Pediatrics.*

16. Devakumar, D., ; Stocks, J., ; Ayres, J. G., ; Kirkby, J., ; Yadav, S. K., ; Saville, N. M., ; Devereux, G., ; Wells, J. C. K., ; Manandhar, D. S., ; Costello, A., ; Osrin, D., *Effects of antenatal multiple micronutrient supplementation on lung function in mid-childhood: Follow-up of a double-blind randomised controlled trial in Nepal.* European Respiratory Journal, 2015. **45**(6): p. 1566-1575.
17. Doyle, L.W., ; Carse, Elizabeth, ; Adams, Anne-Marie, ; Ranganathan, Sarath, ; Opie, Gillian, ; Cheong, Jeanie L. Y., ; Victorian Infant Collaborative Study, Group, *Ventilation in Extremely Preterm Infants and Respiratory Function at 8 Years.* New England Journal of Medicine, 2017. **377**(4): p. 329-337.
18. Doyle, L.W., ; Ranganathan, S., ; Cheong, J. L. Y., *Neonatal caffeine treatment and respiratory function at 11 years in children under 1, 251 g at Birth.* American Journal of Respiratory and Critical Care Medicine, 2017. **196**(10): p. 1318-1324.
19. Flahault, A., ; Paquette, Katryn, ; Fernandes, Rafael Oliveira, ; Delfrate, Jacques, ; Cloutier, Anik, ; Henderson, Melanie, ; Lavoie, Jean-Claude, ; Masse, Benoit, ; Nuyt, Anne Monique, ; Luu, Thuy Mai, ; Hapi collaborating group*, *Increased Incidence but Lack of Association Between Cardiovascular Risk Factors in Adults Born Preterm.* Hypertension, 2020. **75**(3): p. 796-805.
20. Fortuna, M., ; Carraro, S., ; Temporin, E., ; Berardi, M., ; Zanconato, S., ; Salvadori, S., ; Lago, P., ; Frigo, A. C., ; Filippone, M., ; Baraldi, E., *Mid-childhood lung function in a cohort of children with "new bronchopulmonary dysplasia".* Pediatric Pulmonology, 2016. **51**(10): p. 1057-1064.
21. Gaffin, J.M., ; Hauptman, M., ; Petty, C. R., ; Haktanir-Abul, M., ; Gunnlaugsson, S., ; Lai, P. S., ; Baxi, S. N., ; Permaul, P., ; Sheehan, W. J., ; Wolfson, J. M., ; Coull, B. A., ; Gold, D. R., ; Koutrakis, P., ; Phipatanakul, W., *Differential Effect of School-Based Pollution Exposure in Children With Asthma Born Prematurely.* Chest, 2020. **158**(4): p. 1361-1363.
22. Goncalves, C., ; Wandalsen, G., ; Lanza, F., ; Goulart, A. L., ; Sole, D., ; dos Santos, A., *Repercussions of preterm birth on symptoms of asthma, allergic diseases and pulmonary function, 6-14 years later.* Allergologia Et Immunopathologia, 2016. **44**(6): p. 489-496.
23. Gough, A., ; Linden, M., ; Spence, D., ; Patterson, C. C., ; Halliday, H. L., ; McGarvey, L. P. A., *Impaired lung function and health status in adult survivors of bronchopulmonary dysplasia.* European Respiratory Journal, 2014. **43**(3): p. 808-816.
24. Greenough, A., ; Alexander, J., ; Boorman, J., ; Chetcuti, P. A. J., ; Cliff, I., ; Lenney, W., ; Morgan, C., ; Shaw, N. J., ; Sylvester, K. P., ; Turner, J., *Respiratory morbidity, healthcare utilisation and cost of care at school age related to home oxygen status.* European Journal of Pediatrics, 2011. **170**(8): p. 969-975.
25. Greenough, A., ; Peacock, J., ; Zivanovic, S., ; Alcazar-Paris, M., ; Lo, J., ; Marlow, N., ; Calvert, S., *United Kingdom Oscillation Study: long-term outcomes of a randomised trial of two modes of neonatal ventilation.* Health Technology Assessment, 2014. **18**(41): p. 1-+.
26. Hadchouel, A., ; Rousseau, J., ; Roze, J. C., ; Arnaud, C., ; Bellino, A., ; Couderc, L., ; Marret, S., ; Mittaine, M., ; Pinquier, D., ; Verstraete, M., ; Ancel, P. Y., ; Delacourt, C., *Association between asthma and lung function in adolescents born very preterm: Results of the EPIPAGE cohort study.* Thorax., 2018. **31**.
27. Hamon, I., ; Varechova, S., ; Vieux, R., ; Ioan, I., ; Bonabel, C., ; Schweitzer, C., ; Hascoet, J. M., ; Marchal, F., *Exercise-induced bronchoconstriction in school-Age children born extremely preterm.* Pediatric Research, 2013. **73**(4): p. 464-468.
28. Hayden, L.P., ; Gaffin, J. M., ; Sheils, C. A., ; Hersh, C. P., ; Rhein, L. M., *Predictors of lung function in former preterm infants: A boston children's cohort.* American Journal of Respiratory and Critical Care Medicine. Conference: American Thoracic Society International Conference, ATS, 2018. **197**(MeetingAbstracts).

© 2022 Kotecha SJ et al. *JAMA Pediatrics.*

29. Hirata, K., ; Nishihara, Masahiro, ; Shiraishi, Jun, ; Hirano, Shinya, ; Matsunami, Katsura, ; Sumi, Kiyoaki, ; Wada, Norihisa, ; Kawamoto, Yutaka, ; Nishikawa, Masanori, ; Nakayama, Masahiro, ; Kanazawa, Tadahiro, ; Kitajima, Hiroyuki, ; Fujimura, Masanori, *Perinatal factors associated with long-term respiratory sequelae in extremely low birthweight infants.* Archives of Disease in Childhood -- Fetal & Neonatal Edition, 2015. **100**(4): p. F314-9.
30. Kaczmarczyk, K., ; Wiszomirska, I., ; Szturmowicz, M., ; Magiera, A., ; Blazkiewicz, M., *Are preterm-born survivors at risk of long-term respiratory disease?* Therapeutic Advances in Respiratory Disease, 2017. **11**(7): p. 277-287.
31. Karnaushkina, M.A., ; Strutinskaya, A. D., ; Ovsyannikov, D. Y., *Prematurity and early childhood infection of lower respiratory tract as risk factors of developing chronic obstructive bronchopulmonary pathology in adults.* Sovremennye Tehnologii v Medicine, 2017. **9**(1): p. 129-133.
32. Kilbride, H., ; Dinakar, C., ; Carver, T., ; Gauldin, C., ; Teson, K., ; Gelatt, M., ; Sabath, R., *Pulmonary function, oxygen consumption, and exhaled nitric oxide measures for extremely low birth weight, heavier preterm, and term children.* Journal of Investigative Medicine, 2012. **60 (1)**: p. 239.
33. Kilbride, H., ; Escobar, H., ; Holmes, A., ; Teson, K., ; Truog, W., *Childhood Pulmonary Function, Exercise Capacity, and Exhaled Nitric Oxide Levels: Outcomes following Neonatal Treatment with Inhaled Nitric Oxide to Prevent Bronchopulmonary Dysplasia.* American Journal of Perinatology, 2019. **36**(4): p. 360-365.
34. Konefal, H., ; Czeszynska, M. B., ; Merritt, T. A., *School-age spirometry in survivors of chronic lung disease of prematurity in the surfactant era.* Ginekologia Polska, 2013. **84**(4): p. 286-292.
35. Kotecha, S.J., ; John Watkins, W., ; Paranjothy, S., ; Dunstan, F. D., ; John Henderson, A., ; Kotecha, S., *Effect of late preterm birth on longitudinal lung spirometry in school age children and adolescents.* Thorax., 2011. **27**.
36. Kung, Y.P., ; Lin, C. C., ; Chen, M. H., ; Tsai, M. S., ; Hsieh, W. S., ; Chen, P. C., *Intrauterine exposure to per- and polyfluoroalkyl substances may harm children's lung function development.* Environmental Research, 2021. **192**.
37. Kwinta, P., ; Lis, G., ; Klimek, M., ; Grudzien, A., ; Tomasik, T., ; Poplawska, K., ; Pietrzyk, J. J., *The prevalence and risk factors of allergic and respiratory symptoms in a regional cohort of extremely low birth weight children (<1000 g).* Italian journal of pediatrics, 2013. **39**.
38. Lasry A, Kavabushi P, Canakis AM, Luu TM, Nuyt AM, Perreault T, Simoneau J, Landry J, Altit G. Cardiopulmonary Function Abnormalities in Cohort of Adults following Bronchopulmonary Dysplasia as Preterm Infants. Am J Perinatol. 2021 Jan 17. doi: 10.1055/s-0040-1722604. Epub ahead of print. PMID: 33454944..
39. Landry, J.S., ; Tremblay, G. M., ; Li, P. Z., ; Wong, C., ; Benedetti, A., ; Taivassalo, T., *Lung function and bronchial hyperresponsiveness in adults born prematurely: A cohort study.* Annals of the American Thoracic Society, 2016. **13**(1): p. 17-24.
40. Lista, G., ; Castoldi, F., ; Bianchi, S., ; Lupo, E., ; Cavigioli, F., ; Farolfi, A., ; Bersanini, C., ; Ferrerio, E., *Lung function and respiratory health at school age in ventilated very low birth weight infants.* Indian Journal of Pediatrics, 2014. **81**(3): p. 275-278.
41. Luo, H.J., ; Wang, L. Y., ; Chen, P. S., ; Hsieh, W. S., ; Hsu, C. H., ; Peng, S., ; Jeng, S. F., *Neonatal respiratory status predicts longitudinal respiratory health outcomes in preterm infants.* Pediatric Pulmonology, 2019. **54**(6): p. 814-821.
42. MacBean, V., ; Drysdale, S. B., ; Yarzi, M. N., ; Peacock, J. L., ; Rafferty, G. F., ; Greenough, A., *Respiratory viral infections in infancy and school age respiratory outcomes and healthcare costs.* Pediatric Pulmonology, 2018. **53**(3): p. 342-348.

© 2022 Kotecha SJ et al. *JAMA Pediatrics.*

43. Maclean, J.E., ; Dehaan, K., ; Fuhr, D., ; Hariharan, S., ; Kamstra, B., ; Hendson, L., ; Adatia, I., ; Majaesic, C., ; Lovering, A. T., ; Thompson, R. B., ; Nicholas, D., ; Thebaud, B., ; Stickland, M. K., *Altered breathing mechanics and ventilatory response during exercise in children born extremely preterm.* Thorax, 2016. **71**(11): p. 1012-1019.
44. Molgat-Seon, Y., ; Dominelli, P. B., ; Peters, C. M., ; Guenette, J. A., ; William Sheel, A., ; Gladstone, I. M., ; Lovering, A. T., ; Duke, J. W., *Analysis of maximal expiratory flow-volume curves in adult survivors of preterm birth.* American Journal of Physiology - Regulatory Integrative and Comparative Physiology, 2019. **317**(4): p. R588-R596.
45. Morata-Alba, J., ; Romero-Rubio, M. T., ; Castillo-Corullón, S., ; Escribano-Montaner, A., *Respiratory morbidity, atopy and asthma at school age in preterm infants aged 32–35 weeks.* European Journal of Pediatrics, 2019. **178**(7): p. 973-982.
46. Moreno-Galdo, A., ; Perez-Yarza, E. G., ; Ramilo, O., ; Rubi, T., ; Escribano, A., ; Torres, A., ; Sardon, O., ; Oliva, C., ; Perez, G., ; Cortell, I., ; Rovira-Amigo, S., ; Pastor-Vivero, M. D., ; Perez-Frias, J., ; Velasco, V., ; Torres, J., ; Figuerola, J., ; Barrio, M. I., ; Garcia-Hernandez, G., ; Mejias, A., ; Sareprem Investigators, *Recurrent wheezing during the first 3 years of life in a birth cohort of moderate-to-late preterm infants.* Pediatric Allergy and Immunology, 2020. **31**(2): p. 124-132.
47. Morris, S., ; Harris, C., ; Lunt, A., ; Peacock, J., ; Greenough, A., *Lung function at follow-up of very prematurely born young people-impact of bronchopulmonary dysplasia.* Archives of Disease in Childhood, 2018. **103 (Supplement 1)**: p. A183-A184.
48. Morsing, E., ; Gustafsson, P., ; Brodszki, J., *Lung function in children born after foetal growth restriction and very preterm birth.* Acta Paediatrica, International Journal of Paediatrics, 2012. **101**(1): p. 48-54.
49. Narayanan, M., ; Beardsmore, C. S., ; Owers-Bradley, J., ; Dogaru, C. M., ; Mada, M., ; Ball, I., ; Garipov, R. R., ; Kuehni, C. E., ; Spycher, B. D., ; Silverman, M., *Catch-up Alveolarization in Ex-Preterm Children Evidence from He-3 Magnetic Resonance.* American Journal of Respiratory and Critical Care Medicine, 2013. **187**(10): p. 1104-1109.
50. Näsänen-Gilmore, P., ; Sipola-Leppänen, M., ; Tikanmä Ki, M., ; Matinolli, H. M., ; Eriksson, J. G., ; Järvelin, M. R., ; Vääräsmä Ki, M., ; Hovi, P., ; Kajantie, E., *Lung function in adults born preterm.* PLoS ONE, 2018. **13**(10).
51. Nixon, P.A., ; Washburn, L. K., ; O'Shea, T. M., *Antenatal steroid exposure and pulmonary outcomes in adolescents born with very low birth weight.* Journal of Perinatology, 2013. **33**(10): p. 806-810.
52. Nixon, P.A., ; Washburn, L. K., ; Mudd, L. M., ; Webb, H. H., ; O'Shea, T. M., *Aerobic fitness and physical activity levels of children born prematurely following randomization to postnatal dexamethasone.* Journal of Pediatrics, 2011. **158**(1): p. 65-70.
53. Nordlund, B., ; James, A., ; Ebersjo, C., ; Hedlin, G., ; Brostrom, E. B., *Differences and similarities between bronchopulmonary dysplasia and asthma in schoolchildren.* Pediatric Pulmonology, 2017. **52**(9): p. 1179-1186.
54. Panagiotounakou, P., ; Sokou, R., ; Gounari, E., ; Konstantinidi, A., ; Antonogeorgos, G., ; Grivea, I. N., ; Daniil, Z., ; Gourgouliannis, K. I., ; Gounaris, A., *Very preterm neonates receiving "aggressive" nutrition and early nCPAP had similar long-term respiratory outcomes as term neonates.* Pediatric Research, 2019. **86**(6): p. 742-748.
55. Prais, D., ; Kaplan, E., ; Klinger, G., ; Mussaffi, H., ; Mei-Zahav, M., ; Bar-Yishay, E., ; Stafler, P., ; Steuer, G., ; Sirota, L., ; Blau, H., *Short-and long-term pulmonary outcome of palivizumab in children born extremely prematurely.* Chest, 2016. **149**(3): p. 801-808.

© 2022 Kotecha SJ et al. *JAMA Pediatrics.*

56. Praprotnik, M., ; Gantar, I. S., ; Lucovnik, M., ; Avcin, T., ; Krivec, U., *Respiratory morbidity, lung function and fitness assessment after bronchopulmonary dysplasia.* Journal of Perinatology, 2015. **35**(12): p. 1037-1042.

57. Prenzel, F., ; Vogel, M., ; Siekmeyer, W., ; Korner, A., ; Kiess, W., ; vom Hove, M., *Exercise capacity in children with bronchopulmonary dysplasia at school age.* Respiratory Medicine, 2020. **171 (no pagination)**(106102).

58. Ronkainen, E., ; Dunder, T., ; Peltoniemi, O., ; Kaukola, T., ; Marttila, R., ; Hallman, M., *New BPD predicts lung function at school age: Follow-up study and meta-analysis.* Pediatric Pulmonology, 2015. **50**(11): p. 1090-1098.

59. Ruf, K., ; Thomas, Wolfgang, ; Brunner, Maximilian, ; Speer, Christian P., ; Hebestreit, Helge, *Diverging effects of premature birth and bronchopulmonary dysplasia on exercise capacity and physical activity - a case control study.* Respiratory Research, 2019. **20**(1): p. N.PAG-N.PAG.

60. Simpson, S.J., ; Logie, K. M., ; O'Dea, C. A., ; Banton, G. L., ; Murray, C., ; Wilson, A. C., ; Pillow, J. J., ; Hall, G. L., *Altered lung structure and function in mid-childhood survivors of very preterm birth.* Thorax, 2017. **72**(8): p. 702-711.

61. Sorensen, J.K., ; Buchvald, F., ; Berg, A. K., ; Robinson, P. D., ; Nielsen, K. G., *Ventilation inhomogeneity and NO and CO diffusing capacity in ex-premature school children.* Respiratory Medicine, 2018. **140**: p. 94-100.

62. Teig, N., ; Allali, M., ; Rieger, C., ; Hamelmann, E., *Inflammatory markers in induced sputum of school children born before 32 completed weeks of gestation.* Journal of Pediatrics, 2012. **161**(6): p. 1085-1090.

63. Thunqvist, P., ; Tufvesson, Ellen, ; Bjermer, Leif, ; Winberg, Anna, ; Fellman, Vineta, ; Domellof, Magnus, ; Melen, Erik, ; Norman, Mikael, ; Hallberg, Jenny, *Lung function after extremely preterm birth-A population-based cohort study (EXPRESS).* Pediatric Pulmonology, 2018. **53**(1): p. 64-72.

64. Thunqvist, P., ; Gustafsson, P. M., ; Schultz, E. S., ; Bellander, T., ; Berggren-Brostrom, E., ; Norman, M., ; Wickman, M., ; Melen, E., ; Hallberg, J., *Lung function at 8 and 16 years after moderate-to-late preterm birth: A prospective cohort study.* Pediatrics, 2016. **137**(4).

65. Tukova, J., ; Smisek, J., ; Zlatohlavkova, B., ; Plavka, R., ; Markova, D., *Early inhaled budesonide in extremely preterm infants decreases long-term respiratory morbidity.* Pediatric Pulmonology, 2020. **55**(5): p. 1124-1130.

66. Turner, S., ; Fielding, S., ; Devereux, G., *First trimester fetal size and prescribed asthma medication at 15 years of age.* European Respiratory Journal, 2018. **51**(2).

67. Um-Bergström, P., ; Hallberg, J., ; Pourbazargan, M., ; Berggren-Broström, E., ; Ferrara, G., ; Eriksson, M. J., ; Nyrén, S., ; Gao, J., ; Lilja, G., ; Lindén, A., ; Wheelock, Å M., ; Melén, E., ; Sköld, C. M., *Pulmonary outcomes in adults with a history of Bronchopulmonary Dysplasia differ from patients with asthma.* Respiratory Research, 2019. **20**(1).

68. Vardar-Yagli, N., ; Inal-Ince, Deniz, ; Saglam, Melda, ; Arikan, Hulya, ; Savci, Sema, ; Calik-Kutukcu, Ebru, ; Ozcelik, Ugur, *Pulmonary and extrapulmonary features in bronchopulmonary dysplasia: a comparison with healthy children.* Journal of Physical Therapy Science, 2015. **27**(6): p. 1761-5.

69. Vollsæter, M., ; Skromme, K., ; Satrell, E., ; Clemm, H., ; Røksund, O., ; Øymar, K., ; Markestad, T., ; Halvorsen, T., *Children Born Preterm at the Turn of the Millennium Had Better Lung Function Than Children Born Similarly Preterm in the Early 1990s.* PLoS ONE, 2015. **10**(12).

70. Vollsaeter, M., ; Roksund, O. D., ; Eide, G. E., ; Markestad, T., ; Halvorsen, T., *Lung function after preterm birth: development from mid-childhood to adulthood.* Thorax, 2013. **68**(8): p. 767-776.

71. Vrijlandt, E., ; Reijneveld, S. A., ; Aris-Meijer, J. L., ; Bos, A. F., *Respiratory Health in Adolescents Born Moderately-Late Preterm in a Community-Based Cohort.* Journal of Pediatrics, 2018. **203**: p. 429-436.

© 2022 Kotecha SJ et al. *JAMA Pediatrics.*

72. Winck, A.D., ; Heinzmann-Filho, Joao Paulo, ; Schumann, Deise, ; Zatti, Helen, ; Mattiello, Rita, ; Jones, Marcus Herbert, ; Stein, Renato Tetelbom, *Growth, lung function, and physical activity in schoolchildren who were very-low-birth-weight preterm infants.* Jornal Brasileiro De Pneumologia: Publicacao Oficial Da Sociedade Brasileira De Pneumologia E Tisilogia, 2016. **42**(4): p. 254-260.
73. Yaacoby-Bianu, K., ; Plonsky, M. T., ; Gur, M., ; Bar-Yoseph, R., ; Kugelman, A., ; Bentur, L., *Effect of late preterm birth on lung clearance index and respiratory physiology in school-age children.* Pediatric Pulmonology, 2019. **54**(8): p. 1250-1256.
74. Yang, J., ; Kingsford, R. A., ; Horwood, J., ; Epton, M. J., ; Swanney, M. P., ; Stanton, J., ; Darlow, B. A., *Lung function of adults born at very low birth weight.* Pediatrics, 2020. **145**(2).
75. Hacking DF, Gibson AM, Robertson C, Doyle LW; Victorian Infant Collaborative Study Group (VICS). Respiratory function at age 8-9 after extremely low birthweight or preterm birth in Victoria in 1997. Pediatr Pulmonol. 2013 May;48(5):449-55. doi: 10.1002/ppul.22619. Epub 2012 Jul 23. PMID: 22826206.
76. Aoyama BC, Collaco JM, McGrath-Morrow SA. Predictors of pulmonary function at 6 years ofage in infants with bronchopulmonary dysplasia.PediatricPulmonology. 2021;56:974-981.https://doi.org/10.1002/ppul.25244AOYAMAET AL.|981
77. Arroyas M, Calvo C, Rueda S, Esquivias M, Gonzalez-Menchen C, Gonzalez-Carrasco E, Garcia-Garcia ML. Asthma prevalence, lung and cardiovascular function in adolescents born preterm. Sci Rep. 2020 Nov 12;10(1):19616. doi: 10.1038/s41598-020-76614-0. PMID: 33184335; PMCID: PMC7661536.
78. Durlak W, Klimek M, Wroński M, Trybulska A, Kwinta P. Multimodal longitudinal respiratory function assessment in very low birth weight 7-year-old children. Adv Med Sci. 2021 Mar;66(1):81-88. doi: 10.1016/j.advms.2020.12.006. Epub 2021 Jan 7. PMID: 33421705.
79. Di Filippo P, Giannini C, Attanasi M, Dodi G, Scaparrotta A, Petrosino MI, Di Pillo S, Chiarelli F. Pulmonary Outcomes in Children Born Extremely and Very Preterm at 11 Years of Age. Front Pediatr. 2021 May 25;9:635503. doi: 10.3389/fped.2021.635503. PMID: 34113584; PMCID: PMC8185052.
80. Hart K, Cousins M, Watkins WJ, Kotecha SJ, Henderson AJ, Kotecha S. Association of Early Life Factors with Prematurity-Associated Lung Disease: Prospective Cohort Study. Eur Respir J. 2021 Oct 8:2101766. doi: 10.1183/13993003.01766-2021. Epub ahead of print. PMID: 34588197.
81. Joshi S, Powell T, Watkins WJ, Drayton M, Williams EM, Kotecha S. Exercise-induced bronchoconstriction in school-aged children who had chronic lung disease in infancy. J Pediatr. 2013 Apr;162(4):813-818.e1. doi: 10.1016/j.jpeds.2012.09.040. Epub 2012 Oct 27. Erratum in: J Pediatr. 2013 Jun;162(6):1298. PMID: 23110946.
82. Naples, R. Manifield, J. Chynkiamis, N. Brodlie, M. Fenton, A. C. Harigopal, S. Vogiatzis, I. O'Brien, C. J. Use of optoelectronic plethysmography to assess the ventilatory response to exercise in school-aged children born preterm Pediatric Pulmonology. Conference: 20th International Congress of Pediatric Pulmonology. Virtual. 56(SUPPL 2)
83. Pérez-Tarazona S, Rueda Esteban S, García-García ML, Arroyas Sanchez M, de Mir Messa I, Acevedo Valarezo T, Mesa Medina O, Callejón Callejón A, Canino Calderín EM, Albi Rodriguez S, Ayats Vidal R, Salcedo Posadas A, Costa Colomer J, Domingo Miró X, Berrocal Castañeda M, Villares Porto-Dominguez A; Working Group of Perinatal Respiratory Diseases of the Spanish Society of Pediatric Pulmonology. Respiratory outcomes of "new" bronchopulmonary dysplasia in adolescents: A multicenter study. Pediatr Pulmonol. 2021 May;56(5):1205-1214. doi: 10.1002/ppul.25226. Epub 2020 Dec 22. PMID: 33314679; PMCID: PMC8246560.
84. Santema HY, Stolk J, Los M, Stoel BC, Tsonaka R, Merth IT. Prediction of lung function and lung density of young adults who had bronchopulmonary dysplasia. ERJ Open Res. 2020 Oct 26;6(4):00157-2020. doi: 10.1183/23120541.00157-2020. PMID: 33263029; PMCID: PMC7682661.

© 2022 Kotecha SJ et al. *JAMA Pediatrics.*

85. Vanhaverbeke K, Slaats M, Al-Nejar M, Everaars N, Snoeckx A, Spinhoven M, El Addouli H, Lauwers E, Van Eyck A, De Winter BY, Van Hoorenbeeck K, De Dooy J, Mahieu L, Mignot B, De Backer J, Mulder A, Verhulst S. Functional respiratory imaging provides novel insights into the long-term respiratory sequelae of bronchopulmonary dysplasia. Eur Respir J. 2021 Jun 4;57(6):2002110. doi: 10.1183/13993003.02110-2020. PMID: 33303551.
86. Bui DS, Perret J, Walters EH, Lodge C, Bowatte G, Hamilton GS, Thompson BR, Frith P, Erbas B, Thomas PS, Johns D, Wood-baker R, Hopper J, Davis PG, Abramson MJ, Lowe A, Dharmage SC. Associations between very to moderate preterm and lung function deficits and COPD at age 53: a prospective cohort study over six decades. Lancet Respir Med. (In Press)

© 2022 Kotecha SJ et al. *JAMA Pediatrics.*
